# Exploring the Moderating Effect of Control Group Type on Intervention Effectiveness in School-Based Anxiety and Depression Prevention: Findings from a Rapid Review and Network Meta-analysis

**DOI:** 10.1007/s11121-025-01786-y

**Published:** 2025-02-12

**Authors:** Deborah M. Caldwell, Jennifer C. Palmer, Katie E. Webster, Sarah R. Davies, Hugo Hughes, Joseph Rona, Rachel Churchill, Sarah E. Hetrick, Nicky J. Welton

**Affiliations:** 1 Population Health Sciences, Bristol Medical School, University of Bristol, Bristol, BS8 2PS UK; 2 School of Medicine and Population Health, University of Sheffield, Sheffield, S10 2HQ UK; 3 Centre for Reviews and Dissemination, University of York, York, YO10 5DD UK; 4 Faculty of Medical and Health Sciences, Psychological Medicine, University of Auckland, Auckland, 1023 New Zealand; 5 NIHR Health Protection Research Unit in Behavioural Science and Evaluation, Population Health Sciences, Bristol Medical School, University of Bristol, Bristol, UK

**Keywords:** Child, Adolescence, Mental health, Depression, Anxiety, School, Control group, Comparator, Network meta-analysis, Moderation

## Abstract

Many randomized controlled trials have investigated the role of school-based prevention interventions to reduce symptoms of anxiety and depression in young people. Systematic reviews have subsequently demonstrated a small, beneficial effect of these interventions when compared to a combined control group including usual care, no intervention, or waiting list controls. However, evidence from behavioral science and clinical psychology suggests control group choice may influence the relative effectiveness of non-pharmacological interventions. Here we explored whether separating this combined control group into distinct categories might influence the apparent effectiveness of preventive interventions. After updating an earlier review and network meta-analysis of preventive interventions for anxiety and depression in young people, we considered the impact of alternative control groups on estimates of effectiveness. This analysis was restricted to comparisons with cognitive-behavioral interventions only—the most common intervention used in the included studies. In targeted populations, for both anxiety and depression outcomes, the effect of a cognitive-behavioral intervention was larger when compared to waiting list controls than to usual curriculum, no intervention, or attention controls. For anxiety, the effect of no intervention was also considerably larger than waiting list control (standardized mean difference -0.37 [95% credible interval − 0.66, − 0.11], favoring no intervention). These results suggest that the beneficial effect of preventive school-based interventions previously observed in standard meta-analyses may be an artifact of combining control groups. Although exploratory, these findings indicate the impact of different control groups may vary considerably and should be taken into account when interpreting the effectiveness of interventions.

**Supplementary Information:**

The online version contains supplementary material available at 10.1007/s11121-025-01786-y.

## Introduction

Globally, depressive and anxiety disorders are estimated to be the fourth and sixth leading causes of disability-adjusted life years in young people aged 10–24 years old (Vos et al., 2020). Between 1990 and 2019, the global increase in disability-adjusted life years experienced by this age group was estimated to be 20.7% for depressive disorders [95% uncertainty interval: 17.4, 23.5] and 17.9% for anxiety disorders [95% uncertainty interval: 15.7, 20.3] (Vos et al., 2020). During the COVID-19 pandemic, analyses suggest there was a marked increase in prevalence of anxiety and depressive disorders, with younger age groups experiencing a greater increase than older groups (Racine et al., 2021; Santomauro et al., 2021).

In this context, research evaluating interventions for children and young people’s mental health has increased (Wellcome Trust, 2022). A 2021 review of trial registrations on *clinicaltrials.gov* revealed that the number of US-based child and adolescent mental health trials had increased at twice the rate of all mental health trials, and there had been a shift toward evaluations of preventive and behavioral (non-pharmacological) interventions (Wortzel et al., 2020). Such studies are typically conducted in community settings, with the majority occurring in schools (Stockings et al., 2016).

School-based prevention may be universal or targeted. Universal prevention addresses whole populations regardless of risk status. Targeted prevention includes populations at higher-than-average risk of mental health concerns (selective) and those with subclinical symptoms (indicated). Interventions may be embedded in the curriculum and standardized for classroom delivery by teachers or mental health professionals, be multi-component and multi-level “whole school” interventions, or provide support and counseling following psychotherapeutic models. To date, most school-based anxiety and depression prevention programs have been grounded in a cognitive-behavioral approach to mental health (Hetrick et al., 2016; Werner-Seidler et al., 2017). More recently, alternatives such as mindfulness (Kuyken et al., 2022), positive psychology (Garaigordobil et al., 2019), and physical activity combined with psychosocial approaches have also grown in popularity (Zapata-Lamana et al., 2024).

Evidence from high-quality systematic reviews of randomized controlled trials (RCTs) indicates a small beneficial effect of school-based preventive interventions for anxiety and depression, particularly for cognitive-behavioral interventions (Hetrick et al., 2016; Li et al., 2021; Stockings et al., 2016; Werner-Seidler et al., 2021; Zhang et al., 2023). However, concerns have been raised about the robustness of this evidence base. For example, the potential for intervention effects to be exaggerated due to (i) inadequately designed or conducted RCTs (Zhang et al., 2023) and/or (ii) the use of insufficiently stringent control groups (Merry et al., 2004).

Schools may be considered ideal for recruiting large numbers of young people for RCTs and to facilitate follow-up over extended periods of time (Moore et al., 2022). However, designing RCTs to minimize the potential for bias but maximize recruitment and retention of schools has been described as challenging (Plummer et al., 2014). “Trade-offs” may be necessary, even if these compromise internal validity (Loades et al., 2024). For example, allowing flexibility in intervention delivery across schools/classrooms rather than rigid adherence to a manual (Wheatley et al., 2020). Selecting a comparator that effectively controls for the natural course of symptoms, or for participant expectancy effects, is especially challenging in school-based studies. For example, “as usual” controls are often considered to be least disruptive to schools and are less resource intensive than “active” or “attention” controls. Conversely, schools may prefer allocation to an “active” intervention, perceiving an “as usual” control to be of less value or even unethical (Dawson et al., 2018). “Waiting list” controls, in which schools receive the experimental intervention after the end of the study, may be used to improve school recruitment and retention in RCTs. Waiting list may be considered less disruptive for the control schools while also offering an incentive for participation.

In a recent systematic review of school-based interventions, 75% of included studies used a no intervention, class/curriculum “as usual,” or a waiting list control group (Werner-Seidler et al., 2021). These controls are intended to mitigate against threats to internal validity. In comparison, “attention,” “non-specific,” and “active” comparators control for non-specific and specific intervention factors and may be considered more “stringent” (Freedland et al., 2011, 2019; Gold et al., 2017; Mohr et al., 2009). There is consistent evidence from the psychotherapeutic and behavioral change literature suggesting that control group choice can influence, or moderate, effect estimates, with an inverse association observed between the “stringency” of the control and the magnitude of the observed effect size (Black et al., 2020; Faltinsen et al., 2022; Freedland et al., 2011; Kraiss et al., 2023). Choice of control group has received little attention in meta-analyses of preventive mental health interventions and, where moderation effects have been explored, findings have been mixed (Moreno-Peral et al., 2017; Zhang et al., 2023).

In pairwise meta-analyses, it is common for usual class/curriculum, no intervention, and waiting list controls to be conflated into a single comparator for analysis (Caldwell et al., 2021). The Cochrane Handbook recommends meta-analysts carefully consider “…the different meanings of phrases such as ‘control’, ‘placebo’, ‘no intervention’ or ‘usual care’” in advance of synthesis (Higgins et al., 2019, p.44). However, Caldwell et al. (2021) noted that only 11/20 reviews of school-based interventions for prevention of anxiety and depression provided a study-level description of control group type. Among these 11 reviews, seven conducted meta-analyses with a conflated control group and only 2/7 provided a justification (Hetrick et al., 2016; Werner-Seidler et al., 2017).

Network meta-analysis (NMA) provides an ideal framework for exploring the moderating effect of control group type. NMA is an extension of pairwise meta-analysis that allows the simultaneous comparison of multiple, distinct interventions and comparators in a single analysis, thereby avoiding the need to conflate interventions or controls into single comparators solely for the purpose of analysis. Assuming a connected network of comparisons, effects can be estimated in the absence of head-to-head studies. This means coherently estimated effects for each control group type relative to every other can be obtained. As NMAs typically include a greater number of studies than standard meta-analyses and exploit both direct and indirect evidence in the estimation of effects, NMA may also allow for increased power to detect a moderating effect of control group type.

The aim of this paper is to examine the impact of control group type on the relative effectiveness of school-based interventions for the prevention of anxiety and depression, using NMA. We follow the approach taken by Furukawa et al. (2014) in their exploration of control group effects in psychotherapy for treatment of depression. Recognizing the increased number of “active” and “attention” controlled studies published over the last five years (Werner-Seidler et al., 2021), this nested methodological study involved a rapid update of our original review (Caldwell et al., 2019) to include additional studies directly relevant to estimating control group moderator effects.

## Method

### Rapid Update Search Strategy and Selection Criteria

The final searches on which the rapid update is based were run on 30/06/2023 (see Appendix). The search strategy for our original systematic review was developed by an information specialist to identify RCTs and was run in April 2018 using MEDLINE, Embase, PsycINFO, and Cochrane Central Register of Controlled Trials (CENTRAL). A modified, “two-stage” search strategy was followed for this rapid update (Nussbaumer-Streit et al., 2023). Firstly, we ran searches to identify systematic reviews published from 01/01/2016 onward and so omitted CENTRAL from the update searches. Eligible reviews were used to identify relevant primary studies for inclusion in this rapid update. If we did not identify recent systematic reviews, we planned supplementary, targeted searches to identify randomized controlled trials. The most recent reviews included studies published up to July 2021. As such, we also ran targeted keyword searches to identify primary studies in MEDLINE, restricting to RCTs published from 01/01/2020 onward.

Full details of inclusion and exclusion criteria are published in the original systematic review (Caldwell et al., 2021). Eligible primary studies were individually and cluster-randomized controlled trials. Studies described by trialists as “quasi-experimental” were considered eligible if appropriate randomization and allocation methods were reported. For example, cluster randomized studies may be described as quasi-experimental in some fields (Reeves et al., 2017). The population of interest was young people aged 5–18 years attending pre-, primary-, or secondary-educational settings. Using the Institute of Medicine’s definition of primary prevention, eligible populations were universal or targeted (selective or indicated), and eligible interventions were psychological, psychosocial, educational, or physical. Interventions delivered on school grounds to individuals or groups were eligible for inclusion. Interventions that took place elsewhere (e.g., in a clinic, community center, or university department) were not eligible, even if young people were recruited via the educational setting. Studies with an explicit aim of preventing anxiety and/or depression were eligible. Studies focused on general mental health promotion or mental health literacy were not eligible unless the explicit aim was the prevention of anxiety or depression symptoms. Trial registrations and protocols were consulted, and authors were contacted for further information, if required. Mobile phone and digital interventions were excluded if they were delivered off-school grounds. Studies in which schools were utilized for participant recruitment but where the intervention was not school-based were excluded. Primary care and other community settings were also excluded.

Full details of the review process followed for studies identified in the original systematic review are provided in Caldwell et al. (2019) and Caldwell et al. (2021). For the rapid update, study selection was independently assessed by two reviewers and disagreement resolved by a third, if necessary. Data extraction, intervention classification, and risk of bias assessment were completed by one reviewer and verified by a second, by comparing extracted data to the original paper. Discrepancies were discussed with a third reviewer. For team contributions, please see Appendix. Study authors were contacted for additional details as necessary. The rapid update review was prospectively registered with PROSPERO on 31/07/2023 (CRD42023448656) and was updated on 12/02/2024 with a statistical analysis plan. The present paper reports a nested methodological study exploring control group moderation. However, the updated effectiveness NMA results are also briefly reported in accordance with the PRISMA extension statement for NMA (Hutton et al., 2015).

### Methods for Network Meta-Analysis

The intervention and control group classifications were developed for our previous review and were informed by existing classification schemes (Davies et al., 2018; Furukawa et al., 2014; Hetrick et al., 2016). Interventions were categorized as behavioral, bias modification, biofeedback, cognitive-behavioral, exercise, interpersonal, third-wave, occupational, and mindfulness/relaxation-based interventions. Positive psychology was identified as a new intervention class in the update. Controls and comparators were categorized as attention control, no intervention, supportive, education, usual curriculum, and waiting list. The classification scheme is reported in the Appendix.

The outcomes of interest were anxiety and/or depression symptoms, as assessed by a standardized, validated self‐report measurement scale. Where studies used multiple scales, a pre-specified hierarchy was applied to select the most appropriate scale for the NMA (Appendix). Studies contributing to either the depression or anxiety analyses could be studies that aimed to prevent (i) depression, (ii) anxiety, or (iii) anxiety and depression (Dalgleish et al., 2020). If a study reported a composite outcome (e.g., total combined anxiety and depression, or “internalizing” symptoms) but otherwise met inclusion criteria, it was eligible for the review but not included in the NMA. The timepoint was immediately post-intervention and was based on study completers (available cases). Data were extracted on the number of participants in each arm at baseline and post-intervention, and baseline and post-intervention means with standard deviations. If mean change from baseline was reported, this was also extracted. Data were summarized as arm-level mean change from baseline and standard errors in advance of synthesis, assuming a correlation of 0.6 between pre and post measures. We used the standardized mean difference (Hedge’s *g*) to summarize intervention effects, with 95% credible intervals. Guidance from the Cochrane Handbook was used to estimate an approximate sample size for cluster RCTs, where relevant (Higgins et al., 2019).

The key assumption underpinning NMA is exchangeability of intervention effects (Ades et al., 2024). To assess the plausibility of this assumption, a priori checks of “transitivity” were conducted following guidance in the Cochrane Handbook (Chaimani et al., 2019). Study level and participant characteristics were tabulated and visually examined to check that the “distribution” of potential effect modifiers was balanced across comparisons. We also considered the principle of “joint randomizability,” i.e., whether participants could, theoretically, have been randomized to any of the studies or interventions (Salanti, 2012).

Random effects NMA were conducted in WinBUGS by population (universal or targeted) and educational setting. Educational setting was assessed by reviewers as “primary” (5–11 years) or “secondary” (12– ≤ 18 years). Allocation to a “setting” was based on the age range or average participant age at baseline and was for analytical purposes only. NMA model code was adapted from Dias et al., (2013a, 2013b). Heterogeneity was assessed using the posterior median between-study standard deviation (τ) and 95% credible intervals (CrI). The posterior mean residual deviance and the deviance information criterion were used as global measures to assess the presence of inconsistency, by comparing the goodness-of-fit of an unrelated mean effect and a consistency model (Dias et al., 2013a, 2013b). Sensitivity analyses excluding studies at high and unclear risk of bias were conducted. Subgroup analyses to explore whether intervention effect varied by facilitator or intervention format were conducted. Full details on model code, subgroup analyses, model fit, and model selection criteria are reported in the Appendix.

A standardized mean difference (SMD) of 0.20 was considered meaningful (Higgins et al., 2019). Interpretation of statistical findings was based on the magnitude and direction of the SMD (95% CrI). We assessed the strength of statistical evidence on a graduated scale from weaker to stronger statistical evidence of an intervention effect (Sterne & Davey Smith, 2001). Certainty of the evidence was evaluated using CINeMA, for the full NMA only. SMD 0.20 informed the minimally important difference (MID) in the CINeMA assessments (Nikolakopoulou et al., 2020).

### NMA to Explore Control Group Moderation Effects

**Fig. 1:**
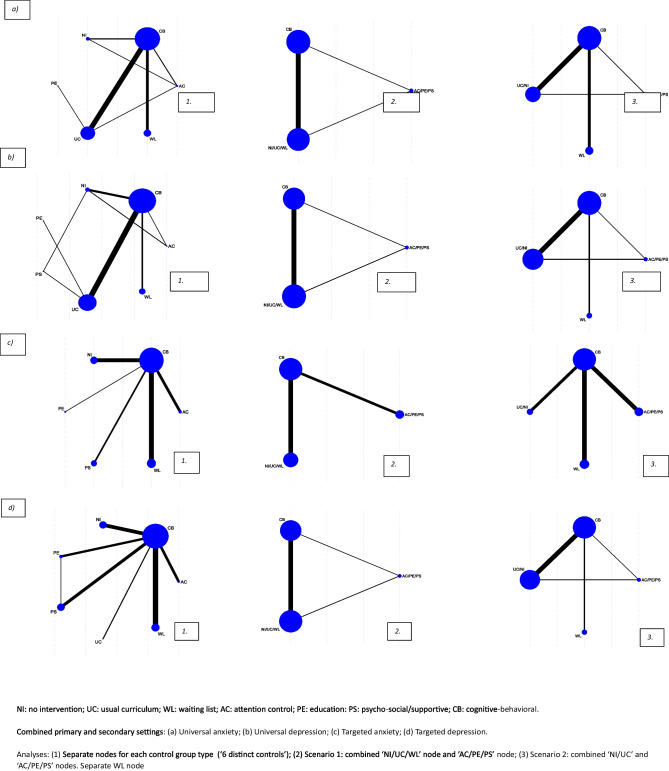
Network plots by population and outcome for cognitive behavioral vs control analyses

Subgroup and moderation analyses are typically subject to low statistical power, particularly in the presence of between study heterogeneity in intervention effects. Informed by previous work (Caldwell et al., 2021), we anticipated moderate statistical heterogeneity in the full NMA and that most “active” interventions would be cognitive-behavioral (Caldwell et al., 2021; Werner-Seidler et al., 2021; Zhang et al., 2023). Therefore, to minimize heterogeneity, we restricted the control group analyses to studies including a cognitive-behavioral intervention compared to attention, no intervention, supportive, education, usual curriculum, and waiting list controls only. We also combined primary and secondary educational settings for the control group NMA, as this maximized the number of eligible studies and can improve network density (Furukawa et al., 2014). This decision was taken to improve statistical power. However, before proceeding with the control group NMA, meta-regression analyses were conducted to check for evidence of an interaction between effect estimates and educational setting. Combining across settings also allowed comparison of our findings with those from other comprehensive systematic reviews, as this is a common approach in pairwise meta-analyses (e.g., Hetrick et al., 2016; Stockings et al., 2016; Werner-Seidler et al., 2021) (Fig. 1).

Multi-arm studies including two or more control arms vs cognitive-behavioral intervention were included (e.g., usual curriculum vs attention control vs cognitive-behavioral). Where multi-arm studies included two “active” interventions vs a control, only the cognitive-behavioral and control arm data were retained (e.g., for no intervention vs cognitive-behavioral vs mindfulness, we retained only the no intervention and cognitive-behavioral arms). Studies comparing a cognitive-behavioral intervention vs an alternative “active” intervention (e.g., cognitive-behavioral vs interpersonal) were not included. Results from this analysis are reported as “Main control group analysis: NMA of cognitive-behavioral intervention relative to six distinct control groups.” The posterior median between-study standard deviation, model fit, and selection statistics were assessed. Where network connectedness could be maintained, we assessed the robustness of our findings to exclusion of small studies (< 50 participants) and exclusion of studies at high and unclear risk of bias. Subgroup analyses by study design were conducted (cluster or individual randomization).

### Post Hoc Scenario Analyses to Approximate Standard Pairwise Meta-Analytic Approaches

As noted in the introduction, results from previous analyses of control group moderation effects have been mixed. Ten out of 44 systematic reviews identified in Caldwell et al. (2021) (*n* = 20) or for this rapid update (*n* = 24, after de-duplication) reported quantitative results by control group (see Appendix Table 1). To compare our control group findings with those from other reviews, we approximated their meta-analytic approaches in two post hoc scenario analyses.

**Table 1:** Results from rapid update NMA: depression outcome

Intervention/ comparator	Universal secondary		CINeM A	Targeted secondary		CINeMA	Universal primary		CINeMA	Targeted primary		CINeMA
	**SMD**	**(95%CrI)**		**SMD**	**(95%CrI)**		**SMD**	**(95%CrI)**		**SMD**	**(95%CrI)**	
Usual curriculum	*Reference*		-	0.00	(−0.75, 0.77)	Low	*Reference*		-	NA		-
Waiting list	0.01	(−0.13, 0.15)	Low	0.15	(−0.32, 0.60)	Low	−0.11	(−0.75, 0.48)	Low	*Reference*		*-*
No intervention	0.06	(−0.09, 0.23)	Low	*Reference*		-	0.02	(−0.54, 0.57)	Low	NA		-
Attention control	0.13	(−0.12, 0.38)	Low	−0.85	(−1.85, 0.14)	Low	−0.16	(−0.87, 0.53)	Low	−0.72	(−3.54, 2.07)	Low
Cognitive-behavioral	−0.01	(−0.10, 0.07)	Very low	−0.25	(−0.60, 0.08)	Low	−0.16	(−0.42, 0.10)	Low	−0.48	(−2.48, 1.50)	Low
Third wave	−0.03	(−0.17, 0.11)	Low	−3.76	(−4.98, −2.53)	Low	NA		-	NA		-
CB + IP	−0.16	(−0.40, 0.08)	Low	NA		-	NA		-	NA		-
IP	−0.01	(−0.30, 0.27)	Low	−0.46	(−1.25, 0.32)	Low	NA		-	NA		-
Education	−0.13	(−0.44, 0.17)	Low	0.04	(−0.49, 0.55)	Low	NA		-	NA		-
Positive Psychology	0.07	(−0.24, 0.37)	Low	−1.15	(−2.34, 0.01)	Low	−0.25	(−1.22, 0.71)	Low	NA		-
Behavioral	−0.02	(−0.36, 0.33)	Low	NA		-	−0.03	(−0.68, 0.61)	Low	NA		-
Exercise	−0.13	(−0.45, 0.19)	Low	NA		-	−0.20	(−1.13, 0.69)	Low	NA		-
Mindfulness	−0.04	(−0.20, 0.12)	Low	NA		-	0.19	(−0.51, 0.89)	Low	NA		-
Bias modification	NA		-	−0.94	(−2.25, 0.36)	Low	0.16	(−0.75, 1.07)	Low	NA		-
Supportive	0.08	(−0.11, 0.26)	Very low	0.22	(−0.36, 0.80)	Low	NA		-	−0.29	(−3.73, 3.15)	Low
Occupational therapy	NA		-	NA		-	NA		-	−0.10	(−2.91, 2.72)	Moderate
*N studies*	48 0.12 (0.08, 0.17)			30 0.39 (0.27, 0.56)			16 0.31 (0.18, 0.55)			6 0.60 (0.07, 3.79)		
*τ* (95% CrIs)												

Table 1 report the standardized mean difference (SMD) and 95% Credible Intervals (CrIs) from random effects network meta-analyses, for each intervention relative to a “reference” intervention. Due to network structure, the reference intervention was not the same across the four population-setting analyses. *τ* is the between study heterogeneity in treatment effects (standard deviation). If *τ* = 0, or is close to zero, it indicates minimal between study heterogeneity

*CINeMA* confidence in network meta-analysis, *NA* intervention not present in network, *CB* cognitive behavioral, *IP* interpersonal

In Scenario Analysis 1 (Fig. 1), our six distinct control groupings were collapsed to form two comparators (i) a combined no intervention, usual curriculum, and waiting list group, and (ii) a combined attention control, supportive, and education control. This approach approximates subgroup analyses in Lawrence et al. (2017), Stockings et al. (2016), Teubert (2011), and Ssegonja et al. (2019). However, it also approximates approaches taken to the pairwise meta-analysis in all ten reviews identified.

Scenario Analysis 2 (Fig. 1) approximated a second approach as reported by Werner-Seidler et al. (2017), Hugh-Jones et al. (2021), Werner-Seidler et al. (2021), and Moreno-Peral et al. (2017). In Scenario Analysis 2, our six distinct control group types were collapsed into three groups: (i) a combined no-intervention and usual curriculum control, (ii) a combined attention control, supportive, and education control, and (iii) a separate waiting list control grouping. In the results below, we refer to these analyses as “Scenario Analysis 1: to approximate Stockings” and “Scenario Analysis 2: to approximate Werner-Seidler” (see appendix for WinBUGS code). The Stockings and Werner-Seidler reviews included the greatest number of studies, which are highly cited, and their inclusion criteria most closely resemble those of the present rapid update.

## Results

### Rapid Review Update

**Fig. 2:**
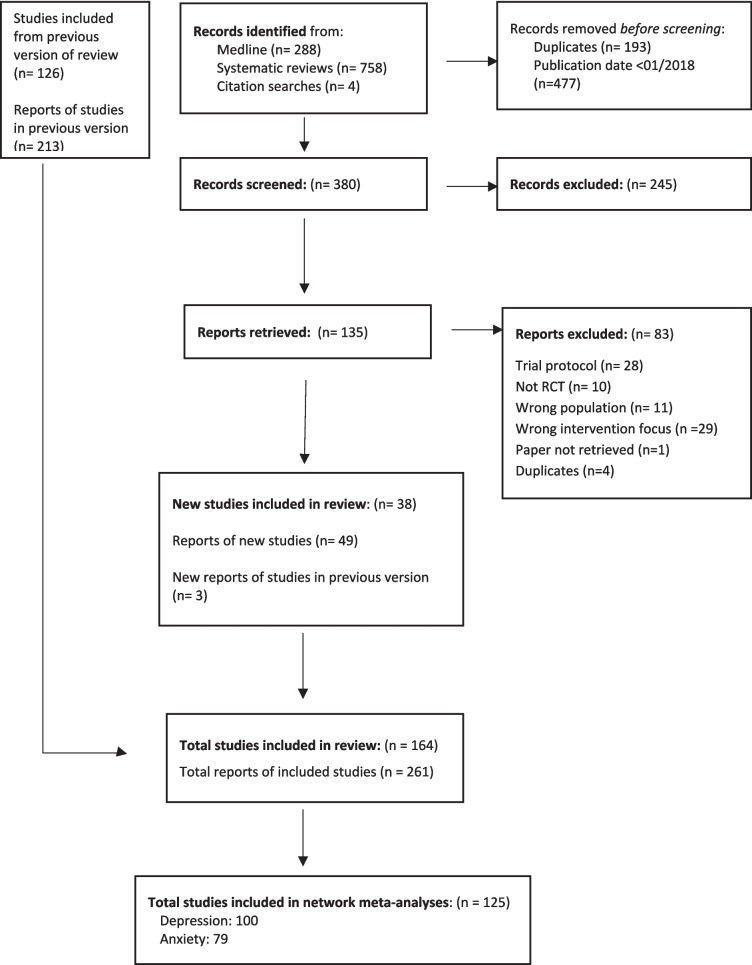
PRISMA flow diagram. Flowchart indicating the number of studies at the searching, screening, and analysis stages of the review process

The review included 164 studies, of which 38 were identified from the update searches (Fig. 2) (23 from keyword searches and 15 from systematic reviews). Study characteristics and risk of bias assessments are reported in the Appendix.

Studies were published between 1982 and 2022. Sample size ranged from 13 to 8376 participants (median: 209 participants). Seventy-five studies were individually randomized and 89 were cluster randomized. One-hundred and forty-four studies were conducted in high-income countries and 20 in low or middle-income countries. One hundred studies were classified as universal, and 64 as targeted. Forty-nine studies were implemented in primary and 111 in secondary educational settings. Four were conducted in both settings.

Self-reported anxiety was assessed in 97 studies and depression in 122 studies (70 assessed both). One-hundred and nine studies included a cognitive-behavioral-based intervention, 17 studies included a mindfulness or relaxation-based intervention, four included interventions based on interpersonal therapy, seven included a “third wave” intervention, nine included a behavioral intervention, four utilized biofeedback methods, five studies included an exercise intervention, three used bias modification approaches, four combined cognitive-behavioral and interpersonal approaches, and seven evaluated a positive psychology-based intervention. One study used an occupational therapy-based intervention.

Forty-seven studies included an attention (AC), or a “non-specific” supportive (PS) or education (PE) control arm (total number of study arms: AC *n* = 26, PE *n* = 7, PS *n* = 15). Forty-nine studies included a waiting list (WL), 54 included a usual curriculum (UC), and 20 included a no intervention (NI) arm. Four studies compared ≥ 2 experimental interventions. Thirty-eight studies were multi-arm, of which 29 compared two active interventions against a control. Eighty-five percent of universal prevention studies included either WL (*n* = 27), UC (*n* = 49), or a NI (*n* = 9) control arm. For targeted studies, this was 59% (WL *n* = 22, UC *n* = 5, NI *n* = 11). For studies with total sample sizes > 101, 79% included a WL, UC, or NI control arm. For studies with sample sizes < 100, this was 61%. Eighty-six percent of cluster randomized and 60% of individually randomized studies included a WL, UC, or NI control.

Sixty-three percent of the newly identified studies included a no intervention, usual curriculum, or waiting list control arm, compared to 77% of studies included in the 2019 review. The proportion of studies identified in the 2019 review that included an attention, supportive, or education arm was 25% and in the update was 39%. The proportion of studies including waiting list also appears to have increased, albeit very slightly. For example, 26% of studies included a waiting list arm in 2006–2010, but in 2011–2015, this was 29% and in 2016–2020 was 31% (full results reported in Appendix).

### Updated Effectiveness Results from Full NMA

Analyses were conducted by educational setting and population, and network plots for each analysis are reported in the Appendix. Transitivity was assessed across studies contributing to each analysis. Potential effect modifiers were considered balanced across comparisons. Model fit and selection statistics supported an assumption of consistency (Appendix).

Seventy-nine studies contributed to the anxiety NMA, of which 35 were focused on anxiety prevention, 12 focused on depression, and 32 on both prevention of anxiety and depression. One hundred studies contributed to the depression NMA, of which 54 were focused on depression prevention, 10 focused on anxiety, and 36 on prevention of both anxiety and depression. Updated effectiveness findings are reported in Table 1 by population and educational setting and are similar to those observed in our 2019 review (Appendix). Between study posterior median standard deviations (τ) indicate low to moderate between study heterogeneity. Across all eight population-setting-outcome analyses, confidence in summary effect estimates was mostly judged to be low (Table 1 and Table 2. Overall, SMDs were indicative of very small to small beneficial effects of intervention relative to a reference. However, for most comparisons, the range of effects contained in the 95% credible intervals (CrI) was consistent with both an important intervention effect and with no intervention effect. Findings were robust to sensitivity analyses and there was no evidence of subgroup effects by facilitator or intervention format. Full sensitivity and subgroup results are reported in the Appendix.

**Table 2:** Results from rapid update NMA: anxiety outcome

Intervention/ comparator	Universal secondary		CINeM A	Targeted secondary			CINeMA	Universal primary			CINeMA	Targeted primary		CINeMA
	**SMD**	**(95%CrI)**		**SMD**		**(95%CrI)**		**SMD**	**(95%CrI)**			**SMD**	**(95%CrI)**	
Usual curriculum	*Reference*		-			NA	-	*Reference*			-	NA		-
Waiting list	0.02	(−0.10, 0.14)	Low	0.31		(0.13, 0.50)	Moderate	−0.02		(−0.24, 0.19)	Low	*Reference*		*-*
No intervention	0.01	(−0.19, 0.20)	Low	*Reference*			-	0.19		(−0.34, 0.72)	Low	NA		-
Attention control	0.03	(−0.15, 0.21)	Low	−0.10		(−0.39, 0.21)	Low	−0.21		(−0.56, 0.14)	Low	−0.38	(−1.10, 0.32)	Low
Cognitive-behavioral	−0.04	(−0.14, 0.04)	Low	0.03		(−0.10, 0.16)	Low	−0.12		(−0.27, 0.01)	Low	−0.38	(−0.84, 0.06)	Low
Mindfulness	−0.15	(−0.41, 0.10)	Low	0.03		(−0.40, 0.47)	Low	−0.11		(−0.80, 0.57)	Low	NA		-
Positive Psychology	−0.14	(−0.41, 0.12)	Low	−0.59		(−0.96, −0.22)	Moderate	NA			-	NA		-
Third wave	0.03	(−0.08, 0.15)	Low	NA			-	NA			-	NA		-
Education	NA		-	0.13		(−0.19, 0.45)	Low	−0.02		(−0.35, 0.31)	Low	NA		-
Bias modification	NA		-	−0.17		(−0.44, 0.10)	Moderate	−0.10		(−0.45, 0.22)	Low	NA		-
Exercise	NA		-	NA			-	NA			-	NA		-
Biofeedback	NA		-	−0.17	(−0.54, 0.19)		Low	NA			-	−0.49	(−1.38, 0.39)	Low
Supportive	NA		-	0.82	(0.41, 1.22)		Moderate	NA			-	0.10	(−0.99, 1.19)	Low
Occupational therapy	NA		-	NA			-	NA			-	0.11	(−0.91, 1.13)	Moderate
*N studies*	29 0.06 (0.00, 0.14)			20 0.05 (0.00, 0.18)				18 0.12 (0.01, 0.27)				12 0.42 (0.21, 0.89)		
*τ* (95% CrIs)														

Table 2 report the standardized mean difference (SMD) and 95% Credible Intervals (CrIs) from random effects network meta-analyses, for each intervention relative to a “reference” intervention. Due to network structure, the reference intervention was not the same across the four population-setting analyses. *τ* is the between study heterogeneity in treatment effects (standard deviation). If *τ* = 0, or is close to zero, it indicates minimal between study heterogeneity

*CINeMA* confidence in network meta-analysis, *NA* intervention not present in network, *CB* cognitive behavioral, *IP* interpersonal

In universal secondary and primary educational settings, there may be some evidence of a very small effect in favor of cognitive-behavioral intervention relative to a usual curriculum reference for anxiety symptoms (secondary: SMD − 0.04 [95% CrI − 0.14, 0.04]; primary: SMD − 0.12 [95% CrI − 0.27, 0.01]). For targeted primary settings, there was weak statistical evidence of an effect of cognitive-behavioral intervention over a waiting list reference (SMD − 0.38 [95% CrI − 0.84, 0.07]). In targeted secondary settings, there was strong evidence of a large effect for third wave-based interventions relative to no intervention for depression symptoms (SMD − 3.76 [95% CrI − 4.98, − 2.53]). A moderate effect was also observed for positive psychology-based interventions relative to no intervention for anxiety symptoms (SMD − 0.59 [95% CrI − 0.96, − 0.22]).

### NMA to Explore Control Group Moderation Effects

Figure 1 presents network plots by population and outcome for the main control group analysis (six distinct controls) and Scenario Analysis 1 and 2. Studies (*n* = 90) contributing to the control group analyses are a subset of those from the full NMA. For universal populations, 43 studies were included in the depression and 37 in the anxiety control group analyses. For targeted populations, 28 studies contributed to the depression and 24 to the anxiety analyses. Between study posterior median standard deviations indicated low to moderate heterogeneity in all analyses. Meta-regression analyses provided no evidence of an interaction between educational setting and effect estimates. Model fit and selection statistics supported an assumption of consistency, and that combining studies across primary and secondary educational settings was reasonable. Model fit statistics and results for the meta-regression are reported, in full, in the Appendix.

#### Main Control Group Analysis: NMA of Cognitive Behavioral Relative to Six Distinct Control Groups

The main control group NMA (“six distinct controls”) considered the effectiveness of cognitive-behavioral intervention relative to each of the six distinct control group types, and of each control group relative to every other (Fig. 1). SMDs and 95% CrI are reported in Table 3 (a and b) separately by population (universal or targeted) and by outcome (anxiety or depression). For consistency, the results are reported below such that SMDs less than 0 favor the intervention listed first (the “experimental” intervention). Intervention rankings (95% CrI) were requested during peer review and, for completeness, are reported in the Appendix.

**Table 3:**
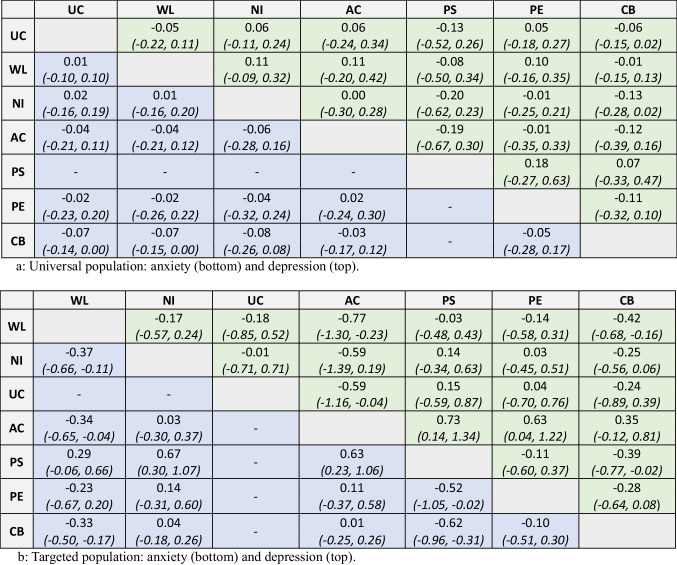
Results from main control group moderation NMA (six distinct control groups)

Standardized mean difference (SMD) and 95% credible intervals (CrIs) between six control groups and CB (cognitive behavioral) intervention interventions. *UC* usual curriculum, *WL* waiting list, *NI* no intervention, *AC* attention control, *PS* supportive, *PE* education. Analysis conducted combining primary and secondary educational settings

Anxiety: (bottom left cells) intervention in each row is ‘experimental’ and in each column is the ‘control’: e.g., the WL vs AC denotes the effect of AC (‘experimental’) over WL (‘control’). SMD < 0 favors row comparator

Depression: (top right cells) the intervention in each column is ‘experimental’ and in each row is the ‘control’ SMD < 0 favors column comparator

Studies contributing to each analysis: universal depression *N* = 43; universal anxiety *N* = 36. Targeted depression *N* = 28; targeted anxiety total *N* = 24

In universal populations, for both depression and anxiety outcomes, there was no strong evidence of intervention effect for cognitive-behavioral intervention relative to any of the control group types. However, for anxiety symptoms, there may be weak statistical evidence of a very small effect favoring cognitive-behavioral intervention relative to usual curriculum (SMD − 0.07 [95% CrI − 0.14, 0.00]) and waiting list (SMD − 0.07 [95% CrI − 0.15, 0.00]).

For targeted populations, there was evidence of a moderate effect in favor of cognitive-behavioral intervention relative to waiting list for depression (SMD − 0.42 [95% CrI − 0.68, − 0.16]) and anxiety (SMD − 0.33 [95% CrI − 0.50, − 0.17]). There was also evidence in favor of cognitive-behavioral intervention relative to a supportive control (depression: − 0.39 [95% CrI − 0.77, − 0.02]; anxiety: − 0.62, [95% CrI − 0.96, − 0.31]). However, there was no evidence of an effect for cognitive-behavioral intervention relative to no intervention for depression or anxiety (depression: SMD − 0.25 [95% CrI − 0.56, 0.06]; anxiety: SMD 0.04 [95% CrI − 0.18, 0.26]). There was also no evidence of an effect for cognitive-behavioral intervention relative to attention control (depression: SMD 0.35 [95% CrI − 0.12, 0.81]; anxiety: SMD 0.01 [95% CrI − 0.25, 0.26]).

In the targeted anxiety analysis, there was also statistical evidence for a moderate effect of no intervention relative to waiting list (SMD − 0.37 [95% CrI − 0.11, − 0.66]) and of attention control relative to waiting list (SMD − 0.34 [95% CrI − 0.65, − 0.04]). For targeted depression, there was evidence of a moderate effect of attention control relative to waiting list (SMD − 0.77 [95% CrI − 1.30, − 0.73]) and for no intervention over attention control (SMD − 0.59 [95% CrI − 1.16, − 0.04]). There was no evidence for an effect of no intervention relative to waiting list (SMD 0.17 [95% CrI − 0.24, 0.57]).

#### Sensitivity and Subgroup Analyses for Main Control Group NMA (Six Distinct Control Groups)

Full results for sensitivity and subgroup results are reported in the Appendix. Across all population and outcome combinations, findings were unchanged after removing studies with a sample size of < 50. After restricting the control group analyses to studies at low risk of bias, results were also largely unchanged. However, in the universal depression analysis, the direction of effect for attention control relative to usual curriculum was reversed (SMD 0.23 [95% CrI 0.04, 0.44]). In targeted depression, a strong effect of attention control relative to usual curriculum was observed in studies at low risk of bias (SMD − 1.59 [95% CrI − 2.99, − 0.18]). Findings were also largely unchanged for subgroup analyses by study design. However, for universal depression, the effect of cognitive-behavioral intervention relative to usual curriculum was SMD − 0.43 ([95% CrI − 0.77, − 0.10], *n* = 12) for individually randomized studies and was SMD − 0.04 ([95% CrI: − 0.11, 0.04], *n* = 31) for cluster randomized studies. For the universal anxiety analysis, the effect of cognitive-behavioral relative to waiting list for individually randomized studies was SMD − 0.36 ([95% CrI − 0.70, − 0.08], *n* = 7) and for cluster randomized studies was SMD − 0.04 ([95% CrI − 0.13, 0.05], *n* = 30).

#### ***Control Group NMA: Scenario Analysis 1 (to Approximate ***Stockings et al., 2016***)***

Scenario Analysis 1 combined the six controls into two groupings for comparison with cognitive-behavioral intervention: (i) no intervention (NI), usual curriculum (UC), and waiting list (WL) were combined, and (ii) attention (AC), supportive (PS), and education (PE) controls were combined. In universal populations, there was evidence of an effect of cognitive-behavioral intervention relative to the lumped NI/UC/WL control for anxiety symptoms (SMD − 0.06 [95% CrI − 0.11, − 0.02]) but not relative to the lumped AC/PS/PE control group (SMD − 0.03 [95% CrI − 0.15, 0.08]). For depressive symptoms, there was evidence of an effect of cognitive-behavioral intervention relative to NI/UC/WL (SMD − 0.06 [95% CrI − 0.13, − 0.001]) but not AC/PE/PS (SMD − 0.06 [95% CrI − 0.22, 0.08]).

For targeted populations, there was evidence of an effect of cognitive-behavioral intervention relative to NI/UC/WL (Depression: SMD − 0.34 [95% CrI − 0.54, − 0.15]; anxiety: SMD − 0.21 [95% CrI − 0.39, − 0.04]) but not to AC/PE/PS (depression: SMD − 0.16 [95% CrI − 0.41, 0.08]; anxiety: SMD − 0.22 [95% CrI: − 0.46, 0.01]).

#### ***Control Group NMA: Scenario Analysis 2 (to Approximate ***Werner-Seidler et al., 2021***)***

Scenario Analysis 2 combined the six controls into three groupings for comparison with cognitive-behavioral intervention: (i) no intervention (NI) and usual curriculum (UC) were combined into a single comparator, (ii) attention (AC), supportive (PS), and education (PE) were combined into a single comparator, and (*iii*) a separate waiting list control.

In universal populations, there was some evidence of an effect of cognitive-behavioral intervention relative to the combined “NI/UC” control group for anxiety symptoms (SMD − 0.06 [95% CrI − 0.13, − 0.01]) and weak evidence of an effect of cognitive-behavioral intervention relative to WL (SMD − 0.07 [95% CrI − 0.14, − 0.002]). However, there was no evidence of an effect of NI/UC relative to WL (SMD 0.01 [95% CrI − 0.09, 0.09]) or NI/UC relative to AC (SMD − 0.03 [95% CrI − 0.16, 0.09]). For depression symptoms, there was some evidence of effect of cognitive-behavioral intervention relative to NI/UC (SMD − 0.08 [95% CrI − 0.16, − 0.01]). However, there was no evidence of an effect of NI/UC relative to WL (SMD − 0.07 [95% CrI − 0.22, 0.08]) or NI/UC relative to AC (SMD − 0.00 [95% CrI − 0.16, 0.15]).

In targeted populations, there was no evidence of an effect of cognitive-behavioral intervention relative to NI/UC for anxiety (SMD 0.04 [95% CrI − 0.22, 0.31]). However, relative to WL and a lumped AC/PE/PS control, there is evidence of a moderate effect in favor of cognitive-behavioral intervention (WL: SMD − 0.34 [95% CrI − 0.55, − 0.15]; AC/PE/PS: SMD − 0.22 [95% CrI: − 0.43, − 0.01]). For depression symptoms, there was strong evidence of a moderate effect of cognitive-behavioral intervention relative to WL (SMD − 0.42 [95% CrI − 0.69, − 0.15]). There may be weak evidence of a small effect of cognitive-behavioral intervention relative to a “lumped” NI/UC control (SMD − 0.25 [95% CrI − 0.55, 0.03]). For anxiety symptoms, there was strong evidence of moderate effect in favor of NI/UC relative to WL (SMD 0.38 [95% CrI 0.06, 0.73]). However, for depression symptoms, there was no evidence of an effect of NI/UC relative to WL (SMD 0.17 [95% CrI − 0.24, 0.56]) or to a lumped AC/PE/PS (SMD − 0.09 [95% CrI − 0.48, 0.29]).

## Discussion

The purpose of this paper was to explore if control group type moderated intervention effect in a large network meta-analysis of school-based interventions for the prevention of anxiety and depression. Ninety studies were included in the control group analyses, of which 13 were identified in the update searches and 77 from our original systematic review (Caldwell et al., 2021).

There was no evidence to suggest intervention effect was moderated by control group in the universal population analyses. However, for targeted populations, we found that the SMD of cognitive-behavioral intervention was larger relative to waiting list than to usual curriculum, no intervention, and attention controls for both anxiety and depression. For targeted anxiety, we also observed strong evidence of an effect of no intervention compared to waiting list, in favor of no intervention (SMD − 0.37 [95% CrI − 0.11, − 0.66]). That is, there was conventionally “significant” evidence of a beneficial effect of no intervention and attention control compared to a waiting list control, with clear implications for use of waiting list controls in trial design and interpretation. These findings mirror those of Furukawa et al. (2014) who described waiting list as a “nocebo” relative to cognitive-behavioral therapy for the treatment of depression.

For both targeted anxiety and depression, there was evidence of an effect for “non-specific” supportive control relative to attention control. In this paper, an attention control classification was applied to de novo, non-psychological, interventions implemented for the purposes of the RCTs only. Examples include study-skills sessions, arts and crafts sessions, and watching a documentary. “Non-specific” controls were classified as supportive if the intervention was not usually available to the participants in their setting but did not contain cognitive or behavioral elements associated with the “active” intervention. Examples include guidance, supportive humanistic, or supportive expressive sessions. Therefore, our findings that supportive controls may be “more effective” than non-psychological attention controls may not be surprising. However, we also observed some evidence of an effect of attention control compared to waiting list, in favor of attention control (anxiety: SMD − 0.34 [95% CrI − 0.65, − 0.04]; depression: SMD − 0.77 [95% CrI − 1.30, − 0.23]). Our findings were unchanged when very small studies (< 50 participants) were excluded and there was no evidence that control group effects in targeted populations differed by study design.

Although not the primary focus, this paper also reports updated effectiveness results from the full NMA. We included 164 studies in the review (38 from the update) and 125 studies were included in the full NMA (26 from the update) (Fig. 2). We found insufficient evidence of an effect for universal school-based interventions relative to a reference control immediately post-intervention. There was evidence of an effect of third wave and positive psychology interventions in targeted populations, although these estimates were directly informed by one (third wave, *n* = 1 (Livheim et al., 2015) and two studies (positive psychology, *n* = 2 (Osborn et al., 2021; Osborn et al., 2020)) and were connected to the network via a “spur.” That is, they were not well-connected to the network and further studies are needed before conclusions can be drawn about intervention effectiveness.

### Potential Limitations and Comparison with Previous Reviews

A key strength of NMA is that, by allowing for a greater number of studies and comparisons to be included in an analysis, the power to detect a moderating effect of control group type may be improved. However, the number of studies in each control group analysis ranged from 24 to 43 and the mean number of study arms per direct comparison was six (range 1–15). Coupled with the moderate to substantial heterogeneity observed, it is likely that our analyses had insufficient power to detect differential control group effects. Additionally, we made multiple comparisons without correction for multiple testing and cannot rule out the possibility of type I error explaining the control group findings. Further research would likely require the use of individual participant data to explore this robustly in NMA.

Although we followed guidance from the Cochrane Rapid Review methods group (Nussbaumer-Streit et al., 2023), the “rapid” nature of the update review may be a limitation. For example, in using systematic reviews as the main source for identifying new RCTs, we may not have identified all relevant studies published between April 2018 and June 2023. Seventy-nine percent of the studies contributing to the full NMA were identified from our previous full systematic review. As such, our findings are similar to those from our previous review (see Appendix) and are consistent with results from large-scale RCTs (Aune & Stiles, 2009; Calear et al., 2009, 2016; Kindt et al., 2014; Sheffield et al., 2006; Spence et al., 2003; Stallard et al., 2013, 2014; Tak et al., 2016). However, systematic reviews published since 2019 continue to report beneficial effects of school-based prevention for depression and anxiety. In a pairwise meta-analysis of 29 studies, Zhang et al. (2023) reported an overall “effect size” of 0.24 (*p* = 0.002) for a combined depression and anxiety outcome. Werner-Seidler et al. (2021) reported separate estimates for anxiety (*g* = 0.18 [95% CI 0.12, 0.26], *n* = 72), and depression (*g* = 0.21 [95% CI 0.17, 0.24], *n* = 101) at post-intervention.

Both Zhang’s and Werner-Seidler’s summary effect estimates are from meta-analyses that combined across targeted and universal populations, and primary and secondary educational settings. Both reviews also used a conflated control group, “lumping” no intervention, usual curriculum, waiting list, and attention controls to form a single comparator for use in meta-analyses. Such differences in meta-analytic methods and data preparation for analysis may account for the differing findings between their reviews and the current paper. For example, these differences may impact on the degree of between study heterogeneity, precision of effect estimates, and statistical power. The two post hoc scenario analyses provide an opportunity to explore the impact of these methodological differences on findings. Scenario Analysis 1 approximated a standard approach to pairwise meta-analysis, in which attention, education, and supportive controls were collapsed to form one comparator (AC/PE/PS) and no intervention, usual curriculum, and waiting list formed another control (NI/UC/WL). Reassuringly, the findings from Scenario Analysis 1 were more similar to Werner-Seidler’s and Zhang’s results. For targeted interventions, the SMD for cognitive-behavioral intervention relative to a “lumped” no intervention and usual curriculum control was SMD − 0.34 [95% CrI − 0.54, − 0.15] for depression and SMD − 0.21 [95% CrI − 0.39, − 0.04] for anxiety. For universal interventions, the SMD for cognitive-behavioral intervention relative to no intervention and usual curriculum was SMD − 0.06 [95% CrI − 0.11, − 0.02]) for anxiety and SMD − 0.06 [95% CrI: − 0.13, − 0.001] for depression. This suggests that “lumping” or conflating different control group types to form a single comparator for meta-analysis may improve precision of effect estimates. Indeed, the 95% CrI for the effect of cognitive-behavioral intervention relative to a reference control were more precisely estimated in both scenario analyses than in the main control group moderation analysis (“six distinct controls”).

### Implications for Future Meta-Analyses of Preventive Mental Health Interventions

The findings from Scenario Analysis 1 also raise an important question for future meta-analyses: Are the “statistically significant” beneficial effects of school-based interventions observed in previous pairwise meta-analyses an artefact of control group conflation? If so, then the categorization of control types in meta-analyses of preventive mental health interventions requires more explicit consideration than is currently given. Of the ten reviews identified for Scenario Analyses 1 and 2, only three provided definitions for each control type (Hetrick et al., 2016; Ssegonja et al., 2019; Werner-Seidler et al., 2021) and only one provided a detailed justification for combining controls into a single comparator (Werner-Seidler et al., 2021). Werner-Seidler explains that waiting list is “essentially another form of a no-intervention control group” because “the content of the no-intervention control groups and the wait-list groups more often than not involve school or class as usual across both control group types…” (Werner-Seidler et al., 2021, p.12). Findings from our main control group NMA (“six distinct controls”) in universal populations provide support for the similarity of no intervention, usual curriculum, and waiting list controls. However, our findings for targeted populations suggest that conflating controls in meta-analysis is not appropriate.

A challenge for meta-analyses is that trial reports may not contain sufficient detail to accurately classify control groups. Indeed, there is evidence from behavioral science that “as usual” comparators (e.g., usual care, usual class/curriculum) frequently contain “active” components that are not well-described in journal publications. In an analysis of behavior change interventions for smoking cessation, de Bruin et al. (2021) compared control group content from author provided materials with that available in the trial report publication and noted that only 26% of the control content could be identified from the published materials. They also found variability in the content of comparator/control groups that had been assigned the same categorization by trialists (e.g., ‘standard care’) (Black et al., 2020). The impact of control content variability on intervention effect estimates was examined via meta-regression models, with the conclusion that unreported control content and variability obscured “true” effectiveness of smoking cessation interventions (Kraiss et al., 2023).

A strength of our approach is that we did not rely solely on author classified control group type but applied a consistent classification scheme to all studies. Where there was insufficient or ambiguous detail reported in the paper, we consulted protocols and trial registrations and sought clarification from trialists via bespoke email requests. For example, clarifications sought for “waiting list” controlled studies included whether participants were aware they could access the intervention at the end of the study and what they received in the interim period. If waiting list participants could access usual curriculum, we additionally sought clarification of what that entailed and if it was standardized across schools. The response rate to all requests was 45% and too small to allow for further sensitivity analyses (for example, by differentiating between a waiting list where participants accessed usual curriculum and a waiting list where they received no intervention). Typical author responses noted usual curriculum usually had some wellbeing or social-emotional learning content, but that it varied across school sites (within study) and could not easily be summarized for reporting. There was no consensus among trialists whether it was appropriate to conflate usual curriculum with no intervention, some felt that they were exchangeable comparators, and some felt they were distinct. As such, we did not have sufficient data to replicate de Bruin’s analysis (Black et al., 2020; de Bruin et al., 2021; Kraiss et al., 2023).

Although our response rate was small, responses indicate an ambiguity about the purpose and operationalization of comparator/control groups in school-based prevention trials that limits the value of the evidence base in determining intervention effectiveness. Our results support Werner-Seidler’s suggestion that the content of no intervention, usual curriculum, and waiting list are probably similar enough to be combined for universal populations. However, we think it is unlikely that the content of usual curriculum and no intervention can be considered similar across universal and targeted populations. Other considerations include whether it is reasonable to assume that no intervention or usual curriculum controls are similar enough across different educational settings (e.g., primary, secondary), countries, or periods of time (e.g., 2000s, 2020s) to be conflated into a single comparator for meta-analysis. When planning meta-analyses of school-based prevention studies, researchers should carefully consider the appropriateness of conflating controls into a single comparator and provide explicit justification of their approach.

### Implications for Future Preventive Mental Health Intervention Trials

Cristea (2019) describes waiting list as an “inadequate benchmark” against which to assess the effectiveness of mental health interventions. In light of the potential for intervention effect to be modified by control group type or content, trialists should use active or attention control groups in preference to waiting list, usual class/curriculum, or no intervention controls. However, this may need to be balanced with the practical challenges of conducting school-based studies, ongoing concerns about young people’s mental health, and resource pressures. It seems likely that waiting list and “as usual” controls will continue to be preferred by schools and trialists (Dawson et al., 2018; Wheatley et al., 2020). As such, trialists might consider reporting down-weighted or adjusted results from waiting list-controlled studies alongside main effects (Hafliðadóttir et al., 2021; Sims et al., 2023). As well as contributing to the evidence base about control group moderation effects, this could be important information for school leaders and policymakers when commissioning mental health services in educational settings. A critical research recommendation is that trialists rigorously and accurately report the content provided to control groups and, in cluster-controlled trials, describe any variation in content across sites (Campbell et al., 2018; Hoffmann et al., 2017; Howick et al., 2020). For all the practical challenges of school-based research, it is important that the control group type and content is reported as extensively as the experimental intervention in trial registrations, protocols, and publications.

## Conclusion

Although exploratory, this paper adds to a growing body of evidence suggesting choice of control group in RCTs is important for interpreting the effectiveness of preventive and public health interventions. We did not find evidence of control group moderation effects for universal populations. However, for targeted populations, intervention effects were larger relative to waiting list controls than to no intervention or usual curriculum controls. Given the potential for effect estimates to be larger for waiting list-controlled studies, meta-analysts should justify decisions to conflate so-called inactive comparators, such as usual curriculum, no intervention, or waiting list when synthesizing RCTs of school-based anxiety and depression prevention interventions.

## Supplementary Information

Below is the link to the electronic supplementary material.

Supplementary file1 (DOCX 444 KB)

## Data Availability

Data are available from the author via https://research-information.bris.ac.uk/en/persons/deborah-m-caldwell/projects/
