# Supplementary material for: Exploring the Moderating Effect of Control Group Type on Intervention Effectiveness in School-Based Anxiety and Depression Prevention: Findings from a Rapid Review and Network Meta-analysis
Source: Prev Sci. 2025 Feb 12;26(2):175–92. doi: 10.1007/s11121-025-01786-y (PMC11891107; doi:10.1007/s11121-025-01786-y)
Supplement: Supplementary file 1 — Supplementary file1 (DOCX 444 KB) [file 11121_2025_1786_MOESM1_ESM.docx]

## Appendix/ Supplementary materials:

## Caldwell, DM et al. Exploring the moderating effect of control group type on intervention effectiveness in school-based anxiety and depression prevention

## Contents

[Search strategies 2](#_Toc188020754)

[Keyword ‘top-up’ search for RCTs: 01/01/2020 4](#_Toc188020755)

[Details of search for systematic reviews: 5](#_Toc188020756)

[Review team contributions and roles 7](#_Toc188020757)

[PRISMA extension checklist for Network Meta-Analysis 9](#_Toc188020758)

[Intervention and control definitions used in the analysis. 13](#_Toc188020759)

[Decision Rule for choosing between multiple scales reported within study 15](#_Toc188020760)

[Control group subgroup results reported by previous systematic reviews: Appendix Table 1 16](#_Toc188020763)

[WinBUGS code and dataset for full NMA and control group moderation analyses 18](#_Toc188020764)

[References to primary studies included in the review: 20](#_Toc188020765)

[Characteristics of included studies and risk of bias 1.0 assessments: Appendix Table 2 30](#_Toc188020766)

[Percentage of studies reporting each control group type, by 5-year periods: Appendix Table 3 36](#_Toc188020767)

[Full network diagrams: combined population and educational setting 37](#_Toc188020768)

[Separate educational setting and population specific networks 38](#_Toc188020769)

[Baseline and study level characteristics by comparison and analysis for assessment of transitivity: Appendix Table 4 40](#_Toc188020770)

[Model fit and selection statistics: Appendix Tables 5-8 44](#_Toc188020771)

[CINeMA assessment: judgements and reasons for downgrading: Appendix Tables 9-16 49](#_Toc188020772)

[Full NMA findings by setting and population: update compared with 2019 review. Tables 17-24 52](#_Toc188020773)

[Subgroup, meta-regression and sensitivity analyses for main NMA: Appendix Tables 25-38 55](#_Toc188020774)

[Intervention rankings for main control group analysis (six distinct control groups) 61](#_Toc188020775)

[Subgroup and sensitivity analyses for control group comparison of results from individually and cluster randomised studies. Appendix Tables 40-43 62](#_Toc188020776)

[Subgroup and sensitivity analyses for control group NMA: results excluding very small studies (n<50) Appendix Tables 44-47 64](#_Toc188020777)

[Sensitivity analysis: moderating effect of control group type for prevention of anxiety (bottom) and depression (top). Appendix Tables 48-49 65](#_Toc188020778)

[Sensitivity analysis: moderating effect of control group type for prevention of anxiety (bottom) and depression (top). 66](#_Toc188020779)

[Post hoc Scenario analyses 1 and 2 for control group NMA Appendix Tables 50-53 67](#_Toc188020780)

### Search strategies

The search strategy was adapted from the original search strategy from the 2019 review. The changes made were to remove the search terms related to conduct disorder. The search was limited to 01/01/2016 onwards and limited to publication type (review, systematic review, meta-analysis). The Medline search is reported below.

| 1. | CHILD, PRESCHOOL/ or CHILD/ or ADOLESCENT/ or YOUNG ADULT/ |
| --- | --- |
| 2. | (child* or boy* or girl* or kids or juvenil* or minors or paediatric* or pediatric* or adolesc* or preadolesc* or preadolesc* or pubert* or pubescen* or prepube* or prepube* or teen* or (young adj (adult* or people or patient* or men* or women* or male or female)) or youth* or student* or undergrad*).ti,ab,kf. |
| 3. | (child* or adolesc* or paediatr* or pediatr*).jn. |
| 4. | or/1-3 |
| 5. | EDUCATION/ |
| 6. | SCHOOLS/ or SCHOOLS, NURSERY/ |
| 7. | SCHOOL HEALTH SERVICES/ or SCHOOL NURSING/ |
| 8. | STUDENTS/ or UNIVERSITIES/ |
| 9. | (preschool or kindergarten or school* or college* or campus* or classroom* or curricul* or teacher or gatekeeper or pupil*).ti,ab,kf. |
| 10. | PEER GROUP/ |
| 11. | ((peer or peers) adj (education or group or relation* or support* or intervention* or leader*)).ti,ab,kf. |
| 12. | student* union.ti,ab,kf. |
| 13. | ((church or communit* or holiday* or religi* or spiritual* or youth or vacation) adj2 (camp or club or group)).ti,ab,kf. |
| 14. | ((church or communit* or holiday* or religi* or spiritual* or youth or vacation) adj based).ti,ab,kf. |
| 15. | or/5-14 |
| 16. | ADAPTATION, PSYCHOLOGICAL/ |
| 17. | EMOTIONS/ |
| 18. | MENTAL HEALTH/ |
| 19. | SOCIAL ADJUSTMENT/ |
| 20. | exp STRESS, PSYCHOLOGICAL/ |
| 21. | (mental health or mental* ill* or psychiatric).ti,ab,kf. |
| 22. | (wellbeing or well being).ti,ab,kf. |
| 23. | (stress* or distress*).ti,ab,kf. |
| 24. | or/16-23 |
| 25. | DEPRESSION/ |
| 26. | DEPRESSIVE DISORDER.mp. [mp=title, book title, abstract, original title, name of substance word, subject heading word, floating sub-heading word, keyword heading word, organism supplementary concept word, protocol supplementary concept word, rare disease supplementary concept word, unique identifier, synonyms, population supplementary concept word, anatomy supplementary concept word] |
| 27. | MOOD DISORDERS.mp. [mp=title, book title, abstract, original title, name of substance word, subject heading word, floating sub-heading word, keyword heading word, organism supplementary concept word, protocol supplementary concept word, rare disease supplementary concept word, unique identifier, synonyms, population supplementary concept word, anatomy supplementary concept word] |
| 28. | (depress* or dysthymi* or affective disorder* or affective symptom* or mood* or mental).ti. |
| 29. | (depress* adj2 (adolescent* or child* or anaclitic* or episode* or disorder or scale* or score* or symptom* or unipolar)).ti,ab,kf. |
| 30. | ((depress*or mood* or mental or psychological or wellbeing or well being or emotion*) adj2 (improve* or onset or prevent* or reduc*)).ti,ab,kf. |
| 31. | or/25-30 |
| 32. | exp ANXIETY DISORDERS/ |
| 33. | ANXIETY/ |
| 34. | anxi*.ti. |
| 35. | (anxi* adj3 (adolescent* or child* or disorder* or general* or interpersonal or separation or social*)).ti,ab,kf. |
| 36. | (phobi* or agoraphobi* or PTSD or post trauma* or posttrauma or panic* or OCD or obsess* or compulsi* or GAD or stress disorder* or stress reaction* or acute stress or neurosis or neuroses or neurotic or psychoneuro* or (school adj2 (refusal or avoid*)) or social avoidance or mutism).ti,ab,kf. |
| 37. | (((anxi* or fear or fright) adj3 (perform* or athlet* or music* or act* or test* or exam*)) or math* anxiety).ti,ab,kf. |
| 38. | (public adj3 (speak* or speech)).ti,ab,kf. |
| 39. | or/32-38 |
| 40. | PREVENTIVE HEALTH SERVICES/ or "EARLY INTERVENTION (education)"/ or HEALTH LITERACY/ or PATIENT EDUCATION AS TOPIC/ or HEALTH PROMOTION/ or PRIMARY PREVENTION/ or SECONDARY PREVENTION/ |
| 41. | prevention & control.fs. |
| 42. | prevent*.ti,kf. |
| 43. | prevention of.ab,kf. |
| 44. | (prevent* adj2 (intervention or educat* or pilot or program* or project or protocol* or training or universal or targeted or primary or secondary or selective or indicated or study or trial)).ti,ab,kf. |
| 45. | ((early or brief) adj intervention*).ti,ab,kf. |
| 46. | ((universal or targeted) adj2 (program* or intervention*)).ti,ab,kf. |
| 47. | or/40-46 |
| 48. | RANDOMIZED CONTROLLED TRIAL/ or PRAGMATIC CLINICAL TRIAL/ |
| 49. | Randomized Controlled Trial.pt. |
| 50. | (randomi#ed or randomi#ation).ab,ti,kf. |
| 51. | (RCT or (random* adj3 (administ* or allocat* or assign* or class* or cluster* or control* or determine* or divide* or distribut* or expose* or fashion or number* or place* or recruit* or subsitut* or treat*))).ab. |
| 52. | at random.ab. |
| 53. | placebo.ab. |
| 54. | trial.ti,kf. |
| 55. | or/48-54 |
| 56. | (treatmentasusual or (treatment* adj2 usual) or (standard adj2 care) or (standard adj2 treatment) or (routine adj2 care) or (usual adj2 medication*) or (usual adj2 care) or TAU).ti,ab,kf. |
| 57. | (waitlist* or waitlist* or waitinglist* or wait* list* or (waiting adj (condition or control)) or WLC).ti,ab,kf. |
| 58. | (((delay* adj3 (start or treatment*)) or no intervention or no treatment* or notreatment or non treatment* or nontreatment* or nontreatment or minim* treatment* or untreated group* or untreated control* or without any treatment) and (control* or group*)).ti,ab,kf. |
| 59. | ((no intervention* or non intervention* or nonintervention* or without any intervention*) and (control* or group*)).ti,ab,kf. |
| 60. | or/56-59 |
| 61. | 55 or 60 |
| 62. | ((universal or indicated or targeted or at risk) and prevent* and (anxiety or depress*) and (child* or adolesc* or school*)).mp. |
| 63. | ((prevent* adj (program* or intervention)) and (anxiety or depress*) and (child* or adolesc* or school*)).mp. |
| 64. | 62 or 63 |
| 65. | 4 and 15 and (24 or 31 or 39 or 47) and 61 |
| 66. | 64 and 61 |

### Keyword ‘top-up’ search for RCTs: 01/01/2020

Medline

| 1. | RANDOMIZED CONTROLLED TRIAL/ or PRAGMATIC CLINICAL TRIAL/ |
| --- | --- |
| 2. | Randomized Controlled Trial.pt. |
| 3. | (randomi#ed or randomi#ation).ab,ti,kf. |
| 4. | (RCT or (random* adj3 (administ* or allocat* or assign* or class* or cluster* or control* or determine* or divide* or distribut* or expose* or fashion or number* or place* or recruit* or subsitut* or treat*))).ab. |
| 5. | at random.ab. |
| 6. | trial.ti,kf. |
| 7. | or/1-6 |
| 8. | ((program* or intervention) and (mental adj health) and school*).ti,ab. |
| 9. | ((prevent* or reduc*) and (mental adj health) and school*).ti,ab. |
| 10. | ((prevent* or reduc*) and (depress* or anxi*) and school*).ti,ab. |
| 11. | ((universal or indicated or targeted or risk) and (prevent* or intervention) and (anxiety or depress*) and school*).ti,ab. |
| 12. | ((prevent* or reduc*) and (mental adj health) and school* and intervention).ti,ab. |
| 13. | or/8-12 |
| 14. | 6 and 13 |

### Details of search for systematic reviews:

- The search for reviews returned 695 citations (Medline: 353. PsycINFO: 169. Embase: 173)
- After de-duplication, the titles and abstracts of 625 citations were screened.
- 44 reports were full-text screened.
- 18 reports were excluded (*Reasons*: review published before 2018 (9), wrong population focus (1), paper from original review (2), not retrieved (1), wrong setting (3), wrong outcome (2).
- 26 reviews included, from which 758 references of primary studies were retrieved for screening.
- 477 study references were published before 2018 and were excluded before screening, as this overlapped with previous searches for Caldwell et al, 2021 (original review). See Figure 2 in main manuscript, PRISMA flow chart.

##### References to 26 identified reviews:

Arora P, Collins TA, Dart EH, Hernández S, Fetterman H, Doll B. Multi-tiered Systems of Support for School-Based Mental Health: A Systematic Review of Depression Interventions. *School Mental Health* 2019;11:240-64.

Bradshaw M, Gericke H, Coetzee B, Stallard P, Human S, Loades M. Universal school-based mental health programmes in low- and middle-income countries: A systematic review and narrative synthesis. *Preventive medicine* 2020;143:106317-NA.

Choi E, Yoon E-H, Park M-H. Game-based digital therapeutics for children and adolescents: Their therapeutic effects on mental health problems, the sustainability of the therapeutic effects and the transfer of cognitive functions. *Frontiers in psychiatry* 2022;13:986687-NA.

Egan SJ, Rees CS, Delalande J, Greene D, Fitzallen G, Brown S, et al. A Review of Self-Compassion as an Active Ingredient in the Prevention and Treatment of Anxiety and Depression in Young People. *Administration and policy in mental health* 2021;49:1-19

Gijzen MWM, Rasing SPA, Creemers DHM, Engels RCME, Smit F. Effectiveness of school-based preventive programs in suicidal thoughts and behaviors: A meta-analysis. *Journal of affective disorders* 2021;298:408-20.

Feiss R, Dolinger SB, Merritt M, Reiche E, Martin K, Yanes JA, et al. A Systematic Review and Meta-Analysis of School-Based Stress, Anxiety, and Depression Prevention Programs for Adolescents. *Journal of youth and adolescence* 2019;48:1668-85.

Filia K, Eastwood O, Herniman SE, Badcock PB. Facilitating improvements in young people's social relationships to prevent or treat depression: A review of empirically supported interventions. *Translational psychiatry* 2021;11:305.

Hoare E, Collins S, Marx W, Callaly E, Moxham-Smith R, Cuijpers P, et al. Universal depression prevention: An umbrella review of meta-analyses. *Journal of psychiatric research* 2021;144:483-93

Hugh-Jones S, Beckett S, Tumelty E, Mallikarjun P. Indicated prevention interventions for anxiety in children and adolescents: a review and meta-analysis of school-based programs. *European child & adolescent psychiatry* 2020;30:849-60

Johnstone KM, Kemps E, Chen J. A Meta-Analysis of Universal School-Based Prevention Programs for Anxiety and Depression in Children. *Clinical child and family psychology review* 2018;21:466-81.

Jones RB, Thapar A, Stone Z, Thapar AK, Jones I, Smith DJ, et al. Psychoeducational interventions in adolescent depression: A systematic review. *Patient education and counselling* 2017;101:804-16.

Li J, Liang J-H, Li J-y, Qian S, Jia R-x, Wang Y-q, et al. Optimal approaches for preventing depressive symptoms in children and adolescents based on the psychosocial interventions: A Bayesian Network Meta-Analysis. *Journal of affective disorders* 2020;280:364-72

Mackenzie K, Williams C. Universal, school-based interventions to promote mental and emotional well-being: what is being done in the UK and does it work? A systematic review. *BMJ open* 2018;8:e022560-NA.

Martinez K, Menéndez-Menéndez MI, Bustillo A. Awareness, Prevention, Detection, and Therapy Applications for Depression and Anxiety in Serious Games for Children and Adolescents: Systematic Review. *JMIR serious games* 2021;9:e30482-e.

de Pablo GS, De Micheli A, Solmi M, Oliver D, Catalan A, Verdino V, et al. Universal and Selective Interventions to Prevent Poor Mental Health Outcomes in Young People: Systematic Review and Meta-analysis. *Harvard review of psychiatry* 2021;29:196-215.

Pascoe MC, Parker AG. Physical activity and exercise as a universal depression prevention in young people: A narrative review. *Early intervention in psychiatry* 2018;13:733-9.

Phan ML, Renshaw TL, Caramanico J, Greeson JM, MacKenzie E, Atkinson-Diaz Z, et al. Mindfulness-based school interventions: A systematic review of outcome evidence quality by study design. *Mindfulness* 2022;13:1591-613.

Rohde P, Brière FN, Stice E. Major depression prevention effects for a cognitive-behavioral adolescent indicated prevention group intervention across four trials. *Behaviour research & therapy* 2017;100:1-6.

Schwartz C, Barican J, Yung D, Zheng Y, Waddell C. Six decades of preventing and treating childhood anxiety disorders: a systematic review and meta-analysis to inform policy and practice. *Evidence-based mental health* 2019;22:103-10.

Šouláková B, Kasal A, Butzer B, Winkler P. Meta-Review on the Effectiveness of Classroom-Based Psychological Interventions Aimed at Improving Student Mental Health and Well-Being and Preventing Mental Illness. *The journal of primary prevention* 2019;40:255-78.

Ssegonja R, Nystrand C, Feldman I, Sarkadi A, Langenskiöld S, Jonsson U. Indicated preventive interventions for depression in children and adolescents: A meta-analysis and meta-regression. *Preventive medicine* 2018;118:7-15.

Tozzi F, Nicolaidou I, Galani A, Antoniades A. eHealth Interventions for Anxiety Management Targeting Young Children and Adolescents: Exploratory Review. JMIR pediatrics and parenting 2018;1:e5.

Vallis EH, Zwicker A, Uher R, Pavlova B. Cognitive-behavioural interventions for prevention and treatment of anxiety in young children: A systematic review and meta-analysis. *Clinical psychology review* 2020;81:101904.

Waldron S, Stallard P, Grist R, Hamilton-Giachritsis C. The ‘Long-Term’ Effects of Universal School-based Anxiety Prevention Trials: A Systematic Review. *Mental Health & Prevention* 2018;11:8-15

Werner-Seidler A, Spanos S, Calear AL, Perry Y, Torok M, O'Dea B, et al. School-based depression and anxiety prevention programs: An updated systematic review and meta-analysis. *Clinical psychology review* 2021;89:102079.

Zhang Q, Wang J, Neitzel A. School-based Mental Health Interventions Targeting Depression or Anxiety: A Meta-analysis of Rigorous Randomized Controlled Trials for School-aged Children and Adolescents. *Journal of youth and adolescence* 2022;52:195-217.

### Review team contributions and roles

| **Name** | **ORCiD** |
| --- | --- |
| Deborah Caldwell | 0000-0001-8014-7480 |
| Jennifer Palmer | 0000-0002-8073-2505 |
| Katie Webster | 0009-0002-7997-4133 |
| Sarah Davies | 0000-0003-1321-7826 |
| Hugo Hughes | NA |
| Joseph Rona | NA |
| Rachel Churchill | 0000-0002-1751-0512 |
| Sarah Hetrick | 0000-0003-2532-0142 |
| Nicky Welton | 0000-0003-2198-3205 |

DC co-conceived the study design, drafted the protocol, sifted references, identified eligible studies, extracted data, and assessed risk of bias for all included studies, conducted statistical analyses, supervised the project, and drafted the manuscript.

JP co-conceived the study design, edited the update review manuscript, and extracted data and assessed risk of bias for the 126 studies identified by the original review.

KW identified eligible studies, extracted data, assessed risk of bias, and provided methodological advice on the update review, and edited the manuscript.

SD contributed to intervention classification for the update review, provided methodological advice, and edited the manuscript, identified eligible studies, extracted data, and assessed risk of bias for the 126 studies identified by the original review.

HH sifted references, identified eligible studies, and extracted data for the 26 studies identified in the update review.

JR sifted references, identified eligible studies, and extracted data for the 26 studies identified in the update review.

RC provided methodological advice, contributed to the development of the intervention classification scheme and reviewed the manuscript

SH provided methodological advice, contributed to the development of the intervention classification scheme and reviewed the manuscript

NW co-conceived the study design, provided methodological advice, developed the WinBUGS code, advised on and supported the statistical analyses, and edited the review manuscript.

Study selection for the update review was independently assessed by two reviewers (of DC, HH, JR) and disagreement resolved by a third (KW), if necessary. For the 26 newly identified primary studies, data extraction, intervention classification, and risk of bias (RoB) assessment was completed by one reviewer. Data extraction of baseline and post-intervention N, means and standard deviations was done independently and in duplicate for 4 of the 26 studies by DC, HH and JR. Then, for the remaining studies extraction was completed by one of HH, JR or DC and was verified by DC or KW (KW verified any extraction by DC). Extraction of study characteristics, intervention classification, and risk of bias assessment were conducted by either DC or KW and then verified by the other. Intervention classification discrepancies were discussed with SD. Analyses were conducted by DC, with support from NW.

HH, JR and KW were not involved in the original 2019/ 2021 original review. The roles of RC, SD, SH, JP and NW in the original review are described in full in both the 2019 and 2021 publications. Briefly, RC, SD and SH contributed to the development and piloting of the intervention and control classifications. SD and JP contributed to data extraction, intervention classification, and risk of bias assessment. JP contributed to statistical analyses. Development of WinBUGS code and supervision of statistical analyses was provided by NW. This applies for the 126 studies included in the current update review but which were identified from the pre- April 2018 searches.

### PRISMA extension checklist for Network Meta-Analysis

Hutton, B., et al., *The PRISMA extension statement for reporting of systematic reviews incorporating network meta-analyses of health care interventions: checklist and explanations.* Ann Intern Med, 2015. **162**(11): p. 777-84.

Checklist available from: <https://www.prisma-statement.org/nma> [Accessed 30-04-2024]

| **Section/Topic** | **Item #** | **Checklist Item** | **Reported on Page # of author’s submitted manuscript** |
| --- | --- | --- | --- |
| **TITLE** |  |  |  |
| Title | 1 | Identify the report as a systematic review *incorporating a network meta-analysis (or related form of meta-analysis).* | 1 |
| **ABSTRACT** |  |  |  |
| Structured summary | 2 | Provide a structured summary including, as applicable:  **Background:** main objectives  **Methods:** data sources; study eligibility criteria, participants, and interventions; study appraisal; and *synthesis methods, such as network meta-analysis.*  **Results:** number of studies and participants identified; summary estimates with corresponding confidence/credible intervals; *treatment rankings may also be discussed. Authors may choose to summarize pairwise comparisons against a chosen treatment included in their analyses for brevity.*  **Discussion/Conclusions:** limitations; conclusions and implications of findings.  **Other:** primary source of funding; systematic review registration number with registry name. | 2 |
| **INTRODUCTION** |  |  |  |
| Rationale | 3 | Describe the rationale for the review in the context of what is already known*, including mention of why a network meta-analysis has been conducted.* | 3-5 |
| Objectives | 4 | Provide an explicit statement of questions being addressed, with reference to participants, interventions, comparisons, outcomes, and study design (PICOS). | 5 |
|  |  |  |  |
| **METHODS** |  |  |  |
| Protocol and registration | 5 | Indicate whether a review protocol exists and if and where it can be accessed (e.g., Web address); and, if available, provide registration information, including registration number. | 6 |
| Eligibility criteria | 6 | Specify study characteristics (e.g., PICOS, length of follow-up) and report characteristics (e.g., years considered, language, publication status) used as criteria for eligibility, giving rationale. *Clearly describe eligible treatments included in the treatment network, and note whether any have been clustered or merged into the same node (with justification).* | 6 |
| Information sources | 7 | Describe all information sources (e.g., databases with dates of coverage, contact with study authors to identify additional studies) in the search and date last searched. | 5 |
| Search | 8 | Present full electronic search strategy for at least one database, including any limits used, such that it could be repeated. | Appendix |
| Study selection | 9 | State the process for selecting studies (i.e., screening, eligibility, included in systematic review, and, if applicable, included in the meta-analysis). | 6 |
| Data collection process | 10 | Describe method of data extraction from reports (e.g., piloted forms, independently, in duplicate) and any processes for obtaining and confirming data from investigators. | 6 |
| Data items | 11 | List and define all variables for which data were sought (e.g., PICOS, funding sources) and any assumptions and simplifications made. | 6-7 |
| *Geometry of the network* | **S1** | Describe methods used to explore the geometry of the treatment network under study and potential biases related to it. This should include how the evidence base has been graphically summarized for presentation, and what characteristics were compiled and used to describe the evidence base to readers. | NA (not a mandatory item) |
| Risk of bias within individual studies | 12 | Describe methods used for assessing risk of bias of individual studies (including specification of whether this was done at the study or outcome level), and how this information is to be used in any data synthesis. | 6 (and Appendix) |
| Summary measures | 13 | State the principal summary measures (e.g., risk ratio, difference in means). *Also describe the use of additional summary measures assessed, such as treatment rankings and surface under the cumulative ranking curve (SUCRA) values, as well as modified approaches used to present summary findings from meta-analyses.* | 7 |
| Planned methods of analysis | 14 | Describe the methods of handling data and combining results of studies for each network meta-analysis. This should include, but not be limited to:   - *Handling of multi-arm trials;* - *Selection of variance structure;* - *Selection of prior distributions in Bayesian analyses; and* - *Assessment of model fit.* | 7-8 |
| *Assessment of Inconsistency* | **S2** | Describe the statistical methods used to evaluate the agreement of direct and indirect evidence in the treatment network(s) studied. Describe efforts taken to address its presence when found. | 7-10 |
| Risk of bias across studies | 15 | Specify any assessment of risk of bias that may affect the cumulative evidence (e.g., publication bias, selective reporting within studies). | 6 |
| Additional analyses | 16 | Describe methods of additional analyses if done, indicating which were pre-specified. This may include, but not be limited to, the following:   - Sensitivity or subgroup analyses; - Meta-regression analyses; - *Alternative formulations of the treatment network; and* - *Use of alternative prior distributions for Bayesian analyses (if applicable).* | 8-9 |
| **RESULTS†** |  |  |  |
| Study selection | 17 | Give numbers of studies screened, assessed for eligibility, and included in the review, with reasons for exclusions at each stage, ideally with a flow diagram. | 9-10 and Fig 2 |
| *Presentation of network structure* | **S3** | Provide a network graph of the included studies to enable visualization of the geometry of the treatment network. | Fig 1 and Appendix |
| *Summary of network geometry* | **S4** | Provide a brief overview of characteristics of the treatment network. This may include commentary on the abundance of trials and randomized patients for the different interventions and pairwise comparisons in the network, gaps of evidence in the treatment network, and potential biases reflected by the network structure. | NA (Not a mandatory item) |
| Study characteristics | 18 | For each study, present characteristics for which data were extracted (e.g., study size, PICOS, follow-up period) and provide the citations. | Appendix |
| Risk of bias within studies | 19 | Present data on risk of bias of each study and, if available, any outcome level assessment. | Appendix |
| Results of individual studies | 20 | For all outcomes considered (benefits or harms), present, for each study: 1) simple summary data for each intervention group, and 2) effect estimates and confidence intervals. *Modified approaches may be needed to deal with information from larger networks.* | Appendix |
| Synthesis of results | 21 | Present results of each meta-analysis done, including confidence/credible intervals. *In larger networks, authors may focus on comparisons versus a particular comparator (e.g. placebo or standard care), with full findings presented in an appendix. League tables and forest plots may be considered to summarize pairwise comparisons.* If additional summary measures were explored (such as treatment rankings), these should also be presented. | 9-14 and Appendix |
| *Exploration for inconsistency* | **S5** | Describe results from investigations of inconsistency. This may include such information as measures of model fit to compare consistency and inconsistency models, *P* values from statistical tests, or summary of inconsistency estimates from different parts of the treatment network. | Appendix |
| Risk of bias across studies | 22 | Present results of any assessment of risk of bias across studies for the evidence base being studied. | Appendix |
| Results of additional analyses | 23 | Give results of additional analyses, if done (e.g., sensitivity or subgroup analyses, meta-regression analyses*, alternative network geometries studied, alternative choice of prior distributions for Bayesian analyses,* and so forth). | 11-14 and Appendix |
|  |  |  |  |
| **DISCUSSION** |  |  |  |
| Summary of evidence | 24 | Summarize the main findings, including the strength of evidence for each main outcome; consider their relevance to key groups (e.g., healthcare providers, users, and policy-makers). | 14-16 |
| Limitations | 25 | Discuss limitations at study and outcome level (e.g., risk of bias), and at review level (e.g., incomplete retrieval of identified research, reporting bias). *Comment on the validity of the assumptions, such as transitivity and consistency. Comment on any concerns regarding network geometry (e.g., avoidance of certain comparisons).* | 14-16 |
| Conclusions | 26 | Provide a general interpretation of the results in the context of other evidence, and implications for future research. | 14-19 |
| **FUNDING** |  |  |  |
| Funding | 27 | Describe sources of funding for the systematic review and other support (e.g., supply of data); role of funders for the systematic review. This should also include information regarding whether funding has been received from manufacturers of treatments in the network and/or whether some of the authors are content experts with professional conflicts of interest that could affect use of treatments in the network. | 18 |

### Intervention and control definitions used in the analysis.

| **Name** | **Definition used in this study to categorise interventions and control types** | |
| --- | --- | --- |
| *Usual curriculum* | \| *Universal*: the experimental intervention takes place during a regular timetabled class, or school schedule, and participants in the control group receive that regular or usual/ standard class. This could be a range of different classes e.g. Physical Education, PSHE, Health Education, or academic subjects. \| \| --- \| \| *Targeted*: intervention typically takes place outside of scheduled classes or is in addition to scheduled classes and is not part of the school’s regular timetable. Usual curriculum/practice refers to the usual route by which young people can access mental health support in school. This could be through a school counsellor or teacher but does not include no intervention. \| |  |
| *Waiting List* | Where participants (schools, parents, or children) were explicitly told that they would receive the active intervention at a later date. Participants may also be receiving usual curriculum or a no intervention control during the waiting period, and this should also be extracted if clear. |  |
| *No intervention* | *Universal*: where the intervention was held outside of regular classes (e.g. after school or at lunchtime) or the intervention happens outside of school time. The participants aren’t described as being in a wait list.  *Targeted*: where intervention is held outside of regular classes (e.g. after school or at lunchtime) or intervention happens outside of school time. The participants aren’t described as being in a wait list, there is no in school mental health or counselling provision or were not permitted to seek treatment outside of the study. |  |
| *Attention control* | Where a de novo non-psychological intervention is provided to the participants for the purposes of trial. E.g. putting on a brand-new class developed specifically for the study. |  |
| *Psycho-social & supportive (includes counselling)* | *Universal*: may be used to resemble an 'active' usual provision comparator, like SEL, but may have been standardised for purposes of study. May contain discussion of feelings, emotions, and may allow forming an attachment or ‘therapeutic’ alliance.  *Targeted*: includes non-specific therapeutic components which might include listening, signposting to further services, forming an attachment or therapeutic alliance. PS classification should not contain cognitive or behavioural aspects associated with other psychotherapeutic techniques. |  |
| *Psycho-education/ education* | Often a component of the active psychological intervention. Here a standalone educational approach to providing background information regarding depression or anxiety and providing signposting to support services and/or the ways in which an intervention might help. May be used to resemble a usual provision intervention, but may have been standardised for the purposes of trial. |  |
| *Cognitive behavioural* | Interventions based on a group of overlapping cognitive and behavioural techniques. Here, we took an inclusive approach, e.g. if an author labelled an intervention as Cognitive Behavioural (CBT) but only used a single component (e.g. cognitive restructuring) we categorised the intervention as based on CB principles. |  |
| *Behavioural* | Behavioural Therapy is a group of allied techniques that focus on behavioural models of psychology and seek to modify maladaptive behaviours or model positive, pro-social behaviours. E.g. interventions based on behavioural activation, self-monitoring, role-playing, scheduling pleasant activities, social skills development. |  |
| *Third wave* | Third wave psychotherapies combine principles of CBT and principles of mindfulness, acceptance and flexibility and have been adapted for prevention. To be categorised as third wave, the intervention should focus on modifying the function of thoughts rather than on modifying their content. |  |
| *Mindfulness/ Relaxation* | A combined category. Relaxation includes breathing exercises, muscle relaxation and yoga from the Iyengar or Hatha traditions (as opposed to e.g. Vinyasa or Bikram traditions). Mindfulness interventions were included here if they primarily focus on mindfulness, without aspects of CBT or other psychotherapeutic approaches (see Third wave for mindfulness-based CBT). |  |
| *Interpersonal* | Techniques which primarily focus on addressing the relationship between young people and significant adults (e.g., teachers, parents), with regards avoiding/ resolving conflict via improved communication skills. |  |
| *Biofeedback* | A mind-body intervention which uses instruments to learn to control physiological responses, such as heart rate. |  |
| *Exercise* | Cardiovascular interventions designed to raise heart rate and breathing into (at least) the moderate intensity level. |  |
| *Bias/ cognitive modification* | Post-hoc identified category: applied cognitive processing therapies often delivered via computer. Includes attention bias and interpretation bias training. | |
| *Positive psychology* | Post-hoc identified & combined category: interventions focus on enhancing positive and optimistic perspective rather than modifying negative outlook. E.g. growth mindset, gratitude, optimism and resilience. | |

### Decision Rule for choosing between multiple scales reported within study

The decision rules below were developed for Caldwell et al (2019) and Caldwell et al (2021) and are described verbatim from those original publications. These rules were applied for selecting an outcome measurement scale for use in the network meta-analyses, where a study reported multiple validated scales for assessing and reporting either depression or anxiety. Only one scale per study was included in the NMA reported in the present manuscript. For the 38 studies identified in the update review, only the prioritised scale was extracted. However, for the 126 studies included in the original review, data from all validated scales were extracted.

Decision Rule for Depression Scales

1. Scores that combine depression and other symptoms will be excluded (e.g. scales which measure ‘internalising symptoms’ or combined anxiety and depression scores).

2. Choice between multiple scales

1. Use self-reports in preference to clinician-rated scales.
2. Use instruments with well-studied psychometric properties.
3. Use inventories aimed at paediatric populations in preference to inventories aimed at general population.
4. Use instruments specifically targeted to measure depressive symptoms in preference to instruments with a broader scope.
5. Use most commonly reported scale across studies.

Decision Rule for Anxiety Scales

1. Scores that combine anxiety and other symptoms will be excluded (e.g. total RCADS would be excluded as it is a combined depression and anxiety score. Whereas the RCADS total anxiety subscale would be included in preference)

2. Use total anxiety scores where available

1. If total anxiety score is not available, but e.g. a generalised anxiety subscale is reported, we will use the subscale (for universal populations we think most interventions are likely to be targeting non-specific anxiety and are not sure what the importance of separation and social anxiety are. And some other subscales e.g. PTSD, OCD are no longer considered anxiety disorders in DSM 5)

3. Choice between multiple scales:

1. Use inventories of general symptoms in preference to instruments targeting specific anxiety domains.
2. If several inventories of general symptoms are available, use those aimed at general population in preference to instruments aimed at identifying patients with anxiety disorders.
3. Use most commonly reported scale across studies

### Control group subgroup results reported by previous systematic reviews: Appendix Table 1

| **Author** | **Focus** | **Population** | **Setting** | **Intervention** | **N studies** | **Lumped control for MA?** | **Subgroup results by control group (relative to 'active' ctrl)** | **Control group definition** |
| --- | --- | --- | --- | --- | --- | --- | --- | --- |
| Teubert 2011 | A | U+T | NC | Psychological, educational, physical | 59 | Y | WL/NI (k=53). g: 0.25 (0.16 to 0.34) AC (k=12). g: 0.11 (-0.08 to 0.30) | Included *“waitlist control or active control groups such as attention control and/or placebo interventions)”; “Passive waitlist control groups only control for the passage of time…”* (P.1047) |
| Stockings 2016 | A+D | U+T | Any | Psychological, educational, physical | 146 | Y | Universal t52 = 0.54, p = 0.53, R2 = −0.84% Selective: t33 = −0.48, p = 0.63, R2 = −10.58% Indicated: t43 = 1.71, p = 0.09, R2 = 8.58% | *“…studies used a no-intervention comparator (n = 123; either treatment as usual, wait-listed control, monitoring control, or no intervention), with 23 employing active comparators (including attention and placebo controls)”* (P.16) |
| Hetrick 2016 | D | U+T | Any | Psychological | 83 | Y | **Universal**: Test for subgroup difference: p=0.11 TAU (k=9). SMD: -0.15 (-0.31 to 0.00)  NI (k=9) SMD: -0.15 (-0.25 to -0.05)  AC (k=9) SMD: 0.00 (-0.09 to 0.08)  WL (k=4) SMD: -0.12 (-0.28 to 0.04)  **Targeted**: Test for subgroup difference: p=0.14 TAU (k=16). SMD: -0.30 (-0.45 to -0.15)  NI (k=14) SMD: -0.39 (-0.57 to -0.21)  AC (k=4) SMD: -0.10 (-0.32 to 0.13)  WL (k=6) SMD: -0.49 (-0.72 to -0.26) | *“ The comparison groups … in order of increasing rigorousness: • treatment as usual, defined as the normal healthcare curriculum, physical education classes or the ability to access any school-based and/or external mental health care as required; • no treatment; • wait-list; • attention placebo, defined by Merry 2006 (p.178) as “controlling for non-specific factors….* (P.10) |
| Lawrence 2017 | A | T | Any | NC | 16 | Y | Inactive (k=10) SMD: -0.43 (-0.73 to -0.12) | *“a waitlist and/or an active comparison condition.” (*P.120) |
| Werner-Seidler 2017 (& 2021) | A+D | U+T | School | Psychological, Educational | 81 | Y | **Depression**:  WL B: 0.10 (SE 0.08), AC B: -0.04 (SE 0.07).  NI (k=47), g=0.22, (0.16 to 0.27) WL (k=8), g=0.36, (0.16 to 0.56) AC (k=19), g = 0.24, (0.13 to 0.35) | Waiting list is *“essentially another form of a no-intervention control group”. “the content of the no-intervention control groups and the wait-list groups more often than not involve school or class as usual across both control group types…* *we*  *collapsed these two categories into a broad ‘inactive control group’”*(P.12) |
|  |  |  |  |  |  |  | **Anxiety**:  WL B: 0.05 (SE 0.07), AC B: -0.01 (SE 0.08). WL (k=16), g = 0.29, (0.16 to 0.43) NI (k=23), g = 0.19, (0.11 to 0.26) AC (k=10), g = 0.10, (0.01 to 0.19) |  |
| Moreno-Peral 2017 | A | U+T | Any | Psychological, Educational | 9 | Y | Q=30.295; d.f.(Q)=2; = <0.001 UC/NI: (k=19) SMD: -0.17 (-0.25 to -0.09) p= <.001 AC: (k=8) SMD: -0.30 (-0.55 to -0.06), p= 0.01  WL (k=9) SMD: -0.69 (-0.87 to -0.53) p= <.001 | *“… comparators allowed were care-as-usual, no intervention, a waiting list for intervention, or attention control.”* (P.1022) |
| Feiss 2019 | A+D | U+T |  | Psychological |  | Y | Anxiety: *"… control condition did not reveal any factors that significantly influenced the reduction in anxiety symptoms"*( P.1677)*.* Depression: no result | *“…control condition (i.e., active or non-active)”* (P.1671) |
| Hugh-Jones 2021 | A | T | School | Psychological, educational | 20 | Y | Chi2 = 4.85, df = 2, P = 0.09,  WL (k = 9), g = -0.53, (-0.97 to -0.09)  AC: (k = 5), g = -0.00 (-0.18 to 0.17)  NI: (k = 4), g = -0.10, (-0.44 to 0.24) | *“Comparator: passive comparators (waitlist and no intervention) and active comparators (programs to control for non-specific aspects of anxiety treatment)”.* (P.851) |
| Zhang 2023 | A+D | U+T |  | Psychological, educational, physical | 29 | Y | *"In exploratory analysis, an additional moderator called waitlist is included in the meta-regression model…. After adding this moderator, estimates and significance values were broadly similar to the original results."* | *“… business as usual or waitlist, which are considered as inactive group.”* (P.207) |
| Ssegonja 2018 | D | T |  | Group-based cognitive behavioral | 38 | Y | Passive 0.005 (0.99 - 1.88) p= 0.06 | *“Active comparator i.e. other specified preventive treatments for depression or an intervention designed to control for non-specific aspects of treatment for depression (e.g. group counselling and bibliotherapy) and passive comparators e.g. waitlist and treatment as usual (assessment only control with the participants free to seek care).” (*P.8) |

### WinBUGS code and dataset for full NMA and control group moderation analyses

Code adapted from: S Dias, NJ Welton, A Sutton, AE Ades (2016). NICE DSU technical support document 2: a generalised linear modelling framework for pairwise and network meta-analysis of randomised controlled trials. Document is available from: https://www.sheffield.ac.uk/nice-dsu/tsds/evidence-synthesis [Accessed 15-01-2025]. An example dataset is provided at the end of the code for illustration. The NMA dataset is available as a .csv file from https://research-information.bris.ac.uk/en/persons/deborah-m-caldwell/projects/

model{

for(i in 1:ns){

w[i,1] <- 0 # adjustment for multi-arm trials is zero for control arm

delta[i,1] <- 0 # treatment effect is zero for control arm

mu[i] ~ dnorm(0,.0001) # vague priors for all trial baselines

for (k in 1:na[i]) { # LOOP THROUGH ARMS

var[i,k] <- pow(se[i,k],2) # calculate variances

prec[i,k] <- 1/var[i,k] # set precisions

y[i,k] ~ dnorm(phi[i,k],prec[i,k]) # normal likelihood

phi[i,k]<-theta[i,k]*(pooled.sd[i]/J[i]) #Hedges G adjustment/ SMD

theta[i,k] <- mu[i] + delta[i,k] # model for linear predictor

Dnum[i,k] <- (y[i,k]-phi[i,k])*(y[i,k]-phi[i,k])

dev[i,k] <- Dnum[i,k]/var[i,k] #deviance arm k, study i

}

resdev[i] <- sum(dev[i,1:na[i]]) # summed residual deviance contribution for this trial

for (k in 2:na[i]) { # LOOP THROUGH ARMS

delta[i,k] ~ dnorm(md[i,k],taud[i,k]) # trial-specific treatment effect distributions

md[i,k] <- d[treat[t[i,k]]] - d[treat[t[i,1]]] + sw[i,k] # mean of treat effects distributions (with multi-arm trial correction)

taud[i,k] <- tau *2*(k-1)/k # precision of trt effects distributions (with multi-arm trial correction)

w[i,k] <- (delta[i,k] - d[treat[t[i,k]]] + d[treat[t[i,1]]]) # multi-arm adjustment, with additional code [treat] for recoding interventions

sw[i,k] <- sum(w[i,1:k-1])/(k-1) # cumulative adjustment for multi-arm trials

}}

totresdev <- sum(resdev[]) #Total Residual Deviance

d[1]<-0 #treatment effect is zero for reference treatment

for (k in 2:nt){ d[k] ~ dnorm(0,.0001) } # vague priors for treatment effects

sd ~ dunif(0,10) # vague prior for between-trial SD

tau <- pow(sd,-2) # between-trial precision = (1/between-trial variance)

for (c in 1:(nt-1)) { # SMDs for each comparison

for (k in (c+1):nt) {

smd[c,k] <- (d[k]-d[c]) } } }}

**Example data:**

| No Intervention: | Treatment 1 (reference treatment) |
| --- | --- |
| Waiting list: | Treatment 2 |
| Attention control: | Treatment 3 |
| CB: | Treatment 4 |
| Education: | Treatment 5 |

list(nt=5, ns=19, treat=c(1,2,3,4,5)) #Standard NMA, for 5 ‘treatments’(NI is reference)

list(nt=5, ns=19, treat=c(2,1,3,4,5)) #As above but changing reference to waiting list

list(nt=3, ns=19, treat=c(1,2,1,3,1)) #Conflating control types into a (i) no intervention + waiting list and an (ii) attention + education node

| t[,1] | y[,1] | se[,1] | t[,2] | y[,2] | se[,2] | t[,3] | y[,3] | se[,3] | t[,4] | y[,4] | se[,4] | pooled.sd[] | J[] | na[] |
| --- | --- | --- | --- | --- | --- | --- | --- | --- | --- | --- | --- | --- | --- | --- |
| 2 | -2.66 | 0.59539 | 4 | -3.35 | 0.42823 | NA | NA | NA | NA | NA | NA | 6.26551 | 0.99545 | 2 |
| 2 | -10.89 | 1.73811 | 4 | -12.58 | 1.34146 | NA | NA | NA | NA | NA | NA | 11.83463 | 0.98997 | 2 |
| 2 | 1.91 | 1.84717 | 4 | -6.87 | 1.51571 | NA | NA | NA | NA | NA | NA | 9.96722 | 0.98286 | 2 |
| 1 | -1.68 | 0.52161 | 4 | -0.76 | 0.47401 | NA | NA | NA | NA | NA | NA | 4.59254 | 0.99685 | 2 |
| 1 | -1.74 | 0.55052 | 4 | -1.51 | 0.49592 | 4 | -2.44 | 0.49686 | NA | NA | NA | 7.46785 | 0.99815 | 3 |
| 1 | -6.17 | 1.53360 | 4 | -6.83 | 1.57634 | 4 | -5.93 | 1.65351 | 4 | -5.25 | 1.43861 | 17.82526 | 0.99855 | 4 |
| 2 | -0.25 | 0.11779 | 4 | -0.71 | 0.13925 | NA | NA | NA | NA | NA | NA | 1.47513 | 0.99768 | 2 |
| 3 | -4.15 | 1.57311 | 4 | -3.72 | 1.25748 | NA | NA | NA | NA | NA | NA | 9.16009 | 0.98286 | 2 |
| 1 | 1.33 | 0.69744 | 4 | 1.88 | 0.92779 | NA | NA | NA | NA | NA | NA | 9.25482 | 0.99637 | 2 |
| 3 | -3.33 | 2.95835 | 4 | -6.43 | 3.42072 | NA | NA | NA | NA | NA | NA | 9.85366 | 0.95775 | 2 |
| 4 | -0.31 | 0.07236 | 5 | -0.24 | 0.10834 | NA | NA | NA | NA | NA | NA | 0.51696 | 0.98955 | 2 |
| 4 | -4.01 | 1.18037 | 5 | -3.13 | 1.25961 | NA | NA | NA | NA | NA | NA | 10.69842 | 0.99413 | 2 |
| 2 | -5.02 | 1.61773 | 4 | -10.67 | 1.14131 | NA | NA | NA | NA | NA | NA | 18.62407 | 0.99758 | 2 |
| 2 | -7.52 | 3.03500 | 4 | -7.87 | 2.01514 | 4 | -6.49 | 1.4795 | NA | NA | NA | 18.32695 | 0.99499 | 3 |
| 2 | -0.06 | 0.04155 | 4 | -0.13 | 0.03456 | NA | NA | NA | NA | NA | NA | 0.35082 | 0.99435 | 2 |

### References to primary studies included in the review:

The list of references below provides the primary reference only for the studies included in the review. Studies marked + were included in the NMA. Studies marked with * were also included in the control group moderation analyses.

| ^*+^ | Ahlen J *et al*. Universal Prevention for Anxiety and Depressive Symptoms in Children: A Meta-analysis of Randomized and Cluster-Randomized Trials. The journal of primary prevention 2015; 36(6): 387-403. |
| --- | --- |
|  | Anticich S *et al*. Prevention of childhood anxiety and promotion of resilience among preschool-aged children: A universal school-based trial. Advances in school mental health promotion 2013; 6(2): 93-121. |
|  | Araya R, Fritsch R, Spears M, et al. School intervention to improve mental health of students in Santiago, Chile: a randomized clinical trial. Jama, Pediatr 2013; 167(11): 1004-10. |
|  | Arnarson EO, Craighead WE. Prevention of depression among Icelandic adolescents. Behav Res Ther 2009; 47(7): 577-85. |
| ^*+^ | Attwood M, Meadows S, Stallard P, Richardson T. Universal and targeted computerised cognitive behavioural therapy (Think, Feel, Do) for emotional health in schools: Results from two exploratory studies. Child and adolescent mental health 2012; 17(3): 173-8. |
| ^*+^ | Aune T, Stiles TC. Universal-based prevention of syndromal and subsyndromal social anxiety: A randomized controlled study. J Consult Clin Psychol 2009; 77(5): 867-79. |
| ^*+^ | Baker SB, Butler JN. Effects of preventive cognitive self-instruction training on adolescent attitudes, experiences, and state anxiety. J Prim Prev 1984; 5(1): 17-26. |
| ^*+^ | Balle M, Tortella-Feliu M. Efficacy of a brief school-based program for selective prevention of childhood anxiety. Anxiety stress coping 2010; 23(1): 71-85. |
|  | Barrett P, Lock S, Farrell L. Developmental differences in universal preventive intervention for child anxiety. Clinical child psychology and psychiatry 2005; 10(4): 539-55. |
| ^*+^ | Barrett P, Turner C. Prevention of anxiety symptoms in primary school children: preliminary results from a universal school-based trial. Br J Clin Psychol 2001; 40(Pt 4): 399-410. |
| ^*+^ | Barry M, Murphy M, O'Donovan H. Assessing the effectiveness of a cognitive behavioural group coaching intervention in reducing symptoms of depression among adolescent males in a school setting. International Coaching Psychology Review 2017; 12(2): 101-9. |
| ^*+^ | Berry K, Hunt CJ. Evaluation of an intervention program for anxious adolescent boys who are bullied at school. J Adolesc Health 2009; 45(4): 376-82. |
|  | Bonhauser M, Fernandez G, Puschel K, et al. Improving physical fitness and emotional well-being in adolescents of low socioeconomic status in Chile: results of a school-based controlled trial. Health Promot Internation 2005; 20(2): 113-22. |
| ^*+^ | Bouchard S, Gervais J, Gagnier N, Loranger C. Evaluation of a primary prevention program for anxiety disorders using story books with children aged 9-12 years. J Prim Prev 2013; 34(5): 345-58. |
|  | Britton Willoughby BLN, E. Niles Halsey, F. Rocha, Tomas Fisher Nathan, E. Gold Jonathan, S. A randomized controlled pilot trial of classroom-based mindfulness meditation compared to an active control condition in sixth-grade children. J Sch Psychol 2014; 52(3): 263-78. |
| ^+^ | Burckhardt R, Manicavasagar V, Batterham PJ, Hadzi-Pavlovic D. A randomized controlled trial of strong minds: A school-based mental health program combining acceptance and commitment therapy and positive psychology. J Sch Psychol 2016; 57: 41-52. |
|  | Burckhardt R, Manicavasagar V, Batterham PJ, Miller LM, Talbot E, Lum A. A Web-Based Adolescent Positive Psychology Program in Schools: Randomized Controlled Trial. J Med Internet Res 2015; 17(7): e187. |
| ^*+^ | Calear AL, Batterham PJ, Poyser CT, Mackinnon AJ, Griffiths KM, Christensen H. Cluster randomised controlled trial of the e-couch Anxiety and Worry program in schools. J Affect Disord 2016; 196: 210-7. |
| ^*+^ | Calear AL, Christensen H, Brewer J, Mackinnon A, Griffiths KM. A pilot randomized controlled trial of the e-couch anxiety and worry program in schools. Internet Interventions 2016; 6: 1-5. |
| ^*+^ | Calear AL, Christensen H, Mackinnon A, Griffiths KM, O'Kearney R. The YouthMood Project: a cluster randomized controlled trial of an online cognitive behavioral program with adolescents. J Consult Clin Psychol 2009; 77(6): 1021-32. |
| ^*+^ | Cardemil EV, Reivich KJ, Beevers CG, Seligman ME, James J. The prevention of depressive symptoms in low-income, minority children: two-year follow-up. Behav Res Ther 2007; 45(2): 313-27. |
| ^*+^ | Chaplin TM, Gillham JE, Reivich K, et al. Depression Prevention for Early Adolescent Girls: A Pilot Study of All Girls Versus Co-Ed Groups. J Early Adolesc 2006; 26(1): 110-26. |
| ^*+^ | Clarke GN, Hawkins W, Murphy M, Sheeber LB, Lewinsohn PM, Seeley JR. Targeted prevention of unipolar depressive disorder in an at-risk sample of high school adolescents: a randomized trial of a group cognitive intervention. J Am Acad Child Adolesc Psychiatry 1995; 34(3): 312-21. |
| ^*+^ | Clarke Gregory NH, Wesley Murphy, Mary Sheeber, Lisa. School-based primary prevention of depressive symptomatology in adolescents: Findings from two studies. Journal of adolescent research 1993(A); 8(2): 183-204. |
| ^+^ | Clarke Gregory NH, Wesley Murphy, Mary Sheeber, Lisa. School-based primary prevention of depressive symptomatology in adolescents: Findings from two studies. Journal of adolescent research 1993(B); 8(2): 183-204. |
| ^*+^ | Collins SWL, Marks Durkin, Kevin. Effects on coping skills and anxiety of a universal school-based mental health intervention delivered in Scottish primary schools. School Psychology International 2014; 35(1): 85-100. |
| ^*+^ | Congleton, Baker A. The effect of a cognitive-behavioral group intervention on the locus of control, attributional style, and depressive symptoms of middle school students; 2019. Thesis. University of Kentucky, USA. |
| ^*+^ | Cooley-Strickland MR, Griffin RS, Darney D, Otte K, Ko J. Urban African American youth exposed to community violence: a school-based anxiety preventive intervention efficacy study. J 2011; 39(2): 149-66. |
| ^*+^ | Cova FR, Paulina Melipillan, Roberto. Evaluation of the efficacy of a prevention program for depression in female adolescents. Terapia psicologica 2011; 29(2): 245-50. |
|  | Cowell JM, McNaughton D, Ailey S, Gross D, Fogg L. Clinical Trail Outcomes of the Mexican American Problem Solving Program (MAPS). Hispanic Health Care International : The Official Journal of The National Association of Hispanic Nurses 2009; 7(4): 179-89. |
| ^*+^ | Dobson Keith SHJ, Ahnberg Fata, Ladan Scherrer, Martin Allan Lauren, C. The prevention of depression and anxiety in a sample of high-risk adolescents: A randomized controlled trial. Canadian journal of school psychology 2010; 25(4): 291-310. |
|  | Eather NMP, J. Lubans David, R. Effects of exercise on mental health outcomes in adolescents: Findings from the CrossFitTM teens RCT. Psychology of sport & exercise 2016; 26: 14-23. |
| ^*+^ | Essau CA, Conradt J, Sasagawa S, Ollendick TH. Prevention of anxiety symptoms in children: results from a universal school-based trial. Behav 2012; 43(2): 450-64. |
| ^+^ | Fitzgerald A, Rawdon C, Dooley B. A randomized controlled trial of attention bias modification training for socially anxious adolescents. Behav Res Ther 2016; 84: 1-8.. |
|  | Fung J, Guo S, Jin J, Bear L, Lau A. A pilot randomized trial evaluating a school-based mindfulness intervention for ethnic minority youth. Mindfulness 2016; 7(4): 819-28. |
|  | Gaete J, Martinez V, Fritsch R, Rojas G, Montgomery AA, Araya R. Indicated school-based intervention to improve depressive symptoms among at risk Chilean adolescents: a randomized controlled trial. BMC Psychiatry 2016; 16: 276. |
| ^*+^ | Gallegos, J. (2008). Preventing childhood anxiety and depression: Testing the effectiveness of a school-based program in Mexico (Order No. 3341564). Available from ProQuest Dissertations & Theses Global. (304487266). |
| ^*+^ | Gillham Jane E. Preventing depressive symptoms in school children. Dissertation abstracts international: section b: the sciences and engineering 1995; 55(9-B): 4119. |
| ^*+^ | Gillham Jane ERK, J. Freres Derek, R. Lascher, Marisa Litzinger, Samantha Shatte, Andrew Seligman Martin, E. P. School-based prevention of depression and anxiety symptoms in early adolescence: A pilot of a parent intervention component. School psychology quarterly 2006; 21(3): 323-48. |
| ^*+^ | Gillham JE, Reivich KJ, Brunwasser SM, et al. Evaluation of a group cognitive-behavioral depression prevention program for young adolescents: a randomized effectiveness trial. J Clin Child Adolesc Psychol 2012; 41(5): 621-39. |
| ^*+^ | Gillham JE, Reivich KJ, Freres DR, et al. School-based prevention of depressive symptoms: A randomized controlled study of the effectiveness and specificity of the Penn Resiliency Program. J Consult Clin Psychol 2007; 75(1): 9-19. |
| ^+^ | Gucht K, Griffith JW, Hellemans R, Bockstaele M, Pascal-Claes F, Raes F. Acceptance and Commitment Therapy (ACT) for adolescents: Outcomes of a large-sample, school-based, cluster-randomized controlled trial. Mindfulness 2017; 8(2): 408-16. |
|  | Haden SC, Daly L, Hagins M. A randomised controlled trial comparing the impact of yoga and physical education on the emotional and behavioural functioning of middle school children. Focus on alternative and complementary therapies 2014; 19(3): 148-55. |
| ^+^ | Hiebert BK, Boelle Jaknavorian, Armine. School-based relaxation: Attempting primary prevention. Canadian journal of counselling 1989; 23(3): 273-87. (Hiebert 1989a and Hiebert 1989b) |
| ^*+^ | Hodas R. An investigation of the relationship between positive and negative mental health factors and academic performance among early adolescent girls. Dissertation abstracts international: section b: the sciences and engineering 2016; 76(12-B(E)): |
| ^*+^ | Horowitz JL, Garber J, Ciesla JA, Young JF, Mufson L. Prevention of depressive symptoms in adolescents: a randomized trial of cognitive-behavioral and interpersonal prevention programs. J Consult Clin Psychol 2007; 75(5): 693-706. |
|  | Hunt C, Andrews G, Crino R, Erskine A, Sakashita C. Randomized controlled trial of an early intervention programme for adolescent anxiety disorders. Aust N Z J Psychiatry 2009; 43(4): 300-4. |
| ^*+^ | Jaycox LH, Reivich KJ, Gillham J, Seligman ME. Prevention of depressive symptoms in school children. Behav Res Ther 1994; 32(8): 801-16. |
| ^+^ | Johnson C, Burke C, Brinkman S, Wade T. A randomized controlled evaluation of a secondary school mindfulness program for early adolescents: Do we have the recipe right yet? Behav Res Ther 2017; 99: 37-46. |
| ^+^ | Johnson C, Burke C, Brinkman S, Wade T. Effectiveness of a school-based mindfulness program for transdiagnostic prevention in young adolescents. Behav Res Ther 2016; 81: 1-11. |
| ^*+^ | Johnstone J, Rooney RM, Hassan S, Kane RT. Prevention of depression and anxiety symptoms in adolescents: 42 and 54 months follow-up of the Aussie Optimism Program-Positive Thinking Skills. Front Psychol 2014; 5: 364. |
| ^*+^ | Jordans MJ, Komproe IH, Tol WA, et al. Evaluation of a classroom-based psychosocial intervention in conflict-affected Nepal: a cluster randomized controlled trial. J Child Psychol Psychiatry 2010; 51(7): 818-26. |
| ^+^ | Khalsa SB, Hickey-Schultz L, Cohen D, Steiner N, Cope S. Evaluation of the mental health benefits of yoga in a secondary school: a preliminary randomized controlled trial. J Behav Health Serv Res 2012; 39(1): 80-90. |
| ^*+^ | Kindt KC, Kleinjan M, Janssens JM, Scholte RH. Evaluation of a school-based depression prevention program among adolescents from low-income areas: a randomized controlled effectiveness trial. Int J Environ Res Public Health 2014; 11(5): 5273-93. |
| ^*+^ | Kiselica Mark SBS, B. Thomas Ronald, N. Reedy, Susan. Effects of stress inoculation training on anxiety, stress, and academic performance among adolescents. J Couns Psychol 1994; 41(3): 335-42. |
|  | Liddle I, Macmillan S. Evaluating the FRIENDS programme in a Scottish setting. Educational Psychology in Practice 2010; 26(1): 53-67. |
| ^+^ | Livheim FH, Louise Ghaderi, Ata Magnusdottir, Thora Hogfeldt, Anna Rowse, Julie Turner, Simone Hayes Steven, C. Tengstrom, Anders. The effectiveness of Acceptance and Commitment Therapy for adolescent mental health: Swedish and Australian pilot outcomes. Journal of child and family studies 2015; 24(4): 1016-30. |
| ^*+^ | Lock S, Barrett PM. A longitudinal study of developmental differences in universal preventive intervention for child anxiety. Behaviour Change 2003; 20(4): 183-99. |
| ^*+^ | Lowry-Webster Hayley MBP, M. Dadds Mark, R. A universal prevention trial of anxiety and depressive symptomatology in childhood: Preliminary data from an Australian study. Behaviour change 2001; 18: 36-50. |
| ^*+^ | Manassis K, Wilansky-Traynor P, Farzan N, Kleiman V, Parker K, Sanford M. The feelings club: randomized controlled evaluation of school-based CBT for anxious or depressive symptoms. Depress Anxiety 2010; 27(10): 945-52. |
| ^*+^ | McCarty CA, Violette HD, Duong MT, Cruz RA, McCauley E. A randomized trial of the Positive Thoughts and Action program for depression among early adolescents. J Clin Child Adolesc Psychol 2013; 42(4): 554-63. |
| ^*+^ | McCarty CA, Violette HD, McCauley E. Feasibility of the positive thoughts and actions prevention program for middle schoolers at risk for depression. Depress Res Treat 2011; 2011: 241386. |
|  | McLaughlin C. Evaluating the effect of an empirically-supported group intervention for students at-risk for depression in a rural school district [thesis]. Dissertation abstracts international: section b: the sciences and engineering 2011; 71(9-b): 5820. |
| ^*+^ | McLoone Jordana KRR, M. Comparison of an anxiety management program for children implemented at home and school: Lessons learned. Sch 2012; 4(4): 231-42. |
|  | Mendelson T, Greenberg MT, Dariotis JK, Gould LF, Rhoades BL, Leaf PJ. Feasibility and preliminary outcomes of a school-based mindfulness intervention for urban youth. J Abnorm Child Psychol 2010; 38(7): 985-94. |
| ^+^ | Merry S, McDowell H, Wild CJ, Bir J, Cunliffe R. A randomized placebo-controlled trial of a school-based depression prevention program. J Am Acad Child Adolesc Psychiatry 2004; 43(5): 538-47. |
| ^*+^ | Mifsud C, Rapee RM. Early intervention for childhood anxiety in a school setting: outcomes for an economically disadvantaged population. J Am Acad Child Adolesc Psychiatry 2005; 44(10): 996-1004. |
| ^*+^ | Miller LD, Laye-Gindhu A, Bennett JL, et al. An effectiveness study of a culturally enriched school-based CBT anxiety prevention program. J Clin Child Adolesc Psychol 2011; 40(4): 618-29. |
| ^*+^ | Miller LD, Laye-Gindhu A, Liu Y, March JS, Thordarson DS, Garland EJ. Evaluation of a preventive intervention for child anxiety in two randomized attention-control school trials. Behav Res Ther 2011; 49(5): 315-23. |
| ^*+^ | Miller LD, Laye-Gindhu A, Liu Y, March JS, Thordarson DS, Garland EJ. Evaluation of a preventive intervention for child anxiety in two randomized attention-control school trials. Behav Res Ther 2011; 49(5): 315-23. |
| ^*+^ | Miller Lynn DS, Christina Garland, E. Jane Clark, Sandra. The ABCs of CBT (cognitive behavior therapy): Evidence-based approaches to child anxiety in public school settings. Journal of counseling and development 2010; 88(4): 432-9. |
|  | Noel La TR, Kathryn Gromer, Jill. Depression prevention among rural preadolescent girls: A randomized controlled trial. School social work journal 2013; 38(1): 1-18. |
|  | Owen HL, Wayne. The effects of three treatment methods upon anxiety and inappropriate attentional style among high school athletes. International journal of sport psychology 1982; 13(3): 154-62. |
|  | Pahl Kristine MBP, M. Preventing anxiety and promoting social and emotional strength in preschool children: A universal evaluation of the Fun FRIENDS program. Advances in school mental health promotion 2010; 3(3): 14-25. |
| ^*+^ | Pattison C, Lynd-Stevenson R. The prevention of depressive symptoms in children: The immediate and long-term outcomes of a school-based program. Behaviour change 2001; 18(2): 92-102. |
| ^*+^ | Perry Y, Werner-Seidler A, Calear A, et al. Preventing Depression in Final Year Secondary Students: School-Based Randomized Controlled Trial. J Med Internet Res 2017; 19(11): e369. |
| ^*+^ | Pophillat E, Rooney RM, Nesa M, et al. Preventing Internalizing Problems in 6-8 Year Old Children: A Universal School-Based Program. Front Psychol 2016; 7: 1928. |
| ^*+^ | Poppelaars M, Tak YR, Lichtwarck-Aschoff A, et al. A randomized controlled trial comparing two cognitive-behavioral programs for adolescent girls with subclinical depression: A school-based program (Op Volle Kracht) and a computerized program (SPARX). Behav Res Ther 2016; 80: 33-42. |
| ^*+^ | Possel P, Adelson JL, Hautzinger M. A randomized trial to evaluate the course of effects of a program to prevent adolescent depressive symptoms over 12 months. Behav Res Ther 2011; 49(12): 838-51. |
| ^*+^ | Possel P, Horn AB, Groen G, Hautzinger M. School-based prevention of depressive symptoms in adolescents: a 6-month follow-up. J Am Acad Child Adolesc Psychiatry 2004; 43(8): 1003-10. |
| ^*+^ | Possel P, Martin NC, Garber J, Hautzinger M. A randomized controlled trial of a cognitive-behavioral program for the prevention of depression in adolescents compared with nonspecific and no-intervention control conditions. J Couns Psychol 2013; 60(3): 432-8. |
| ^+^ | Potek R. Mindfulness as a school-based prevention program and its effect on adolescent stress, anxiety and emotion regulation. Dissertation abstracts international: section b: the sciences and engineering 2012; 73(5-B): 3272. |
| ^*+^ | Puskar K, Sereika S, Tusaie-Mumford K. Effect of the Teaching Kids to Cope (TKC) program on outcomes of depression and coping among rural adolescents. J Child Adolesc Psychiatr Nurs 2003;: 71-80. |
| ^*+^ | Quayle DD, Suzanne Roberts, Claire Kane, Robert Ebsworthy, Greg. The effect of an optimism and lifeskills program on depressive symptoms in preadolescence. Behaviour change 2001; 18(4): 194-203. |
| ^+^ | Raes FGJ, W. Van der Gucht, Katleen Williams, J. Mark G. School-based prevention and reduction of depression in adolescents: A cluster-randomized controlled trial of a mindfulness group program. Mindfulness 2014; 5(5):477-86. |
| ^*+^ | Rice Cristy L. Reducing anxiety in middle school and high school students: A comparison of cognitive-behavioral therapy and relaxation training approaches. Dissertation abstracts international section a: humanities and social sciences 2009; 69(7-A): 2607. |
| ^+^ | Rivet-Duval EH, Sandra Hunt, Caroline. Preventing adolescent depression in Mauritius: A universal school-based program. Child and adolescent mental health 2011; 16(2): 86-91. |
| ^*+^ | Roberts C, Kane R, Thomson H, Bishop B, Hart B. The prevention of depressive symptoms in rural school children: a randomized controlled trial. J Consult Clin Psychol 2003; 71(3): 622-8. |
| ^*+^ | Roberts CM, Kane R, Bishop B, Cross D, Fenton J, Hart B. The prevention of anxiety and depression in children from disadvantaged schools. Behav Res Ther 2010; 48(1): 68-73. |
|  | Roberts CM, Kane RT, Rooney RM, et al. Efficacy of the Aussie Optimism Program: Promoting Pro-social Behavior and Preventing Suicidality in Primary School Students. A Randomised-Controlled Trial. Front Psychol 2018; 8(1392). |
| ^*+^ | Rodgers AD, Sandra. A controlled evaluation of the 'FRIENDS for life' emotional resiliency programme on overall anxiety levels, anxiety subtype levels and school adjustment. Child and adolescent mental health 2015; 20(1): 13-9. |
| ^*+^ | Rohde P, Stice E, Shaw H, Briere FN. Indicated cognitive behavioral group depression prevention compared to bibliotherapy and brochure control: acute effects of an effectiveness trial with adolescents. J Consult Clin Psychol 2014; 82(1): 65-74. |
| ^*+^ | Rooney RR, Clare Kane, Robert Pike, Lisbeth Winsor, Amber White, Julia Brown, Annette. The Prevention of Depression in 8- to 9-Year-Old Children: A Pilot Study. Australian journal of guidance and counselling 2006; 16(1): 76-90. |
| ^+^ | Rose K, Hawes DJ, Hunt CJ. Randomized controlled trial of a friendship skills intervention on adolescent depressive symptoms. J Consult Clin Psychol 2014; 82(3): 510-20. |
| ^*+^ | Ruttledge RD, Eileen Greene, Gabrielle Mullany, Mary Charles, Elizabeth Frehill, Joanne Moriarty, Maura. A randomised controlled trial of the FRIENDSfor Life emotional resilience programme delivered by teachers in Irish primary schools. Educational and child psychology 2016; 33(2): 69-89. |
|  | Sawyer MG, Pfeiffer S, Spence SH, et al. School-based prevention of depression: a randomised controlled study of the beyondblue schools research initiative. J Child Psychol Psychiatry 2010; 51(2): 199-209. |
| ^+^ | Scholten H, Malmberg M, Lobel A, Engels RC, Granic I. A Randomized Controlled Trial to Test the Effectiveness of an Immersive 3D Video Game for Anxiety Prevention among Adolescents. PLoS ONE 2016; 11(1): e0147763. |
| ^+^ | Schoneveld EA, Lichtwarck-Aschoff A, Granic I. Preventing Childhood Anxiety Disorders: Is an Applied Game as Effective as a Cognitive Behavioral Therapy-Based Program? Prev Sci 2018; 19(2): 220-32. |
| ^+^ | Schoneveld EA, Malmberg M, Lichtwarck-Aschoff A, Verheijen GP, Engels RC, Granic I. A neurofeedback video game (MindLight) to prevent anxiety in children: A randomized controlled trial. Computers in Human Behavior 2016; 63: 321-33. |
| ^*+^ | Shatte Andrew J. Prevention of depressive symptoms in adolescents: Issues of dissemination and mechanisms of change. Dissertation abstracts international: section b: the sciences and engineering 1997; 57(11-B): 7236. |
| ^*+^ | Sheffield JK, Spence SH, Rapee RM, et al. Evaluation of universal, indicated, and combined cognitive-behavioral approaches to the prevention of depression among adolescents. J Consult Clin Psychol 2006; 74(1): 66-79. (Sheffield 2006a and Sheffield 2006b). |
| ^*+^ | Simpson Anna T. The roles of self-regulation and coping in a preventative cognitive-behavioural intervention for school-age children at-risk for internalizing disorders. Dissertation abstracts international: section b: the sciences and engineering 2008; 69(6-B): 3862. |
| ^*+^ | Siu Fung Ying A. Internalizing problems among primary school children in Hong Kong: Prevalence and treatment. Dissertation abstracts international section a: humanities and social sciences 2008; 69(1-A): 115. |
| ^*+^ | Soffer, A. G. (2003). School -based social skills training to reduce children's depressive symptomatology (Order No. 3074683). Available from ProQuest Dissertations & Theses Global. (305330756). |
| ^*+^ | Spence SH, Sheffield JK, Donovan CL. Preventing adolescent depression: An evaluation of the problem solving for life program. Journal of Consulting and Clinical Psychology 2003; 71(1): 3-13. |
| ^*+^ | Sportel BE, de Hullu E, de Jong PJ, Nauta MH. Cognitive bias modification versus CBT in reducing adolescent social anxiety: a randomized controlled trial. PLoS ONE 2013; 8(5): e64355. |
|  | Stallard P, Phillips R, Montgomery AA, et al. A cluster randomised controlled trial to determine the clinical effectiveness and cost-effectiveness of classroom-based cognitive-behavioural therapy (CBT) in reducing symptoms of depression in high-risk adolescents. Health Technol Assess 2013; 17(47): vii-xvii, 1-109. (Stallard 2012a and Stallard 2012b) |
|  | Stallard P, Skryabina E, Taylor G, et al. Classroom-based cognitive behaviour therapy (FRIENDS): a cluster randomised controlled trial to Prevent Anxiety in Children through Education in Schools (PACES). Lancet Psychiatry 2014; 1(3): 185-92. |
| ^*+^ | Stice E, Rohde P, Seeley JR, Gau JM. Brief cognitive-behavioral depression prevention program for high-risk adolescents outperforms two alternative interventions: a randomized efficacy trial. J Consult Clin Psychol 2008; 76(4): 595-606. |
|  | Stoppelbein Laura A. Primary prevention: An evaluation of a high-school based cognitive-behavioral program. Dissertation abstracts international: section b: the sciences and engineering 2004; 64(8-B): 4066. |
| ^*+^ | Tak YR, Lichtwarck-Aschoff A, Gillham JE, Van Zundert RM, Engels RC. Universal School-Based Depression Prevention 'Op Volle Kracht': a Longitudinal Cluster Randomized Controlled Trial. J Abnorm Child Psychol 2016; 44(5): 949-61. |
| ^+^ | Tokolahi E, Vandal AC, Kersten P, Pearson J, Hocking C. Cluster-randomised controlled trial of an occupational therapy intervention for children aged 11–13 years, designed to increase participation to prevent symptoms of mental illness. Child and Adolescent Mental Health 2018; 23(4): 313-27. |
| ^*+^ | Tomba E, Belaise C, Ottolini F, et al. Differential effects of well-being promoting and anxiety-management strategies in a non-clinical school setting. J Anxiety Disord 2010; 24(3): 326-33. |
| ^*+^ | Topper M, Emmelkamp PM, Watkins E, Ehring T. Prevention of anxiety disorders and depression by targeting excessive worry and rumination in adolescents and young adults: A randomized controlled trial. Behav Res Ther 2017; 90: 123-36. |
| ^*+^ | van Starrenburg ML, Kuijpers RC, Kleinjan M, Hutschemaekers GJ, Engels RC. Effectiveness of a Cognitive Behavioral Therapy-Based Indicated Prevention Program for Children with Elevated Anxiety Levels: a Randomized Controlled Trial. Prev Sci 2017; 18(1): 31-9. |
|  | Velasquez Ana MLM, Adelaida Quinonez, Natalia Paba Diana, Patricia. Yoga for the prevention of depression, anxiety, and aggression and the promotion of socio-emotional competencies in school-aged children. Educational research and evaluation 2015; 21(5-6): 407-21. |
| ^*+^ | Wijnhoven LA, Creemers DH, Vermulst AA, Scholte RH, Engels RC. Randomized controlled trial testing the effectiveness of a depression prevention program ('Op Volle Kracht') among adolescent girls with elevated depressive symptoms. J Abnorm Child Psychol 2014; 42(2): 217-28. |
| ^*+^ | Wong N, Kady L, Mewton L, Sunderland M, Andrews G. Preventing anxiety and depression in adolescents: A randomised controlled trial of two school-based Internet-delivered cognitive behavioural therapy programmes. Internet interventions 2014; 1(2): 90-4. |
| ^*+^ | Woods BJP, E. Effectiveness of a school-based indicated early intervention program for Maori and Pacific adolescents. Journal of pacific rim psychology 2011; 5(1): 40-50. |
| ^+^ | Young JF, Benas JS, Schueler CM, Gallop R, Gillham JE, Mufson L. A Randomized Depression Prevention Trial Comparing Interpersonal Psychotherapy--Adolescent Skills Training to Group Counseling in Schools. Prev Sci 2016; 17(3): 314-24. |
| ^+^ | Young JF, Mufson L, Davies M. Efficacy of Interpersonal Psychotherapy-Adolescent Skills Training: an indicated preventive intervention for depression. J Child Psychol Psychiatry 2006; 47(12): 1254-62. |
| ^+^ | Young JF, Mufson L, Gallop R. Preventing depression: a randomized trial of interpersonal psychotherapy-adolescent skills training. Depress Anxiety 2010; 27(5): 426-33. |
|  | Yu L. Preventing depressive symptoms in Chinese children. Dissertation abstracts international: section b: the sciences and engineering 2000; 60(12-B): 6389. |
| ^*+^ | Ab Ghaffar SF, Mohd Sidik S, Ibrahim N, Awang H, Gyanchand Rampal LR. Effect of a School-Based Anxiety Prevention Program among Primary School Children. Int J Environ Res Public Health. 2019 Dec 5;16(24):4913. doi: 10.3390/ijerph16244913. PMID: 31817328; PMCID: PMC6950005. |
| ^+^ | Bazzano AN, Sun Y, Chavez-Gray V, Akintimehin T, Gustat J, Barrera D, Roi C. Effect of Yoga and Mindfulness Intervention on Symptoms of Anxiety and Depression in Young Adolescents Attending Middle School: A Pragmatic Community-Based Cluster Randomized Controlled Trial in a Racially Diverse Urban Setting. Int J Environ Res Public Health. 2022 Sep 24;19(19):12076. doi: 10.3390/ijerph191912076. PMID: 36231378; PMCID: PMC9564597. |
| ^*+^ | Brière, F.N., Reigner, A., Yale-Soulière, G. et al. Effectiveness Trial of Brief Indicated Cognitive-Behavioral Group Depression Prevention in French-Canadian Secondary Schools. School Mental Health 11, 728–740 (2019). https://doi.org/10.1007/s12310-019-09316-2 |
| ^+^ | Calvete E, Fernández-Gonzalez L, Orue I, Echezarraga A, Royuela-Colomer E, Cortazar N, Muga J, Longa M, Yeager DS. The Effect of an Intervention Teaching Adolescents that People can Change on Depressive Symptoms, Cognitive Schemas, and Hypothalamic-Pituitary-Adrenal Axis Hormones. J Abnorm Child Psychol. 2019 Sep;47(9):1533-1546. doi: 10.1007/s10802-019-00538-1. PMID: 30903540; PMCID: PMC6650351. |
|  | Chen J, Johnstone KM, Kemps E. A randomised controlled trial evaluating two universal prevention programs for children: Building resilience to manage worry. J Affect Disord. 2022 Jan 15;297:437-446. doi: 10.1016/j.jad.2021.10.079. Epub 2021 Oct 29. PMID: 34715158. |
|  | David OA, Cardoș RAI, Matu S. Is REThink therapeutic game effective in preventing emotional disorders in children and adolescents? Outcomes of a randomized clinical trial. Eur Child Adolesc Psychiatry. 2019 Jan;28(1):111-122. doi: 10.1007/s00787-018-1192-2. Epub 2018 Jul 10. PMID: 29992353. |
| ^*+^ | de Jonge-Heesen KWJ, Rasing SPA, Vermulst AA, Scholte RHJ, van Ettekoven KM, Engels RCME, Creemers DHM. Randomized control trial testing the effectiveness of implemented depression prevention in high-risk adolescents. BMC Med. 2020 Jul 24;18(1):188. doi: 10.1186/s12916-020-01656-0. PMID: 32703288; PMCID: PMC7379355. |
|  | Fernández-Martínez I, Orgilés M, Morales A, Espada JP, Essau CA. One-Year follow-up effects of a cognitive behavior therapy-based transdiagnostic program for emotional problems in young children: A school-based cluster-randomized controlled trial. J Affect Disord. 2020 Feb 1;262:258-266. doi: 10.1016/j.jad.2019.11.002. Epub 2019 Nov 4. PMID: 31733917 |
| ^+^ | Garaigordobil M, Jaureguizar J, Bernarás E. Evaluation of the effects of a childhood depression prevention program. J Psychol. 2019;153(2):127-140. doi: 10.1080/00223980.2018.1502741. Epub 2018 Oct 30. PMID: 30376644. |
|  | García-Escalera J, Valiente RM, Sandín B, Ehrenreich-May J, Prieto A, Chorot P. The Unified Protocol for Transdiagnostic Treatment of Emotional Disorders in Adolescents (UP-A) Adapted as a School-Based Anxiety and Depression Prevention Program: An Initial Cluster Randomized Wait-List-Controlled Trial. Behav Ther. 2020 May;51(3):461-473. doi: 10.1016/j.beth.2019.08.003. Epub 2019 Aug 14. PMID: 32402261. |
| ^*+^ | Hamdani SU, Huma ZE, Tamizuddin-Nizami A, Baneen UU, Suleman N, Javed H, Malik A, Wang D, Mazhar S, Khan SA, Minhas FA, Rahman A. Feasibility and acceptability of a multicomponent, group psychological intervention for adolescents with psychosocial distress in public schools of Pakistan: a feasibility cluster randomized controlled trial (cRCT). Child Adolesc Psychiatry Ment Health. 2022 Jun 21;16(1):47. doi: 10.1186/s13034-022-00480-z. PMID: 35729589; PMCID: PMC9210054. |
|  | Harris N, Warbrick I, Fleming T, Borotkanics R, Atkins D, Lubans D. Impact of high-intensity interval training including Indigenous narratives on adolescents' mental health: a cluster-randomised controlled trial. Aust N Z J Public Health. 2022 Dec;46(6):794-799. doi: 10.1111/1753-6405.13312. Epub 2022 Oct 19. PMID: 36259747 |
| ^*+^ | Haugland BSM, Haaland ÅT, Baste V, Bjaastad JF, Hoffart A, Rapee RM, Raknes S, Himle JA, Husabø E, Wergeland GJ. Effectiveness of Brief and Standard School-Based Cognitive-Behavioral Interventions for Adolescents With Anxiety: A Randomized Noninferiority Study. J Am Acad Child Adolesc Psychiatry. 2020 Apr;59(4):552-564.e2. doi: 10.1016/j.jaac.2019.12.003. Epub 2020 Jan 8. PMID: 31926224. |
|  | Humphrey N, Panayiotou M. Bounce Back: randomised trial of a brief, school-based group intervention for children with emergent mental health difficulties. Eur Child Adolesc Psychiatry. 2022 Jan;31(1):205-210. doi: 10.1007/s00787-020-01612-6. Epub 2020 Aug 8. PMID: 32770409. |
|  | Johnstone KM, Middleton T, Kemps E, Chen J. A pilot investigation of universal school-based prevention programs for anxiety and depression symptomology in children: A randomized controlled trial. J Clin Psychol. 2020 Jul;76(7):1193-1216. doi: 10.1002/jclp.22926. Epub 2020 Jan 13. PMID: 31943189. |
| ^+^ | Khanna, P., Singh, K. Do All Positive Psychology Exercises Work for Everyone? Replication of Seligman et al.’s (2005) Interventions among Adolescents. Psychol Stud 64, 1–10 (2019). https://doi.org/10.1007/s12646-019-00477-3 |
|  | Klim-Conforti P, Zaheer R, Levitt AJ, Cheung AH, Schachar R, Schaffer A, Goldstein BI, Fefergrad M, Niederkrotenthaler T, Sinyor M. The Impact of a Harry Potter-Based Cognitive-Behavioral Therapy Skills Curriculum on Suicidality and Well-being in Middle Schoolers: A Randomized Controlled Trial. J Affect Disord. 2021 May 1;286:134-141. doi: 10.1016/j.jad.2021.02.028. Epub 2021 Feb 10. PMID: 33721740. |
| ^+^ | Kuyken W, Ball S, Crane C, Ganguli P, Jones B, Montero-Marin J, Nuthall E, Raja A, Taylor L, Tudor K, Viner RM, Allwood M, Aukland L, Dunning D, Casey T, Dalrymple N, De Wilde K, Farley ER, Harper J, Kappelmann N, Kempnich M, Lord L, Medlicott E, Palmer L, Petit A, Philips A, Pryor-Nitsch I, Radley L, Sonley A, Shackleford J, Tickell A, Blakemore SJ, Team TM, Ukoumunne OC, Greenberg MT, Ford T, Dalgleish T, Byford S, Williams JMG. Effectiveness and cost-effectiveness of universal school-based mindfulness training compared with normal school provision in reducing risk of mental health problems and promoting well-being in adolescence: the MYRIAD cluster randomised controlled trial. Evid Based Ment Health. 2022 Jul 12;25(3):99–109. doi: 10.1136/ebmental-2021-300396. Epub ahead of print. PMID: 35820992; PMCID: PMC9340028. |
| ^+^ | Lima RA, de Barros MVG, Bezerra J, Dos Santos SJ, Monducci E, Rodriguez-Ayllon M, Soares FC. Universal school-based intervention targeting depressive symptoms in adolescents: A cluster randomized trial. Scand J Med Sci Sports. 2022 Mar;32(3):622-631. doi: 10.1111/sms.14115. Epub 2021 Dec 23. PMID: 34923679. |
| ^*+^ | Maalouf FT, Alrojolah L, Ghandour L, Afifi R, Dirani LA, Barrett P, Nakkash R, Shamseddeen W, Tabaja F, Yuen CM, Becker AE. Building Emotional Resilience in Youth in Lebanon: a School-Based Randomized Controlled Trial of the FRIENDS Intervention. Prev Sci. 2020 Jul;21(5):650-660. doi: 10.1007/s11121-020-01123-5. PMID: 32363411. |
|  | Makover H, Adrian M, Wilks C, Read K, Stoep AV, McCauley E. Indicated Prevention for Depression at the Transition to High School: Outcomes for Depression and Anxiety. Prev Sci. 2019 May;20(4):499-509. doi: 10.1007/s11121-019-01005-5. PMID: 30852711. |
| ^*+^ | Martinsen KD, Rasmussen LMP, Wentzel-Larsen T, Holen S, Sund AM, Løvaas MES, Patras J, Kendall PC, Waaktaar T, Neumer SP. Prevention of anxiety and depression in school children: Effectiveness of the transdiagnostic EMOTION program. J Consult Clin Psychol. 2019 Feb;87(2):212-219. doi: 10.1037/ccp0000360. Epub 2018 Dec 13. PMID: 30550301. |
|  | Michelson D, Malik K, Parikh R, Weiss HA, Doyle AM, Bhat B, Sahu R, Chilhate B, Mathur S, Krishna M, Sharma R, Sudhir P, King M, Cuijpers P, Chorpita B, Fairburn CG, Patel V. Effectiveness of a brief lay counsellor-delivered, problem-solving intervention for adolescent mental health problems in urban, low-income schools in India: a randomised controlled trial. Lancet Child Adolesc Health. 2020 Aug;4(8):571-582. doi: 10.1016/S2352-4642(20)30173-5. Epub 2020 Jun 23. Erratum in: Lancet Child Adolesc Health. 2020 Jul 23;: PMID: 32585185; PMCID: PMC7386943. |
| ^*+^ | O'Dea B, Subotic-Kerry M, King C, Mackinnon AJ, Achilles MR, Anderson M, Parker B, Werner-Seidler A, Torok M, Cockayne N, Baker STE, Christensen H. A cluster randomised controlled trial of a web-based youth mental health service in Australian schools. Lancet Reg Health West Pac. 2021 Jun 12;12:100178. |
| ^+^ | Olive, L. S., Byrne, D., Cunningham, R. B., Telford, R. M., & Telford, R. D. (2019). Can physical education improve the mental health of children? The LOOK study cluster-randomized controlled trial. Journal of Educational Psychology, 111(7), 1331–1340. |
| ^+^ | Osborn TL, Rodriguez M, Wasil AR, Venturo-Conerly KE, Gan J, Alemu RG, Roe E, Arango G S, Otieno BH, Wasanga CM, Shingleton R, Weisz JR. Single-session digital intervention for adolescent depression, anxiety, and well-being: Outcomes of a randomized controlled trial with Kenyan adolescents. J Consult Clin Psychol. 2020 Jul;88(7):657-668. (Osborn 2020a) |
| ^+^ | Osborn TL, Venturo-Conerly KE, Arango G S, Roe E, Rodriguez M, Alemu RG, Gan J, Wasil AR, Otieno BH, Rusch T, Ndetei DM, Wasanga C, Schleider JL, Weisz JR. Effect of Shamiri Layperson-Provided Intervention vs Study Skills Control Intervention for Depression and Anxiety Symptoms in Adolescents in Kenya: A Randomized Clinical Trial. JAMA Psychiatry. 2021 Aug 1;78(8):829-837. |
| ^+^ | Osborn TL, Wasil AR, Venturo-Conerly KE, Schleider JL, Weisz JR. Group Intervention for Adolescent Anxiety and Depression: Outcomes of a Randomized Trial with Adolescents in Kenya. Behav Ther. 2020 Jul;51(4):601-615. (Osborn 2020b) |
| ^*+^ | Pile V, Smith P, Leamy M, Oliver A, Bennett E, Blackwell SE, Meiser-Stedman R, Stringer D, Dunn BD, Holmes EA, Lau JYF. A feasibility randomised controlled trial of a brief early intervention for adolescent depression that targets emotional mental images and memory specificity (IMAGINE). Behav Res Ther. 2021 Aug;143:103876. |
| ^+^ | Poli A, Maremmani AGI, Gemignani A, Miccoli M. Randomized Trial on the Effects of a Mindfulness Intervention on Temperament, Anxiety, and Depression: A Multi-Arm Psychometric Study. Behav Sci (Basel). 2022 Mar 10;12(3):74. |
|  | Rivero LMHN, Andrade ALM, Figueredo LZP, Pinheiro BO, Micheli D. Evaluation of FunFRIENDS program in prevention of anxiety in Brazilian children: a randomized controlled pilot trial. Cien Saude Colet. 2020 Nov;25(11):4497-4508. |
| ^*+^ | Rodrigues JM, Matos LC, Francisco N, Dias A, Azevedo J, Machado J. Assessment of Qigong Effects on Anxiety of High-school Students: A Randomized Controlled Trial. Adv Mind Body Med. 2021 Summer;35(3):10-19. PMID: 34237025. |
| ^*+^ | Sælid GA, Czajkowski NO, Aarø LE, Andersen JR, Idsøe T, Helleseter MD, Holte A. Effects of a school-based intervention on levels of anxiety and depression: a cluster-randomized controlled trial of the MindPower program in ten high schools in Norway. BMC Psychol. 2022 Jan 24;10(1):14. |
| ^*+^ | Teesson M, Newton NC, Slade T, Chapman C, Birrell L, Mewton L, Mather M, Hides L, McBride N, Allsop S, Andrews G. Combined prevention for substance use, depression, and anxiety in adolescence: a cluster-randomised controlled trial of a digital online intervention. Lancet Digit Health. 2020 Feb;2(2):e74-e84. |
| ^+^ | Venturo-Conerly KE, Osborn TL, Alemu R, Roe E, Rodriguez M, Gan J, Arango S, Wasil A, Wasanga C, Weisz JR. Single-session interventions for adolescent anxiety and depression symptoms in Kenya: A cluster-randomized controlled trial. Behav Res Ther. 2022 Apr;151:104040. |
| ^+^ | Volanen SM, Lassander M, Hankonen N, Santalahti P, Hintsanen M, Simonsen N, Raevuori A, Mullola S, Vahlberg T, But A, Suominen S. Healthy learning mind - Effectiveness of a mindfulness program on mental health compared to a relaxation program and teaching as usual in schools: A cluster-randomised controlled trial. J Affect Disord. 2020 Jan 1;260:660-669. |
| ^*+^ | Waters AM, Candy SG, Zimmer-Gembeck MJ, Groth TA, Craske MG, Bradley BP, Mogg K. A School-Based Comparison of Positive Search Training to Enhance Adaptive Attention Regulation with a Cognitive-Behavioural Intervention for Reducing Anxiety Symptoms in Children. J Abnorm Child Psychol. 2019 Nov;47(11):1821-1840. |
|  | Zhao Y, Yu F, Wu Y, Zeng G, Peng K. Positive Education Interventions Prevent Depression in Chinese Adolescents. Front Psychol. 2019 Jun 12;10:1344. |
|  |  |

### Characteristics of included studies and risk of bias 1.0 assessments: Appendix Table 2

| **Review** | **Study** | **Design** | **Focus** | **Popul- ation** | **Setting** | **Age** | **Country** | **Ctrl** | **Int 1** | **Int2** | **Int3** | **RoB1** | **RoB2** | **Facilitated by** | **Mode** | **Format** |
| --- | --- | --- | --- | --- | --- | --- | --- | --- | --- | --- | --- | --- | --- | --- | --- | --- |
| 2019 | Ahlen 2018 | C | A+D | U | Primary | 8-11 | HIC | UC | CB |  |  | Low | Low | Teacher | F2F | Group |
| 2019 | Anticich 2013 | C | A | U | Primary | 4-7 | HIC | WL | PS | CB |  | Unclear | Unclear | Teacher | F2F | Group |
| 2019 | Araya 2013 | C | D | U | Secondary | 14.5 | MIC | UC | CB |  |  | Low | Unclear | Psychologist | F2F | Group |
| 2019 | Arnarson 2009 | I | D | T | Secondary | 14-15 | HIC | NI | CB+IP |  |  | Unclear | Unclear | Psychologist | F2F | Group |
| 2019 | Attwood 2012 | I | A | U | Primary | 10-12 | HIC | AC | CB |  |  | Unclear | Unclear | Researcher | MM | Group/  Individual |
| 2019 | Aune 2009 | C | A | U | Secondary | 10-15 | HIC | NI | CB |  |  | Unclear | Unclear | Psychologist | F2F | Group |
| 2019 | Baker 1984 | C | A | U | Secondary | 16-18 | HIC | CBSH | CB |  |  | Unclear | Unclear | Teacher | F2F | Group |
| 2019 | Balle 2010 | I | A | T | Secondary | 11-17 | HIC | WL | CB |  |  | Unclear | Unclear | Psychologist | F2F | Group |
| 2019 | Barrett 2001 | C | A | U | Primary | 10-12 | HIC | UC | CB | CB |  | Unclear | Unclear | Teachers or Psychologist | F2F | Group |
| 2019 | Barrett 2005 | C | A | U | Secondary | 9-16 | HIC | UC | CB |  |  | Unclear | Unclear | Psychologist | F2F | Group |
| 2019 | Barry 2017 | I | D | U | Secondary | 15-16 | HIC | UC | CB |  |  | Unclear | Unclear | "Coach" | F2F | Group |
| 2019 | Berry 2009 | C | A | T | Secondary | 12-15 | HIC | WL | CB |  |  | Low | Unclear | Psychologist | F2F | Group |
| 2019 | Bonhauser 2005 | C | A+D | U | Secondary | 15.3 | MIC | UC | EX |  |  | Unclear | Unclear | Teacher | F2F | Group |
| 2019 | Bouchard 2013 | I | A | U | Primary | 9-12 | HIC | WL | CB |  |  | Unclear | Unclear | Psychologist | F2F | Group |
| 2019 | Britton 2014 | I | A | U | Secondary | 11.79 | HIC | AC | MR |  |  | Low | Unclear | Teacher | F2F | Group |
| 2019 | Burckhardt 2015 | C | A+D | U | Secondary | 14-16 | HIC | AC | MR |  |  | Low | Unclear | NA | MM | Group |
| 2019 | Burckhardt 2016 | C | A+D | U | Secondary | 15-18 | HIC | UC | TW |  |  | Unclear | High | Psychologist | F2F | Group |
| 2019 | Calear 2009 | C | A+D | U | Secondary | 12-17 | HIC | WL | CB |  |  | Low | Low | Teacher | MM | Group |
| 2019 | Calear 2016 | C | A | U | Secondary | 12-18 | HIC | WL | CB | CB |  | Low | Low | Teacher or MHP | MM | Group |
| 2019 | Calear 2016b | C | A | U | Secondary | 13-17 | HIC | WL | CB |  |  | Unclear | Unclear | Teacher | MM | Group |
| 2019 | Cardemil 2002 | I | D | U | Primary | 10-12 | HIC | UC | CB |  |  | Unclear | Unclear | Psychologist | F2F | Group |
| 2019 | Chaplin 2006 | I | D | U | Secondary | 11-14 | HIC | NI | CB |  |  | Low | Unclear | Teacher & Researchers | F2F | Group |
| 2019 | Clarke 1993a | C | D | U | Secondary | 14-16 | HIC | UC | PE |  |  | Unclear | Unclear | Teacher | F2F | NA |
| 2019 | Clarke 1993b | C | D | U | Secondary | 14-16 | HIC | UC | BT |  |  | Unclear | Unclear | Teacher | F2F | NA |
| 2019 | Clarke 1995 | I | D | T | Secondary | 14-16 | HIC | NI | CB |  |  | Unclear | Unclear | School psychologist | F2F | Group |
| 2019 | Collins 2014 | C | A | U | Primary | 9-10 | HIC | UC | CB | CB |  | Unclear | Unclear | Teacher or school counsellor | F2F | Group |
| 2019 | Congelton 1995 | I | D | T | Secondary | 12-14 | HIC | WL | CB |  |  | Unclear | High | Psychologist | F2F | Group |
| 2019 | Cooley-Strickland 2011 | I | A | T | Primary | 9-10 | HIC | WL | CB |  |  | Unclear | Unclear | Psychologist | F2F | Group |
| 2019 | Cova 2011 | I | D | T | Secondary | 14-15 | MIC* | NI | CB |  |  | Unclear | Unclear | Psychologist | F2F | Group |
| 2019 | Cowell 2009 | C | D | T | Primary | 10.4 | HIC | NI | PS |  |  | Unclear | Unclear | Nurse | F2F | Group |
| 2019 | Dobson 2010 | I | A+D | T | Secondary | 13-18 | HIC | AC | CB |  |  | Low | Unclear | Psychologist | F2F | Group |
| 2019 | Eather 2016 | C | A+D | U | Secondary | 15-16 | HIC | WL | EX |  |  | Low | Low | Fitness instructor | F2F | Group |
| 2019 | Essau 2012 | C | A | U | Primary | 9-12 | HIC | WL | CB |  |  | Unclear | Unclear | Psychologist | F2F | Group |
| 2019 | Fitzgerald 2016 | I | A | T | Secondary | 15-18 | HIC | AC | BM |  |  | Unclear | Unclear | Researcher | MM | Group |
| 2019 | Fung 2016 | I | A+D | T | Secondary | 12-14 | HIC | WL | MR |  |  | Unclear | Unclear | Psychologist | F2F | Group |
| 2019 | Gaete 2016 | I | D | T | Secondary | 13-18 | MIC* | UC | CB |  |  | Low | Low | Psychologist | F2F | Group |
| 2019 | Gallegos 2008 | C | A+D | U | Primary | 9-11 | MIC | UC | CB |  |  | Unclear | Unclear | Teacher | F2F | Group |
| 2019 | Gillham 1994 | I | D | U | Primary | 10-12 | HIC | NI | CB | CB |  | Unclear | Unclear | Psychologist | F2F | Group |
| 2019 | Gillham 2006 | I | A+D | U | Secondary | 11-13 | HIC | NI | CB |  |  | Unclear | Unclear | Researchers & Psychologist | F2F | Group |
| 2019 | Gillham 2007 | I | D | U | Secondary | 11-14 | HIC | NI | PS | CB |  | Low | Unclear | Teachers/school counsellors/psychologists | F2F | Group |
| 2019 | Gillham 2012 | I | D | T | Secondary | 10-15 | HIC | NI | CB | CB |  | Low | Unclear | Teacher & school counsellor | F2F | Group |
| 2019 | Guhct 2017 | C | A+D | U | Secondary | 14-21 | HIC | UC | TW |  |  | Low | Unclear | Teacher | F2F | Group |
| 2019 | Haden 2014 | I | A+D | U | Primary | 10-11 | HIC | UC | MR |  |  | Unclear | High | Teacher | F2F | Group |
| 2019 | Hiebert 1989a | I | A | T | Secondary | 15-17 | HIC | WL | MR | BIO |  | Unclear | Unclear | Psychologist | F2F | Individual |
| 2019 | Hiebert 1989b | I | A | U | Secondary | 13-14 | HIC | AC | MR |  |  | Unclear | Unclear | Teacher & school counsellor | F2F | Group |
| 2019 | Hodas 2015 | I | A+D | U | Secondary | 12-14 | HIC | WL | CB |  |  | Unclear | Unclear | Psychologist | F2F | Group |
| 2019 | Horowitz 2007 | I | D | U | Secondary | 14-15 | HIC | UC | CB | IP |  | Low | High | Psychologist | F2F | Group |
| 2019 | Hunt 2009 | C | A | T | Secondary | 11-13 | HIC | NI | CB |  |  | Unclear | Unclear | Teacher & school counsellor | F2F | Group |
| 2019 | Jaycox 1994 | C | D | T | Primary | 10-13 | HIC | WL | CB |  |  | Unclear | Unclear | Psychologist | F2F | Group |
| 2019 | Johnson 2016 | C | A+D | U | Secondary | 13.63 | HIC | UC | TW |  |  | Low | Unclear | Psychologist | F2F | Group |
| 2019 | Johnson 2017 | C | A+D | U | Secondary | 13.44 | HIC | UC | TW | TW |  | Low | Unclear | Psychologist | F2F | Group |
| 2019 | Johnstone 2014 | C | A+D | U | Primary | 9-10 | HIC | UC | CB |  |  | Unclear | Unclear | Teacher | F2F | Group |
| 2019 | Jordans 2010 | C | A+D | T | Secondary | 11-14 | LIC | WL | Mixed (CB) |  |  | Low | Low | Researcher | F2F | Group |
| 2019 | Khalsa 2012 | C | A+D | U | Secondary | 15-19 | HIC | UC | MR |  |  | Unclear | Unclear | Yoga trainer | F2F | Group |
| 2019 | Kindt 2014 | C | D | U | Secondary | 11-16 | HIC | UC | CB |  |  | Low | Low | Teacher | F2F | Group |
| 2019 | Kiselica 1994 | I | A | T | Secondary | 14-15 | HIC | PS | CB |  |  | Unclear | Unclear | Counsellors | F2F | Group |
| 2019 | Liddle 2010 | I | A | T | Mixed | 8-14 | HIC | WL | CB |  |  | Unclear | Unclear | Psychologist | F2F | Group |
| 2019 | Livheim 2014 | I | D | T | Secondary | 12-17 | HIC | UC | TW |  |  | Low | Unclear | Psychologist | F2F | Group |
| 2019 | Lock 2003 | C | A | U | Secondary | NR | HIC | UC | CB |  |  | Unclear | Unclear | Teacher | F2F | Group |
| 2019 | Lowry-Webster 2001 | C | A+D | U | Secondary | 10-13 | HIC | WL | CB |  |  | Unclear | Unclear | Teacher | F2F | Group |
| 2019 | Manassis 2010 | I | A+D | T | Primary | 8-11 | HIC | AC | CB |  |  | Low | Unclear | Psychologist | F2F | Group |
| 2019 | McCarty 2011 | I | D | T | Secondary | 13 | HIC | UC | CB |  |  | Unclear | Unclear | Not clear | F2F | Group |
| 2019 | McCarty 2013 | I | D | T | Secondary | 11-15 | HIC | PS | CB |  |  | Low | Low | Therapists | F2F | Group |
| 2019 | McLaughlin 2011 | I | D | T | Mixed | 10-15 | HIC | PS | CB |  |  | Low | High | Psychologist | F2F | Group |
| 2019 | McLoone 2012 | I | A | T | Primary | 7-10 | HIC | WL | CB | CB |  | Low | Unclear | School counsellors | F2F | Group |
| 2019 | Mendelson 2010 | C | D | U | Primary | 9-11 | HIC | WL | MR |  |  | Unclear | Unclear | Yoga trainer | F2F | Group |
| 2019 | Merry 2004 | I | D | U | Secondary | 13-15 | HIC | AC | CB+IP |  |  | Low | Low | Teacher | F2F | Group |
| 2019 | Mifsud 2005 | C | A | T | Primary | 8-11 | HIC | WL | CB |  |  | Unclear | Unclear | School counsellors | F2F | Group |
| 2019 | Miller 2010 | C | A | U | Primary | 7-12 | HIC | WL | CB |  |  | Unclear | Unclear | Teacher | F2F | Group |
| 2019 | Miller 2011a | C | A | U | Primary | 7-13 | HIC | WL | CB |  |  | Unclear | Unclear | Teacher & school counsellor | F2F | Group |
| 2019 | Miller 2011b | C | A | T | Primary | 7-12 | HIC | AC | CB |  |  | Unclear | Unclear | Teacher & school counsellor | F2F | Group |
| 2019 | Miller 2011c | C | A | U | Primary | 7-13 | HIC | AC | CB |  |  | Unclear | Unclear | Teacher & school counsellor | F2F | Group |
| 2019 | Noël 2013 | I | D | T | Secondary | 13-15 | HIC | WL | CB |  |  | Low | Unclear | Students | F2F | Group |
| 2019 | Owen 1982 | I | A | T | Secondary | 15-16 | HIC | WL | MR | CB | CB | Unclear | Unclear | Counsellors | F2F | Group |
| 2019 | Pahl 2010 | C | A | U | Primary | 4-6 | HIC | WL | CB |  |  | Unclear | Unclear | Psychologist | F2F | Group |
| 2019 | Pattison 2001 | I | D | U | Primary | 9-12 | HIC | NI | AC | CB | CB | Unclear | Unclear | Child mental health professionals | F2F | Group |
| 2019 | Perry 2017 | C | D | U | Secondary | 16-17 | HIC | AC | CB |  |  | Unclear | Low | Self | MM | Group |
| 2019 | Pophillat 2016 | C | A+D | U | Primary | 6-8 | HIC | UC | CB |  |  | Unclear | Unclear | Teacher | F2F | Group |
| 2019 | Poppelaars 2016 | C | D | T | Secondary | 11-16 | HIC | WL | CB | CB | CB | Unclear | Low | Psychologist | F2F | Individual |
| 2019 | Possel 2004 | C | D | U | Secondary | 13-14 | HIC | UC | CB |  |  | Unclear | Unclear | Psychologist or graduate students | F2F | Group |
| 2019 | Possel 2008 | C | D | U | Secondary | 12-13 | HIC | UC | CB |  |  | Unclear | Unclear | Psychologist or graduate students | F2F | Group |
| 2019 | Possel 2013 | C | D | U | Secondary | 14-16 | HIC | UC | CB | PS |  | Unclear | Unclear | Psychologist or graduate students | F2F | Group |
| 2019 | Potek 2012 | I | A | U | Secondary | 14-17 | HIC | WL | MR |  |  | Unclear | Unclear | Psychologist | F2F | Group |
| 2019 | Puskar 2003 | I | D | T | Secondary | 14-18 | HIC | NI | CB |  |  | Low | Unclear | Nurse | F2F | Group |
| 2019 | Quayle 2001 | I | D | U | Primary | 11-12 | HIC | WL | CB |  |  | Unclear | Unclear | Psychologist | F2F | Group |
| 2019 | Raes 2014 | C | D | U | Secondary | 13-20 | HIC | UC | TW |  |  | Low | Low | Psychologist | F2F | Group |
| 2019 | Rice 2008 | I | A | T | Secondary | 10-18 | HIC | AC | MR | CB |  | Unclear | Unclear | Psychologist | F2F | Group |
| 2019 | Rivet-Duval 2011 | I | D | U | Secondary | 12-16 | MIC | WL | CB+IP |  |  | Unclear | High | Teacher | F2F | Group |
| 2019 | Roberts 2003 | C | D | U | Secondary | 11-13 | HIC | UC | CB |  |  | Unclear | Unclear | Psychologist | F2F | Group |
| 2019 | Roberts 2010 | C | A+D | U | Secondary | 11-13 | HIC | UC | CB |  |  | Unclear | Unclear | Teacher | F2F | Group |
| 2019 | Roberts 2018 | C | A+D | U | Primary | 9-12 | HIC | UC | CB | CB |  | Unclear | Unclear | Teacher | F2F | Group |
| 2019 | Rodgers 2015 | I | A | U | Secondary | 12-13 | HIC | WL | CB |  |  | Unclear | Unclear | Psychologist | F2F | Group |
| 2019 | Rohde 2014 | I | D | T | Secondary | 13-19 | HIC | PE | CBSH | CB |  | Low | Unclear | Psychologist or self-help | F2F | Group |
| 2019 | Rooney 2006 | C | D | U | Primary | 8-9 | HIC | UC | CB |  |  | Unclear | Unclear | Psychologist | F2F | Group |
| 2019 | Rose 2014 | C | D | U | Secondary | 9-14 | HIC | WL | CB+IP | CB+IP |  | Unclear | Unclear | Psychologist | F2F | Group |
| 2019 | Ruttledge 2016 | C | A | U | Primary | 9-13 | HIC | WL | CB |  |  | Low | Unclear | Teacher | F2F | Group |
| 2019 | Sawyer 2010 | C | D | U | Secondary | 13.1 | HIC | UC | CB |  |  | Unclear | Low | Teacher | F2F | Group |
| 2019 | Scholten 2016 | I | A | T | Secondary | 11-15 | HIC | AC | Bio |  |  | Low | Unclear | Researcher | MM | Individual |
| 2019 | Schoneveld 2016 | I | A | T | Primary | 8-13 | HIC | AC | Bio |  |  | Low | Low | Researcher | MM | Group |
| 2019 | Schoneveld 2018 | I | A | T | Primary | 7-12 | HIC | CB | Bio |  |  | Low | Low | Masters students, psychologist | MM | Group |
| 2019 | Shatte 1997 | I | D | U | Secondary | 12-14 | HIC | NI | CB | PS |  | Unclear | Unclear | Teachers & Psychologist | F2F | Group |
| 2019 | Sheffield 2006a | C | D | U | Secondary | 13-15 | HIC | NI | CB |  |  | Low | Low | Teachers & Psychologist | F2F | Group |
| 2019 | Sheffield 2006b | C | D | T | Secondary | 13-15 | HIC | NI | CB | CB | CB | Low | Low | Teachers or school counsellor or both | F2F | Group |
| 2019 | Simpson 2008 | I | A+D | T | Primary | 7-11 | HIC | AC | CB |  |  | Unclear | Unclear | NR | F2F | Group |
| 2019 | Siu 2007 | I | A+D | T | Primary | 7-10 | HIC | WL | CB |  |  | Unclear | Unclear | Counsellors | F2F | Group |
| 2019 | Soffer 2003 | I | D | U | Primary | 10-11 | HIC | NI | AC | BT |  | Unclear | Unclear | Psychologist | F2F | Group |
| 2019 | Spence 2003 | C | D | U | Secondary | 12-14 | HIC | UC | CB |  |  | Unclear | Unclear | Teacher | F2F | Group |
| 2019 | Sportel 2013 | C | A | T | Secondary | 12-15 | HIC | NI | BM | CB |  | Low | Low | Self | MM | Individual |
| 2019 | Stallard 2012a | C | D | U | Secondary | 12-16 | HIC | UC | UC | CB+IP |  | Low | Low | Facilitator | F2F | Group |
| 2019 | Stallard 2012b | C | D | T | Secondary | 12-16 | HIC | UC | UC | CB+IP |  | Low | Low | ‘Facilitator’ | F2F | Group |
| 2019 | Stallard 2014 | C | A | U | Primary | 9-10 | HIC | UC | CB | CB |  | Low | Unclear | Teacher & Facilitator | F2F | Group |
| 2019 | Stice 2008 | I | D | T | Secondary | 14-19 | HIC | PE | CBSH | PS | CB | Low | Unclear | Self-help or Psychologist | F2F | Group |
| 2019 | Stoppelbein 2003 | C | D | T | Secondary | 15 | HIC | AC | CB |  |  | Unclear | Unclear | Psychologist | F2F | Group |
| 2019 | Tak 2016 | C | D | U | Secondary | 12-14 | HIC | UC | CB |  |  | Unclear | Low | Teacher & Psychologist | F2F | Group |
| 2019 | Tokolahi 2018 | C | A+D | T | Primary | 7-12 | HIC | WL | OT |  |  | Low | Low | Occupational Therapist | F2F | Group |
| 2019 | Tomba 2010 | C | A+D | U | Secondary | 11.41 | HIC | CB | CB |  |  | Unclear | Unclear | Psychologist | F2F | Group |
| 2019 | Topper 2017 | I | A+D | T | Secondary | 15-22 | HIC | WL | CB |  |  | Unclear | Low | Psychologist | F2F | Group |
| 2019 | van Starrenburg 2017 | I | A | T | Primary | 7-13 | HIC | WL | CB |  |  | Unclear | Unclear | Psychologist | F2F | Group |
| 2019 | Velásquez 2015 | I | A+D | U | Mixed | NR | MIC | WL | MR |  |  | Unclear | Unclear | Yoga trainer | F2F | Group |
| 2019 | Wijnhoven 2014 | I | D | T | Secondary | 11-15 | HIC | WL | CB |  |  | Low | Low | Therapist | F2F | Group |
| 2019 | Wong 2014 | C | A+D | U | Secondary | 14-16 | HIC | UC | CB | CB |  | Low | High | Teacher | MM | Group |
| 2019 | Woods 2011 | I | D | T | Secondary | 14 | HIC | UC | CB |  |  | Low | Unclear | School counsellors | F2F | Group |
| 2019 | Young 2006 | I | D | T | Secondary | 11-16 | HIC | PS | IP |  |  | Low | Unclear | Psychologist/ Social worker | F2F | Group/individual |
| 2019 | Young 2010 | I | D | T | Secondary | 13-17 | HIC | PS | IP |  |  | Low | Unclear | Psychologist | F2F | Group/individual |
| 2019 | Young 2016 | I | D | T | Secondary | 13.42 | HIC | PS | IP |  |  | Low | Unclear | Psychologist | F2F | Group/individual |
| 2019 | Yu 2002 | I | D | T | Mixed | 8-15 | MIC | NI | CB |  |  | Unclear | Unclear | Teacher | F2F | Group |
| 2023 | Ab Ghaffar 2019 | C | A | U | Primary | 10-11 | MIC | UC | PE |  |  | Low | Unclear | Research assistants | F2F | Group |
| 2023 | Bazzano 2022 | C | A+D | U | Secondary | 11-14 | HIC | WL | MR |  |  | Low | Unclear | Yoga teachers | F2F | Group |
| 2023 | Briere 2019 | I | D | T | Secondary | 14-18 | HIC | PE | CB |  |  | Unclear | Unclear | Psycho-educator or psychologist | F2F | Group |
| 2023 | Calvete 2019 | I | D | U | Secondary | 12-17 | HIC | AC | PP |  |  | Unclear | Low | Psychologist | F2F | Group |
| 2023 | Chen 2022 | C | A+D | U | Primary | 8-13 | HIC | UC | BT | CB |  | Low | Unclear | A provisional psychologist and research assistant | F2F | Group |
| 2023 | David 2019 | I | A+D | U | Secondary | 10-16 | HIC | WL | CB | CB |  | Low | Unclear | Self | F2F & MM | Group |
| 2023 | de Jonge-Heesen 2021 | I | D | T | Secondary | 12-16 | HIC | PE | CB |  |  | Low | Unclear | School psychologists | F2F | Group |
| 2023 | Fernandez-Martinez 2020 | C | A+D | T | Primary | 6-8 | HIC | WL | CB |  |  | Unclear | Unclear | Psychologists | F2F | Group |
| 2023 | Garaigordobil 2019 | C | D | U | Primary | 7-10 | HIC | PP | CB |  |  | Unclear | Unclear | Teachers | F2F | Group |
| 2023 | Garcia-Escalera 2020 | C | A+D | U | Secondary | 15.05 | HIC | WL | TW |  |  | Low | High | Researcher & psychology graduate student | F2F | Group |
| 2023 | Hamdani 2022 | C | A+D | T | Secondary | 13-15 | MIC | NI | CB |  |  | Low | Low | Non-specialist facilitators | F2F | Group |
| 2023 | Harris 2022 | C | A+D | U | Secondary | 11-13 | HIC | UC | EX |  |  | Unclear | Low | Teachers | F2F | Group |
| 2023 | Haugland 2020* | I | A | T | Secondary | 12-16 | HIC | WL | CB |  |  | Low | Unclear | School nurses & mental health professionals | F2F | Group |
| 2023 | Humphrey 2022 | C | A+D | T | Primary | 9-11 | HIC | WL | BT |  |  | Low | Unclear | Youth practitioner | F2F | Group |
| 2023 | Johnstone 2020 | C | A+D | U | Primary | 8-13 | HIC | UC | BT | CB |  | Low | High | A provisional psychologist and research assistant | F2F | Group |
| 2023 | Khanna 2019 | C | D | U | Secondary | 11-13 | MIC | AC | PP |  |  | Unclear | Unclear | Researcher | F2F | Group |
| 2023 | Klim-Conforti 2021 | C | A+D | U | Secondary | 11-14 | HIC | WL | CB |  |  | High | High | Teachers | F2F | Group |
| 2023 | Kuyken 2022 | C | A+D | U | Secondary | 11-16 | HIC | UC | MR |  |  | Low | Low | Teachers | F2F | Group |
| 2023 | Lima 2022 | C | D | U | Secondary | 13-18 | HIC | UC | EX | EX |  | Unclear | Unclear | PE teachers | F2F | Group |
| 2023 | Maalouf 2020 | C | A+D | U | Secondary | 12 | MIC | WL | CB |  |  | Unclear | Unclear | Mental health professionals or trainees | F2F | Group |
| 2023 | Makover 2019 | I | A+D | T | Secondary | 13-14 | HIC | PS | BT |  |  | Low | Unclear | Mental health counsellors | F2F | Group |
| 2023 | Martinsen 2019 | C | A+D | T | Primary | 8-12 | HIC | PS | CB |  |  | Unclear | Unclear | Psychologists & school counsellors | F2F | Group |
| 2023 | Michelson 2020 | I | A+D | T | Secondary | 12-20 | MIC | PE | BT |  |  | Low | Low | Lay counsellors | F2F | Individual |
| 2023 | O'Dea 2021 | C | A+D | U | Secondary | 14.3 | HIC | UC | CB |  |  | Low | Unclear | Self/school staff | MM | Individual |
| 2023 | Olive 2019 | C | D | U | Primary | 8.1 | HIC | UC | EX |  |  | Low | Unclear | PE teachers | F2F | Group |
| 2023 | Osborn 2020a | I | A+D | U | Secondary | 13-18 | MIC | AC | PP |  |  | Low | Unclear | Lay group leaders | F2F | Group |
| 2023 | Osborn 2020b | I | A+D | T | Secondary | 14-17 | MIC | AC | PP |  |  | Low | Low | Self | MM | Group |
| 2023 | Osborn 2021 | I | A+D | T | Secondary | 13-18 | MIC | AC | PP |  |  | Low | Unclear | Lay group leaders | F2F | Group |
| 2023 | Pile 2021 | I | D | T | Secondary | 16-18 | HIC | PS | CB |  |  | Low | Low | Clinical Psychologist | F2F | Individual |
| 2023 | Poli 2022 | I | A+D | U | Primary | 9-11 | HIC | UC | MR |  |  | Low | Unclear | Psychotherapist | F2F | Group |
| 2023 | Rivero 2020 | I | A+D | U | Primary | 4-6 | MIC | WL | CB |  |  | Unclear | Unclear | Psychologists | F2F | Group |
| 2023 | Rodrigues 2021 | I | A | U | Secondary | 13-18 | HIC | UC | AC | MR |  | Low | Unclear | Qigong therapist | F2F | Group |
| 2023 | Saelid 2022 | C | A+D | U | Secondary | 15-16 | HIC | WL | CB |  |  | Low | High | Teachers | F2F | Group |
| 2023 | Teesson 2020 | C | A+D | U | Secondary | 13-14 | HIC | UC | CB | CB |  | Low | High | Self and teacher | Mixed | Group |
| 2023 | Venturo-Conerly 2022a | C | A+D | U | Secondary | 14-18 | MIC | AC | PP | PP | PP | Low | Unclear | Lay providers | F2F | Group |
| 2023 | Volanen 2020 | C | D | U | Secondary | 12-15 | HIC | UC | MR | MR |  | Low | Unclear | Mindfulness facilitators | F2F | Group |
| 2023 | Waters 2019 | C | A+D | U | Primary | 7-11 | HIC | UC | CB | BM |  | Low | Unclear | BM: Self + Researcher, CB: clinical psychologist | MM F2F | Group |
| 2023 | Zhao 2019 | C | D | U | Secondary | 13.54 | MIC | AC | PP |  |  | Unclear | Unclear | Teacher | F2F | Group |

**Key**: Review: study identified by original (2019) or update (2023) review. C: cluster randomised trial, I: individual randomised trial, D: depression, A: anxiety, A+D: anxiety and depression, U: universal population, T: targeted population. HIC: high income country, MIC: middle-income country, LIC: low-income country. RoB1: random sequence generation. RoB2: adequacy of allocation concealment. *Haughland2020 is a 3-arm trial but data extracted for this review were reported as a combined CB arm.

AC: attention control, NI: no intervention, UC:  usual curriculum, WL: wait list. BIO: biofeedback, BM: bias modification, BT: behavioural, CB: cognitive behavioural, EX: exercise, IP: interpersonal therapy, MR: mindfulness/relaxation interventions, OT: occupational therapy, PE: education, PP: positive psychology, PS: psupportive, SH: self-help, TW: third wave. F2F: face to face, MM: multi-media/digital/computer-based

### Percentage of studies reporting each control group type, by 5-year periods: Appendix Table 3

| Year | UC | WL | NI | AC | PS | PE | Other |
| --- | --- | --- | --- | --- | --- | --- | --- |
| <=2000 (na=14; N=12) | 14% | 29% | 21% | 7% | 14% | 7% | 7% |
| 2001-2005 (na=19, N=17) | 42% | 16% | 21% | 21% | 0% | 0% | 0% |
| 2006-2010 (na=35, N=32) | 17% | 26% | 26% | 11% | 14% | 3% | 3% |
| 2011-2015 (na=44, N=40) | 39% | 32% | 7% | 11% | 9% | 2% | 0% |
| 2016-2020 (na=48, N=47) | 33% | 31% | 0% | 19% | 6% | 6% | 4% |
| 2021- 06/2023 (na=16, N=15) | 44% | 25% | 6% | 13% | 6% | 6% | 0% |

Cell data are the percentage of arms in each 5-year period, including each control group type

UC: usual curriculum, WL: waiting list, NI: no intervention, AC: attention control, PS; supportive, PE:education. ‘Other’ includes active interventions such as cognitive behavioural and behavioural comparator arms.

N=number of studies, na= number of study arms. Number of study arms includes multi-arm studies where more than one control group was reported (164 studies and 176 control/ comparator arms).

### Full network diagrams: combined population and educational setting

#### Depression: universal and targeted, primary and secondary educational settings

#### Anxiety: universal and targeted, primary and secondary educational settings

Combined network shown for studies in primary and secondary educational settings and for universal and targeted populations. The solid black lines denote direct evidence comparing each pair of interventions, with the width proportional to the number of studies. The size of each node (circle) is proportional to the number of participants receiving that intervention across all studies.

AC: attention control, BM: bias modification, BIO: biofeedback, CBI: cognitive behavioural, MR: mindfulness/ relaxation, NI: no intervention, OT: occupational therapy, PP: positive psychology, PE: education, PS: supportive, TW: third wave, UC: usual curriculum, WL: waiting list.

### Separate educational setting and population specific networks

**Universal depression**: (a) Secondary educational setting, (b) Primary educational setting

**Universal anxiety**: (a) Secondary educational setting, (b) Primary educational setting

**Targeted depression:** (a) Secondary educational setting, (b) Primary educational setting

**Targeted anxiety:** (a) Secondary educational setting, (b) Primary educational setting

### Baseline and study level characteristics by comparison and analysis for assessment of transitivity: Appendix Table 4

| **Study** | **Population** | **Setting** | **Comparison** | **Gender** | **Age range** | **Mean age** | **Sessions** | **Intensity (mins)** | **Anxiety scale** | **Baseline Score** | **Depression scale** | **Baseline score** |
| --- | --- | --- | --- | --- | --- | --- | --- | --- | --- | --- | --- | --- |
| Attwood 2012 | Universal | Primary | AC_CB | Male | 10-12 | 10.6 | 6 | 270 | SCAS | 23.3 | NR | NR |
| Miller 2011c | Universal | Primary | AC_CB | Mixed | 7-13 | 9.8 | 9 | 540 | MASC | 47.3 | NR | NR |
| Soffer 2003 | Universal | Primary | NI_AC_BT | Mixed | 10-11 | 10.5 | 8 | 320 | NR | NR | RCDS | 50.9 |
| Pattison 2001 | Universal | Primary | NI_AC_CB_CB | Mixed | 9-12 | 10.4 | 10 | 1200 | STAI-C | 33.0 | CDI | 7.9 |
| Gillham 1994 | Universal | Primary | NI_CB | Mixed | 10-12 | . | 12 | 1440 | NR | NR | CDI | 7.5 |
| Garaigordobil 2019 | Universal | Primary | PP_CB | Mixed | 7-10 | . | 18 | . | NR | NR | CDS | 138.3 |
| Ahlen 2018 | Universal | Primary | UC_CB | Mixed | 8-11 | 9.6 | 10 | 600 | SCAS | 26.9 | CDI | 1.8 |
| Cardemil 2002 | Universal | Primary | UC_CB | Mixed | 10-12 | 11.1 | 12 | 1080 | NR | NR | CDI | 11.2 |
| Gallegos 2008 | Universal | Primary | UC_CB | Mixed | 8-13 | 9.9 | 10 | 600 | SCAS | 30.2 | CDI | 9.4 |
| Johnstone 2014 | Universal | Primary | UC_CB | Mixed | 9-10 | 8.8 | 10 | 600 | SCAS | 31.6 | CDI | 12.1 |
| Pophillat 2016 | Universal | Primary | UC_CB | Mixed | 6-8 | . | 10 | . | SCAS | 33.4 | CDI | 11.7 |
| Rooney 2006 | Universal | Primary | UC_CB | Mixed | 8-9 | 9.1 | 8 | 480 | RCMAS | 13.8 | CDI | 14.0 |
| Waters 2019 | Universal | Primary | UC_CB_BM | Mixed | 7-11 | 9.1 | 8-9 | 240-270 | SCAS-C | 5.6 | SMFQ-C | 1.7 |
| Barrett 2001 | Universal | Primary | UC_CB_CB | Mixed | 10-12 | 10.8 | 10 | 750 | RCMAS | 10.7 | CDI | 9.2 |
| Collins 2014 | Universal | Primary | UC_CB_CB | Mixed | 9-10 | . | 10 | . | SCAS | 27.9 | NR | NR |
| Olive 2019 | Universal | Primary | UC_EX | Mixed | . | 8.1 | 288 | 14400 | NR | NR | CDI | 25.7 |
| Poli 2022 | Universal | Primary | UC_MR | Mixed | 9-11 | 10.8 | 8 | 480 | TAD | 107.5 | TAD | 106.2 |
| Ab Ghaffar 2019 | Universal | Primary | UC_PE | Mixed | 10-11 | 10.5 | 4 | 240 | RCADS25 | 14.7 | NR | NR |
| Bouchard 2013 | Universal | Primary | WL_CB | Mixed | 9-12 | 10.4 | 10 | 750 | MASC | 42.8 | NR | NR |
| Essau 2012 | Universal | Primary | WL_CB | Mixed | 9-12 | 10.9 | 10 | 600 | SCAS | 23.3 | RCADS | 6.4 |
| Miller 2010 | Universal | Primary | WL_CB | Mixed | 7-12 | 9.8 | . | . | MASC | 49.8 | NR | NR |
| Miller 2011a | Universal | Primary | WL_CB | Mixed | 7-13 | 9.8 | 9 | . | MASC | 46.2 | NR | NR |
| Quayle 2001 | Universal | Primary | WL_CB | Female | 11-12 | . | 8 | 640 | NR | NR | CDI | 7.4 |
| Ruttledge 2016 | Universal | Primary | WL_CB | Mixed | 9-13 | 10.8 | 10 | . | SCAS | 23.6 | NR | NR |
| Perry 2017 | Universal | Secondary | AC_CB | Mixed | 16-17 | 16.7 | 7 | 175 | SCAS-GA | 6.8 | Major Depression Inventory | 14.6 |
| Merry 2004 | Universal | Secondary | AC_CB+IP | Mixed | 13-15 | 14.2 | 11 | . | NR | NR | RADS | 54.7 |
| Hiebert 1989b | Universal | Secondary | AC_MR | Mixed | 13-14 | . | 11 | 660 | STAI | 39.3 | NR | NR |
| Calvete 2019 | Universal | Secondary | AC_PP | Mixed | 12-17 | 14.5 | 1 | 60 | NR | NR | CES-D | 17.3 |
| Khanna 2019 | Universal | Secondary | AC_PP | Mixed | 11-13 | 12.7 | 5 | . | NR | NR | CES-D | 17.7 |
| Osborn 2020a | Universal | Secondary | AC_PP | Mixed | 13-18 | 15.5 | 1 | . | GAD7 | 8.9 | PHQ8 | 10.1 |
| Venturo-Conerly 2022a | Universal | Secondary | AC_PP_PP_PP | Mixed | 14-18 | 16.0 | 1 | 60 | GAD7 | 8.1 | PHQ8 | 8.3 |
| Baker 1984 | Universal | Secondary | CB_CB | Mixed | 16-18 | . | 8 | 360 | STAI | 51.1 | NR | NR |
| Tomba 2010 | Universal | Secondary | CB_CB | Mixed | 11.41 | 11.4 | 6 | 720 | RCMAS | 9.3 | Kellner | 3.3 |
| Aune 2009 | Universal | Secondary | NI_CB | Mixed | 10-15 | 12.6 | 3 | 135 | SCARED | 10.9 | SMFQ | 4.0 |
| Chaplin 2006 | Universal | Secondary | NI_CB | Female | 11-14 | 12.2 | 12 | 1080 | NR | NR | CDI | 7.6 |
| Gillham 2006 | Universal | Secondary | NI_CB | Mixed | 11-13 | . | 8 | 720 | RCMAS | 11.1 | CDI | 10.8 |
| Sheffield 2006a | Universal | Secondary | NI_CB | Mixed | 13-15 | 14.3 | 8 | 380 | SCAS | 23.9 | CDI | 11.1 |
| Gillham 2007 | Universal | Secondary | NI_PS_CB | Mixed | 11-14 | 12.1 | 12 | 1080 | NR | NR | CDI | 8.5 |
| Shatte 1997 | Universal | Secondary | NI_PS_CB | Mixed | 12-14 | 12.7 | 12 | 1440 | NR | NR | CDI | 12.0 |
| Rodrigues 2021 | Universal | Secondary | UC_AC_MR | Mixed | 13-18 | . | 8 | 160 | STAI | 39.7 | NR | NR |
| Clarke 1993b | Universal | Secondary | UC_BT | Mixed | 14-16 | 15.1 | 5 | 250 | NR | NR | CES-D | 16.7 |
| Barry 2017 | Universal | Secondary | UC_CB | Male | 15-16 | 15.7 | 4 | . | NR | NR | CED-D-C | 11.0 |
| Kindt 2014 | Universal | Secondary | UC_CB | Mixed | 11-16 | 13.4 | 16 | . | NR | NR | CDI | 8.6 |
| Lock 2003 | Universal | Secondary | UC_CB | Mixed | . | . | 10 | 750 | RCMAS | 10.4 | CDI | 9.3 |
| O'Dea 2021 | Universal | Secondary | UC_CB | Mixed | 11-19 | 14.3 | . | . | GAD7 | 6.4 | CES-D | 17.5 |
| Possel 2004 | Universal | Secondary | UC_CB | Mixed | 13-14 | 14.0 | 10 | 900 | NR | NR | CES-D | 15.2 |
| Possel 2008 | Universal | Secondary | UC_CB | Mixed | 12-13 | 13.7 | 10 | 900 | NR | NR | Self-report questionnaire - Depression | 0.6 |
| Roberts 2003 | Universal | Secondary | UC_CB | Mixed | 11-13 | 11.9 | 12 | . | RCMAS | 10.3 | CDI | 10.0 |
| Roberts 2010 | Universal | Secondary | UC_CB | Mixed | 11-13 | 12.0 | 20 | 1200 | RCMAS | 8.0 | CDI | 7.9 |
| Spence 2003 | Universal | Secondary | UC_CB | Mixed | 12-14 | 12.9 | 8 | 380 | NR | NR | BDI | 44.4 |
| Tak 2016 | Universal | Secondary | UC_CB | Mixed | 12-14 | 13.91 | 16 | 800 | NR | NR | CDI | 7.6 |
| Teesson 2020 | Universal | Secondary | UC_CB_CB | Mixed | 13-14 | 13.5 | 6-12 | 240-480 | GAD7 | 3.6 | PHQ8 | 4.5 |
| Wong 2014 | Universal | Secondary | UC_CB_CB | Mixed | 14-16 | . | 6 | 240 | GAD7 | 4.6 | PHQ5 | 2.9 |
| Horowitz 2007 | Universal | Secondary | UC_CB_IP | Mixed | 14-15 | 14.4 | 8 | 720 | NR | NR | CDI | 9.6 |
| Lima 2022 | Universal | Secondary | UC_EX_EX | Mixed | 13-18 | . | . | . | NR | NR | CES-D | 17.0 |
| Khalsa 2012 | Universal | Secondary | UC_MR | Mixed | 15-19 | 16.8 | 27.5 | 825 | BASC-2 | NE | BASC | NE |
| Kuyken 2022 | Universal | Secondary | UC_MR | Mixed | 11-16 | 12.2 | 10 | 300 | RCADS | NE | CES-D | 13.5 |
| Volanen 2020 | Universal | Secondary | UC_MR_MR | Mixed | 12-15 | . | 9 | 405 | NR | NR | BDI | 2.7 |
| Clarke 1993a | Universal | Secondary | UC_PE | Mixed | 14-16 | 15.4 | 3 | 150 | NR | NR | CES-D | 17.1 |
| Possel 2013 | Universal | Secondary | UC_PS_CB | Mixed | 14-16 | 15.1 | 10 | 900 | NR | NR | CDI | 10.8 |
| Burckhardt 2016 | Universal | Secondary | UC_TW | Mixed | 15-18 | 16.4 | 16 | 480 | DASS | 9.5 | DASS | 11.3 |
| Guhct 2017 | Universal | Secondary | UC_TW | Mixed | 14-21 | 17.0 | 4 | 480 | YSR | 3.4 | YSR | 5.7 |
| Johnson 2016 | Universal | Secondary | UC_TW | Mixed | . | 13.6 | 9 | 495 | DASS | 0.5 | DASS | 0.6 |
| Raes 2014 | Universal | Secondary | UC_TW | Mixed | 14-17 | . | 8 | 800 | NR | NR | DASS | 20.3 |
| Johnson 2017 | Universal | Secondary | UC_TW_TW | Mixed | . | 13.4 | 9 | 450 | DASS | 0.9 | DASS | 0.8 |
| Calear 2009 | Universal | Secondary | WL_CB | Mixed | 12-17 | 14.3 | 5 | 150 | RCMAS | 8.9 | CES-D | 11.8 |
| Calear 2016 | Universal | Secondary | WL_CB | Mixed | 12-17 | 14.8 | 6 | 210 | GAD7 | 4.6 | CES-D | 17.6 |
| Calear 2016b | Universal | Secondary | WL_CB | Mixed | 13-17 | 15.0 | 6 | 210 | GAD7 | 4.9 | CES-D | 16.5 |
| Hodas 2015 | Universal | Secondary | WL_CB | Female | 12-14 | . | 7 | 455 | RCMAS | 11.0 | CDI | 9.0 |
| Lowry-Webster 2001 | Universal | Secondary | WL_CB | Mixed | 10-13 | . | 10 | 600 | RCMAS | 11.6 | CDI | 10.5 |
| Maalouf 2020 | Universal | Secondary | WL_CB | Mixed | . | 12.0 | 10 | . | SCARED | 28.9 | MFQ | 16.4 |
| Rodgers 2015 | Universal | Secondary | WL_CB | Mixed | 12-13 | . | 10 | 600 | SCAS | 22.8 | NR | NR |
| Saelid 2022 | Universal | Secondary | WL_CB | Mixed | 15-16 | . | 8 | 720 | NR | NR | RADS | 19.2 |
| Rivet-Duval 2011 | Universal | Secondary | WL_CB+IP | Mixed | 12-16 | 14.0 | 11 | 660 | NR | NR | RADS2 | 51.2 |
| Rose 2014 | Universal | Secondary | WL_CB+IP _CB+IP | Mixed | 9-14 | 12.2 | 11 | 495 | NR | NR | CDI | 7.8 |
| Bazzano 2022 | Universal | Secondary | WL_MR | Mixed | 11-14 | . | 8 | 360 | SCARED | 11.8 | PHQ | 5.0 |
| Potek 2012 | Universal | Secondary | WL_MR | Mixed | 14-17 | 15.0 | 6 | 270 | MASC | 62.2 | NR | NR |
| Schoneveld 2016 | Targeted | Primary | AC_Bio | Mixed | 8-13 | 10.0 | 5 | 300 | SCAS | 0.9 | NR | NR |
| Manassis 2010 | Targeted | Primary | AC_CB | Mixed | 8-11 | . | 12 | 720 | MASC | 61.2 | CDI | 57.3 |
| Miller 2011b | Targeted | Primary | AC_CB | Mixed | 7-12 | 10.1 | 9 | 540 | MASC | 57.2 | NR | NR |
| Simpson 2008 | Targeted | Primary | AC_CB | Mixed | 7-11 | 9.0 | 12 | 1080 | MASC | 58.2 | CDI | 52.7 |
| Schoneveld 2018 | Targeted | Primary | CB_Bio | Mixed | 7-12 | 10.0 | 6 | 360 | SCAS | 1.0 | NR | NR |
| Martinsen 2019* | Targeted | Primary | PS_CB | Mixed | 8-12 | 10.1 | 20 | . | MASC-C | 63.4 | SMFQ-C | 9.9 |
| Cooley-Strickland 2011 | Targeted | Primary | WL_CB | Mixed | 8-12 | 9.4 | 13 | 780 | RCMAS | 16.5 | NR | NR |
| Jaycox 1994 | Targeted | Primary | WL_CB | Mixed | 10-13 | 11.4 | 12 | 1080 | NR | NR | CDI | 9.5 |
| Mifsud 2005 | Targeted | Primary | WL_CB | Mixed | 8-11 | 9.5 | 8 | 480 | SCAS | 48.6 | NR | NR |
| Siu 2007 | Targeted | Primary | WL_CB | Mixed | 7-10 | 8.4 | 8 | . | SCARED | 31.3 | RCDS | 55.2 |
| van Starrenburg 2017 | Targeted | Primary | WL_CB | Mixed | 7-13 | 9.5 | 12 | 720 | SCAS | 0.8 | NR | NR |
| McLoone 2012 | Targeted | Primary | WL_CB_CB | Mixed | 7-12 | 9.7 | 10 | 600 | SCAS | 41.1 | NR | NR |
| Tokolahi 2018 | Targeted | Primary | WL_OT | Mixed | 11-13 | 12.1 | 8 | 480 | MASC | 52.1 | CDI | 73.6 |
| Scholten 2016 | Targeted | Secondary | AC_Bio | Mixed | 11-15 | 13.3 | 6 | 360 | SCAS | 0.8 | NR | NR |
| Fitzgerald 2016 | Targeted | Secondary | AC_BM | Mixed | 15-18 | 15.9 | 4 | . | SCARED | 37.4 | RCADS | 12.2 |
| Dobson 2010 | Targeted | Secondary | AC_CB | Mixed | 13-18 | 15.3 | 15 | 675 | BAI | 31.3 | CES-D | 32.1 |
| Rice 2008 | Targeted | Secondary | AC_CB_MR | Mixed | 10-18 | 13.2 | 16 | 560 | MASC | 53.7 | NR | NR |
| Osborn 2020b | Targeted | Secondary | AC_PP | Mixed | 14-17 | 15.7 | 4 | 240 | GAD7 | 13.0 | PHQ8 | 13.2 |
| Osborn 2021 | Targeted | Secondary | AC_PP | Mixed | 13-18 | 15.5 | 4 | 240 | GAD7 | 13.3 | PHQ8 | 12.7 |
| Clarke 1995 | Targeted | Secondary | NI_CB | Mixed | 14-16 | 15.3 | 15 | 675 | NR | NR | CES-D | 22.9 |
| Cova 2011 | Targeted | Secondary | NI_CB | Female | 14-15 | . | 11 | 990 | BAI | 13.8 | BDI | 17.3 |
| Hamdani 2022 | Targeted | Secondary | NI_CB | Mixed | 13-15 | 13.6 | 7 | 630 | NR | NR | PHQ9 | 7.4 |
| Puskar 2003 | Targeted | Secondary | NI_CB | Mixed | 14-18 | 16.0 | 10 | 450 | NR | NR | RADS | 70.3 |
| Sportel 2013 | Targeted | Secondary | NI_CB_BM | Mixed | 12-15 | 14.1 | 20 | 900 | RCADS-SocA | 13.4 | NR | NR |
| Gillham 2012 | Targeted | Secondary | NI_CB_CB | Mixed | 10-15 | . | 10 | 900 | RCMAS | 10.8 | CDI | 10.5 |
| Sheffield 2006b | Targeted | Secondary | NI_CB_CB_CB | Mixed | 13-15 | 14.3 | 8 | 380 | SCAS | 38.0 | CDI | 22.0 |
| Briere 2019* | Targeted | Secondary | PE_CB | Mixed | 14-18 | 15.5 | 6 | 360 | SCAS | 2.6 | CES-D | 1.6 |
| de Jonge-Heesen 2021* | Targeted | Secondary | PE_CB | Mixed | 12-16 | 13.6 | 8 | 480 | STAI | 42.6 | CDI | 15.9 |
| Rohde 2014 | Targeted | Secondary | PE_CB | Mixed | 13-19 | 15.5 | 6 | 360 | NR | NR | K-SADS | 1.4 |
| Stice 2008 | Targeted | Secondary | PE_PS_CB | Mixed | 14-19 | 15.6 | 6 | 360 | NR | NR | BDI | 19.6 |
| Kiselica 1994 | Targeted | Secondary | PS_CB | Mixed | 14-15 | . | 8 | 480 | STAI | 45.3 | NR | 8.6 |
| McCarty 2013 | Targeted | Secondary | PS_CB | Mixed | 11-15 | 12.7 | 12 | 600 | NR | 61.2 | MFQ | 14.8 |
| Pile 2021 | Targeted | Secondary | PS_CB | Mixed | 16-18 | 17.1 | 4 | . | SCARED | 38.8 | MFQ | 33.3 |
| Young 2006 | Targeted | Secondary | PS_IP | Mixed | 11-16 | 13.4 | 10 | 900 | NR | NR | CES-D | 25.2 |
| Young 2010 | Targeted | Secondary | PS_IP | Mixed | 13-17 | 14.5 | 10 | 900 | NR | NR | CES-D | 26.4 |
| Young 2016 | Targeted | Secondary | PS_IP | Mixed | . | 13.5 | 11 | 450 | NR | NR | CES-D | 15.3 |
| McCarty 2011 | Targeted | Secondary | UC_CB | Mixed | . | 13.0 | 12 | . | NR | 61.2 | MFQ | 14.6 |
| Woods 2011 | Targeted | Secondary | UC_CB | Mixed | . | 14.0 | 8 | 720 | NR | NR | CDI | 24.6 |
| Livheim 2014 | Targeted | Secondary | UC_TW | Female | 12-17 | 14.6 | 8 | . | NR | NR | RADS2 | 65.2 |
| Hiebert 1989a | Targeted | Secondary | WL_Bio_MR | Mixed | 15-17 | 15.6 | 8 | 320 | STAI | 45.4 | NR | NR |
| Balle 2010 | Targeted | Secondary | WL_CB | Mixed | 11-17 | 13.6 | 6 | 270 | SCAS | 40.6 | CDI | 13.9 |
| Berry 2009 | Targeted | Secondary | WL_CB | Male | 12-15 | 13.0 | 8 | 480 | SCARED | 18.6 | CES-D-C | 18.6 |
| Congelton 1995 | Targeted | Secondary | WL_CB | Mixed | 12-14 | . | 8 | 480 | NR | NR | CDI | 49.0 |
| Topper 2017 | Targeted | Secondary | WL_CB | Mixed | 15-22 | 17.5 | 6 | 540 | MASQ | NE | BDI | 11.8 |
| Wijnhoven 2014 | Targeted | Secondary | WL_CB | Female | 11-15 | 13.3 | 8 | 400 | NR | NR | CDI | 20.9 |
| Haugland 2020 | Targeted | Secondary | WL_CB_CB | Mixed | 12-16 | 14.0 | 5-10 | 450-900 | SCAS | 42.0 | SMFQ | 11.1 |
| Poppelaars 2016 | Targeted | Secondary | WL_CB_CB_CB | Female | 11-16 | 13.4 | 8 | 480 | NR | NR | RADS | 68.8 |
| Jordans 2010 | Targeted | Secondary | WL_Mix | Mixed | 11-14 | 12.7 | 15 | 900 | SCARED5 | 4.3 | DSRS | 13.3 |

### Model fit and selection statistics: Appendix Tables 5-8

The following tables report model fit statistics for each population and outcome, for the post intervention timepoint analysis. We assessed both fixed and random effects models. Heterogeneity was evaluated by examining the posterior median between-study standard deviation (τ) and 95% Credible Intervals (CrIs) from the random effects model, and by comparing model fit of the fixed and random effects models. Model fit was measured by the posterior mean of residual deviance. In addition, we examined the Deviance Information Criterion (DIC), which penalises model fit with model complexity. Differences of ≥ 5 points for posterior mean residual deviance and DIC were considered meaningful, with lower values preferred (Spigelhalter et al, 2002). Inconsistency was assessed by comparing the goodness of fit of a model assuming consistency with one allowing for inconsistency (i.e. a model which provides effect estimates based on direct evidence only). A common between-study variance was also assumed for both the consistency and inconsistency models.

Spiegelhalter DJ, Best NG, Carlin BP, Van Der Linde A. Bayesian measures of model complexity and fit. *Journal of the Royal Statistical Society: Series B (Statistical Methodology)* 2002;64:583-639.

**Priors used for all models:**

Between study standard deviation: Uniform(0,5)

Intervention effect: Normal (0, 1000)

Convergence for full NMA and control group analyses was satisfactory at 100,000 iterations on 2 chains observed using BGR diagnostic in WinBUGS.

| **Universal Depression** | **#trt** | **#Studies** | **Data- points** | **Totresdev** | **pD** | **DIC** | **SD (95% CrIs)** | |
| --- | --- | --- | --- | --- | --- | --- | --- | --- |
| Secondary educational setting only (FE consistency) | 14 | 48 | 114 | 186.4 | 61.0 | 317.7 | NA | |
| Secondary educational setting only (RE consistency) |  |  |  | 117.0 | 89.6 | 276.9 | 0.12 (0.08 to 0.18) | |
| Secondary educational setting only (RE inconsistency) |  |  |  | 121.7 | 89.8 | 281.9 | 0.11 (0.07 to 0.16) | |
| Primary educational setting only (FE consistency) | 10 | 16 | 38 | 77.3 | 25.0 | 158.4 | NA | |
| Primary educational setting only (RE consistency) |  |  |  | 37.9 | 34.7 | 128.8 | 0.31 (0.18 to 0.55) | |
| Primary educational setting only (RE inconsistency) |  |  |  | 38.0 | 34.4 | 128.5 | 0.28 (0.15 to 0.50) | |
| Combined setting all interventions (RE cons) | 15 | 64 | 152 | 154.9 | 120.5 | 401.8 | 0.15 (0.11 to 0.20) | |
| Combined setting all interventions (RE incons) |  |  |  | 156.9 | 123.6 | 406.9 | 0.15 (0.11 to 0.20) | |
| Combined setting all interventions (RE cons meta-regression*) |  |  |  | 154.9 | 121.0 | 402.3 | 0.15 (0.11 to 0.20) | |
| Combined setting CB control NMA (6 distinct controls) (FE cons) | 7 | 43 | 97 | 201.8 | 48.9 | 332.5 | NA | |
| Combined setting CB control NMA (6 distinct controls) (RE cons) |  |  |  | 99.5 | 79.3 | 260.6 | 0.17 (0.12 to 0.24) | |
| Combined setting CB control NMA (6 distinct controls) (RE incons) |  |  |  | 99.3 | 79.6 | 260.7 | 0.16 (0.11 to 0.23) | |
| Combined setting CB only (control group to approx. WS) (RE cons) | 4 |  |  | 99.7 | 77.7 | 259.2 | 0.17 (0.12 to 0.23) | |
| Combined setting CB only (control group to approx. Stockings) (RE cons) | 3 |  |  | 100.5 | 77.3 | 259.5 | 0.16 (0.12 to 0.23) | |
| *β= 0.07 (95% CrI -0.09 to 0.23)  #trt: number of interventions and controls contributing to each analysis. #Studies: number of studies contributing to each analysis.  Model: FE = fixed effect, RE = random effects. Cons: consistency model. Incon: inconsistency model.  Datapoints: number of effective datapoints in model  Totresdev: posterior mean total residual deviance  DIC: deviance information criterion  pD: effective parameters  SD: between study heterogeneity parameter (τ) and 95% Credible Intervals | | | | | | | |  |
| **Universal Anxiety** | **#trt** | **#Studies** | **Data- points** | **Totresdev** | **pD** | **DIC** | **SD (95% CrIs)** | |
| Secondary educational setting only (FE consistency) | 8 | 29 | 65 | 84.0 | 35.9 | 168.9 | NA | |
| Secondary educational setting only (RE consistency) |  |  |  | 75.9 | 43.5 | 168.4 | 0.06 (0.00 to 0.14) | |
| Secondary educational setting only (RE inconsistency) |  |  |  | 78.4 | 45.8 | 173.2 | 0.05 (0.00 to 0.13) | |
| Primary educational setting only (FE consistency) | 10 | 16 | 18 | 53.4 | 24.9 | 166.1 | NA | |
| Primary educational setting only (RE consistency) |  |  |  | 44.03 | 32.2 | 164 | 0.12 (0.01 to 0.27) | |
| Primary educational setting only (RE inconsistency) |  |  |  | 46.3 | 32.7 | 166.8 | 0.11 (0.00 to 0.27) | |
| Combined setting all interventions (RE cons) | 10 | 47 | 106 | 123.0 | 67.6 | 327.3 | 0.06 (0.00 to 0.13) | |
| Combined setting all interventions (RE incons) |  |  |  | 127.7 | 69.8 | 334.3 | 0.05 (0.00 to 0.27) | |
| Combined setting all interventions (RE cons meta-regression*) |  |  |  | 121.7 | 69.4 | 327.9 | 0.07 (0.00 to 0.13) | |
| Combined setting CB control NMA (6 distinct controls) (FE cons) | 6 | 37 | 81 | 103.9 | 41.9 | 282.4 | NA | |
| Combined setting CB control NMA (6 distinct controls) (RE cons) |  |  |  | 93.5 | 51.6 | 281.5 | 0.06 (0.00 to 0.14) | |
| Combined setting CB control NMA (6 distinct controls) (RE incons) |  |  |  | 96.6 | 52.3 | 285.4 | 0.05 (0.00 to 0.13) | |
| Combined setting CB only (control group to approx. WS) (RE cons) | 4 |  |  | 94.3 | 48.0 | 278.8 | 0.05 (0.00 to 0.12) | |
| Combined setting CB only (control group to approx. Stockings) (RE cons) | 3 |  |  | 94.1 | 46.6 | 277.2 | 0.04 (0.00 to 0.11) | |
| *β= 0.05 (95% CrI -0.08 to 0.18)  #trt: number of interventions and controls contributing to each analysis. #Studies: number of studies contributing to each analysis.  Model: FE = fixed effect, RE = random effects. Cons: consistency model. Incon: inconsistency model.  Datapoints: number of effective datapoints in model  Totresdev: posterior mean total residual deviance  DIC: deviance information criterion  pD: effective parameters  SD: between study heterogeneity parameter (τ) and 95% Credible Intervals | | | | | | | |  |
| **Targeted Depression** | **#trt** | **#Studies** | **Data- points** | **Totresdev** | **pD** | **DIC** | **SD (95% CrIs)** | |
| Secondary educational setting only (FE consistency) | 11 | 30 | 64 | 177.8 | 40.0 | 300.6 | NA | |
| Secondary educational setting only (RE consistency) |  |  |  | 69.5 | 62.0 | 214.3 | 0.39 (0.27 to 0.56) | |
| Secondary educational setting only (RE inconsistency) |  |  |  | 70.8 | 62.3 | 215.8 | 0.38 (0.26 to 0.56) | |
| Primary educational setting only (FE consistency) | 5 | 6 | 12 | 17.5 | 10.0 | 44.1 | NA | |
| Primary educational setting only (RE consistency) |  |  |  | 12.3 | 12.0 | 40.9 | 0.60 (0.07 to 3.79) | |
| Primary educational setting only (RE inconsistency) |  |  |  | 12.3 | 12.0 | 40.9 | 0.60 (0.07 to 3.81) | |
| Combined setting all interventions (RE cons) | 12 | 36 | 80 | 82.2 | 72.3 | 253.9 | 0.36 (0.25 to 0.51) | |
| Combined setting all interventions (RE incons) |  |  |  | 83.8 | 72.5 | 255.8 | 0.35 (0.24 to 0.50) | |
| Combined setting all interventions (RE cons meta-regression*) |  |  |  | 82.1 | 72.7 | 254.3 | 0.37 (0.26 to 0.52) | |
| Combined setting CB control NMA (6 distinct controls) (FE cons) | 7 | 28 | 64 | 163.0 | 33.9 | 275.8 | NA | |
| Combined setting CB control NMA (6 distinct controls) (RE cons) |  |  |  | 66.6 | 56.9 | 202.3 | 0.35 (0.24 to 0.50) | |
| Combined setting CB control NMA (6 distinct controls) (RE incons) |  |  |  | 68.3 | 57.0 | 204.1 | 0.34 (0.22 to 0.50) | |
| Combined setting CB only (control group to approx. WS) (RE cons) | 4 |  |  | 66.9 | 56.6 | 202.6 | 0.37 (0.26 to 0.53) | |
| Combined setting CB only (control group to approx. Stockings) (RE cons) | 3 |  |  | 66.3 | 56.7 | 201.7 | 0.37 (0.27 to 0.52) | |
| *β= 0.06 (95%CrI -0.60 to 0.72) [WL as reference]  #trt: number of interventions and controls contributing to each analysis. #Studies: number of studies contributing to each analysis.  Model: FE = fixed effect, RE = random effects. Cons: consistency model. Incon: inconsistency model.  Datapoints: number of effective datapoints in model  Totresdev: posterior mean total residual deviance  DIC: deviance information criterion  pD: effective parameters  SD: between study heterogeneity parameter (τ) and 95% Credible Intervals | | | | | | | |  |
| **Targeted Anxiety** | **#trt** | **#Studies** | **Data- points** | **Totresdev** | **pD** | **DIC** | **SD (95% CrIs)** | |
| Secondary educational setting only (FE consistency) | 10 | 20 | 46 | 47.6 | 29.0 | 136.1 | NA | |
| Secondary educational setting only (RE consistency) |  |  |  | 45.9 | 32.1 | 137.6 | 0.05 (0.00 to 0.18) | |
| Secondary educational setting only (RE inconsistency) |  |  |  | 47.4 | 35.1 | 142.1 | 0.05 (0.00 to 0.19) | |
| Primary educational setting only (FE consistency) | 6 | 12 | 25 | 54.8 | 17.0 | 90.6 | NA | |
| Primary educational setting only (RE consistency) |  |  |  | 25.9 | 24.2 | 68.8 | 0.42 (0.21 to 0.89) | |
| Primary educational setting only (RE inconsistency) |  |  |  | 25.8 | 24.5 | 68.9 | 0.47 (0.23 to 1.07) | |
| Combined setting all interventions (RE cons) | 11 | 33 | 71 | 79.4 | 56.7 | 214.4 | 0.16 (0.04 to 0.28) | |
| Combined setting all interventions (RE incons) |  |  |  | 81.0 | 56.9 | 219.2 | 0.16 (0.03 to 0.31) | |
| Combined setting all interventions (RE cons meta-regression*) |  |  |  | 78.9 | 57.9 | 215.1 | 0.17 (0.04 to 0.29) | |
| Combined setting CB control NMA (6 distinct controls) (FE cons) | 6 | 24 | 52 | 83.8 | 28.9 | 195.1 | NA | |
| Combined setting CB control NMA (6 distinct controls) (RE cons) |  |  |  | 58.9 | 43.1 | 184.4 | 0.19 (0.05 to 0.34) | |
| Combined setting CB control NMA (6 distinct controls) (RE incons) |  |  |  | 61.0 | 42.7 | 186.1 | 0.18 (0.03 to 0.33) | |
| Combined setting CB only (control group to approx. WS) (RE cons) | 4 |  |  | 56.5 | 45.1 | 183.9 | 0.26 (0.15 to 0.41) | |
| Combined setting CB only (control group to approx. Stockings) (RE cons) | 3 |  |  | 55.3 | 46.1 | 183.7 | 0.30 (0.19 to 0.45) | |

*β= 0.05 (95% CrI -0.25 to 0.35) [WL as reference]

#trt: number of interventions and controls contributing to each analysis. #Studies: number of studies contributing to each analysis.

Model: FE = fixed effect, RE = random effects. Cons: consistency model. Incon: inconsistency model.

Datapoints: number of effective datapoints in model

Totresdev: posterior mean total residual deviance

DIC: deviance information criterion

pD: effective parameters

SD: between study heterogeneity parameter (τ) and 95% Credible Intervals

### CINeMA assessment: judgements and reasons for downgrading: Appendix Tables 9-16

|  |  |  |  |  |  |  |  |
| --- | --- | --- | --- | --- | --- | --- | --- |
| **Secondary universal depression (NMA results reported in Table 1a)** | | | | | | | |
| **Comparison** | **Within-study bias** | **Reporting bias** | **Indirectness** | **Imprecision** | **Heterogeneity** | **Incoherence** | **Reason(s) for downgrading** |
| BT:UC | Some concerns | Low risk | No concerns | Major concerns | No concerns | No concerns | RoB, Imprecision |
| CB:UC | Some concerns | Low risk | No concerns | No concerns | Major concerns | Some concerns | RoB, Heterogeneity, Inconsistency |
| IP:UC | Some concerns | Low risk | No concerns | Major concerns | No concerns | No concerns | RoB, Imprecision |
| MR:UC | Some concerns | Low risk | No concerns | Some concerns | Some concerns | No concerns | RoB, Imprecision, Heterogeneity |
| PE:UC | Some concerns | Low risk | No concerns | Some concerns | Some concerns | No concerns | RoB, Imprecision, Heterogeneity |
| PS:UC | Some concerns | Low risk | No concerns | Some concerns | Some concerns | Some concerns | RoB, Imprecision, Heterogeneity, Inconsistency |
| TW:UC | Some concerns | Low risk | No concerns | Some concerns | Some concerns | No concerns | RoB, Imprecision, Heterogeneity |
| AC:UC | Some concerns | Low risk | No concerns | Some concerns | Some concerns | No concerns | RoB, Imprecision, Heterogeneity |
| IP+CB:UC | Some concerns | Low risk | No concerns | Some concerns | Some concerns | No concerns | RoB, Imprecision, Heterogeneity |
| NI:UC | Some concerns | Low risk | No concerns | Some concerns | Some concerns | No concerns | RoB, Imprecision, Heterogeneity |
| PP:UC | Some concerns | Some concerns | No concerns | Major concerns | No concerns | No concerns | RoB, Imprecision |
| UC:WL | Some concerns | Low risk | No concerns | No concerns | Major concerns | No concerns | RoB, Heterogeneity |
| **Secondary targeted depression (NMA results reported in Table 1a)** | | | |  |  |  |  |
| **Comparison** | **Within-study bias** | **Reporting bias** | **Indirectness** | **Imprecision** | **Heterogeneity** | **Incoherence** | **Reason(s) for downgrading** |
| AC:NI | Some concerns | Low risk | No concerns | No concerns | Major concerns | No concerns | RoB, Heterogeneity |
| BM:NI | Some concerns | Low risk | No concerns | Some concerns | Some concerns | No concerns | RoB, Imprecision, Heterogeneity |
| CB:NI | Some concerns | Low risk | No concerns | No concerns | Major concerns | No concerns | RoB, Heterogeneity |
| IP:NI | Some concerns | Low risk | Some concerns | Major concerns | No concerns | No concerns | RoB, Indirectness, Heterogeneity |
| PE:NI | Some concerns | Low risk | No concerns | Major concerns | No concerns | No concerns | RoB, Imprecision |
| PP:NI | Some concerns | Some concerns | No concerns | No concerns | No concerns | No concerns | RoB, Publication bias |
| PS:NI | Some concerns | Low risk | No concerns | Major concerns | No concerns | No concerns | RoB, Imprecision |
| TW:NI | Some concerns | Low risk | Some concerns | No concerns | No concerns | No concerns | RoB, Indirectness |
| UC:NI | Some concerns | Low risk | No concerns | Major concerns | No concerns | No concerns | RoB, Imprecision |
| WL:NI | Some concerns | Low risk | No concerns | Major concerns | No concerns | No concerns | RoB, Imprecision |
| **Primary universal depression (NMA results reported in Table 1a)** | | | |  |  |  |  |
| **Comparison** | **Within-study bias** | **Reporting bias** | **Indirectness** | **Imprecision** | **Heterogeneity** | **Incoherence** | **Reason(s) for downgrading** |
| AC:UC | Some concerns | Low risk | No concerns | Major concerns | No concerns | No concerns | RoB, Imprecision |
| BM:UC | Some concerns | Low risk | No concerns | Major concerns | No concerns | No concerns | RoB, Imprecision |
| CB:UC | Some concerns | Low risk | No concerns | No concerns | Major concerns | No concerns | RoB, Heterogeneity |
| EX:UC | Some concerns | Low risk | No concerns | Some concerns | Some concerns | No concerns | RoB, Imprecision, Heterogeneity |
| MR:UC | Some concerns | Low risk | No concerns | Major concerns | No concerns | No concerns | RoB, Imprecision |
| NI:UC | Some concerns | Low risk | No concerns | Major concerns | No concerns | No concerns | RoB, Imprecision |
| PP:UC | Some concerns | Some concerns | No concerns | Some concerns | Some concerns | No concerns | RoB, Imprecision, Heterogeneity |
| UC:WL | Some concerns | Low risk | No concerns | Major concerns | No concerns | No concerns | RoB, Imprecision |
| **Primary targeted depression (NMA results reported in Table 1a)** | | | |  |  |  |  |
| **Comparison** | **Within-study bias** | **Reporting bias** | **Indirectness** | **Imprecision** | **Heterogeneity** | **Incoherence** | **Reason(s) for downgrading** |
| AC:WL | Some concerns | Low risk | No concerns | No concerns | Major concerns | No concerns | RoB, Heterogeneity |
| CB:WL | Some concerns | Low risk | No concerns | No concerns | Major concerns | No concerns | RoB, Heterogeneity |
| OT:WL | No concerns | Low risk | No concerns | Major concerns | No concerns | No concerns | Imprecision |
| PS:WL | Some concerns | Low risk | No concerns | No concerns | Major concerns | No concerns | RoB, Heterogeneity |
| **Secondary universal anxiety (NMA results reported in Table 1b)** | | | | |  |  |  |
| **Comparison** | **Within-study bias** | **Reporting bias** | **Indirectness** | **Imprecision** | **Heterogeneity** | **Incoherence** | **Reason(s) for downgrading** |
| AC:UC | Some concerns | Low risk | No concerns | Some concerns | Some concerns | No concerns | RoB, Imprecision, Heterogeneity |
| CB:UC | Some concerns | Low risk | No concerns | No concerns | Major concerns | No concerns | RoB, Heterogeneity |
| MR:UC | Some concerns | Low risk | No concerns | Some concerns | Some concerns | No concerns | RoB, Imprecision, Heterogeneity |
| TW:UC | Some concerns | Low risk | No concerns | No concerns | Major concerns | No concerns | RoB, Heterogeneity |
| NI:UC | Some concerns | Low risk | No concerns | Some concerns | No concerns | No concerns | RoB, Imprecision, Heterogeneity |
| PP:UC | Some concerns | Some concerns | No concerns | Some concerns | Some concerns | No concerns | RoB, Imprecision, Heterogeneity |
| UC:WL | Some concerns | Low risk | No concerns | No concerns | Major concerns | No concerns | RoB, Heterogeneity |
| **Secondary targeted anxiety (NMA results reported in Table 1b)** | | | |  |  |  |  |
| **Comparison** | **Within-study bias** | **Reporting bias** | **Indirectness** | **Imprecision** | **Heterogeneity** | **Incoherence** | **Reason(s) for downgrading** |
| AC:NI | Some concerns | Low risk | No concerns | Some concerns | Some concerns | No concerns | RoB, Imprecision, Heterogeneity |
| BIO:NI | Some concerns | Low risk | No concerns | Major concerns | No concerns | No concerns | RoB, Imprecision |
| BM:NI | No concerns | Low risk | No concerns | Some concerns | No concerns | No concerns | RoB |
| CB:NI | Some concerns | Low risk | No concerns | Some concerns | Some concerns | No concerns | RoB, Imprecision, Heterogeneity |
| MR:NI | Some concerns | Low risk | No concerns | Major concerns | No concerns | No concerns | RoB, Imprecision |
| PE:NI | Some concerns | Low risk | No concerns | Major concerns | No concerns | No concerns | RoB, Imprecision |
| PP:NI | Some concerns | Low risk | No concerns | No concerns | No concerns | No concerns | RoB |
| PS:NI | Some concerns | Low risk | No concerns | No concerns | No concerns | No concerns | RoB |
| UC:NI | No concerns | Low risk | Some concerns | Major concerns | No concerns | No concerns | Indirectness, Imprecision |
| WL:NI | Some concerns | Low risk | No concerns | No concerns | No concerns | No concerns | RoB |
| **Primary universal anxiety (NMA results reported in Table 1b)** | | | |  |  |  |  |
| **Comparison** | **Within-study bias** | **Reporting bias** | **Indirectness** | **Imprecision** | **Heterogeneity** | **Incoherence** | **Reason(s) for downgrading** |
| AC:UC | Some concerns | Low risk | No concerns | Major concerns | No concerns | No concerns | RoB, Imprecision |
| BM:UC | Some concerns | Low risk | No concerns | Major concerns | No concerns | No concerns | RoB, Imprecision |
| CB:UC | Some concerns | Low risk | No concerns | No concerns | Major concerns | No concerns | RoB, Heterogeneity |
| MR:UC | Some concerns | Low risk | No concerns | Major concerns | No concerns | No concerns | RoB, Imprecision |
| NI:UC | Some concerns | Low risk | No concerns | Major concerns | No concerns | No concerns | RoB, Imprecision |
| PE:UC | Some concerns | Low risk | No concerns | Major concerns | No concerns | No concerns | RoB, Imprecision |
| UC:WL | Some concerns | Low risk | No concerns | Some concerns | Some concerns | No concerns | RoB, Imprecision, Heterogeneity |
| **Primary targeted anxiety (NMA results reported in Table 1b)** | | | |  |  |  |  |
| **Comparison** | **Within-study bias** | **Reporting bias** | **Indirectness** | **Imprecision** | **Heterogeneity** | **Incoherence** | **Reason(s) for downgrading** |
| AC:WL | Some concerns | Low risk | No concerns | Some concerns | Some concerns | No concerns | RoB, Imprecision, Heterogeneity |
| BIO:WL | Some concerns | Low risk | No concerns | Some concerns | Some concerns | No concerns | RoB, Imprecision, Heterogeneity |
| CB:WL | Some concerns | Low risk | No concerns | No concerns | Major concerns | No concerns | RoB, Heterogeneity |
| OT:WL | No concerns | Low risk | No concerns | Major concerns | No concerns | No concerns | RoB, Imprecision |
| PS:WL | Some concerns | Low risk | No concerns | No concerns | Major concerns | No concerns | RoB, Heterogeneity |

### Full NMA findings by setting and population: update compared with 2019 review. Appendix Tables 17-24

| **Secondary -Universal-Depression** | | | |  | |  | | |  |
| --- | --- | --- | --- | --- | --- | --- | --- | --- | --- |
|  | **Update NMA (RE)**  **SMD (95% CrI)** | | | **2019 NMA(RE)**  **SMD (95% CrI)** | | | | | |
| Usual | Reference | | | Reference | | | | | |
| Waiting list | 0.01 | | (-0.13 , 0.15) | 0.00 | | | | (-0.19 , 0.19) | |
| No intervention | 0.06 | | (-0.09 , 0.23) | 0.03 | | | | (-0.15 , 0.21) | |
| Attention control | 0.13 | | (-0.12 , 0.38) | 0.07 | | | | (-0.12 , 0.25) | |
| CB | -0.01 | | (-0.10 , 0.07) | -0.04 | | | | (-0.16 , 0.08) | |
| Third wave | -0.03 | | (-0.17 , 0.11) | -0.04 | | | | (-0.21 , 0.14) | |
| CB+IP | -0.16 | | (-0.40 , 0.08) | -0.19 | | | | (-0.45 , 0.08) | |
| IP | -0.01 | | (-0.30 , 0.27) | -0.03 | | | | (-0.36 , 0.29) | |
| Education | -0.13 | | (-0.44 , 0.17) | -0.13 | | | | (-0.49 , 0.22) | |
| Behavioural | -0.02 | | (-0.36 , 0.33) | -0.02 | | | | (-0.40 , 0.36) | |
| Mindfulness | -0.04 | | (-0.20 , 0.12) | - | | | | - | |
| Positive Psych. | 0.07 | | (-0.24 , 0.37) | - | | | | - | |
| Exercise | -0.13 | | (-0.45 , 0.19) | - | | | | - | |
| Supportive | 0.08 | | (-0.11 , 0.26) | - | | | | - | |
| **SD** | 0.12 | | (0.08, 0.18) | 0.15 | | | (0.10, 0.22) | | |
| **N studies** |  | 48 | |  | 34 | | | | |

| **Primary-Universal-Depression** | | | | | | | | |  | | | | |  |  |
| --- | --- | --- | --- | --- | --- | --- | --- | --- | --- | --- | --- | --- | --- | --- | --- |
|  | **Update NMA (RE)**  **SMD (95% CrI)** | | | | | | | | | | | **2019 NMA(RE)**  **SMD (95% CrI)** | | | |
| Usual curriculum | Reference | | | | | | | | | | | Reference | | | |
| Waiting list | -0.11 | | | (-0.75 , 0.48) | | | | | | | | -0.09 | | (-0.77 , 0.54) | |
| No intervention | 0.02 | | | (-0.54 , 0.57) | | | | | | | | 0.13 | | (-0.40 , 0.66) | |
| Attention control | -0.16 | | | (-0.87 , 0.53) | | | | | | | | -0.07 | | (-0.80 , 0.63) | |
| CB | -0.16 | | | (-0.42 , 0.10) | | | | | | | | -0.13 | | (-0.44 , 0.18) | |
| Behaviour therapy | -0.03 | | | (-0.68 , 0.61) | | | | | | | | -0.10 | | (-1.04 , 0.81) | |
| Exercise | -0.20 | | | (-1.13 , 0.69) | | | | | | | | - | | - | |
| Mindfulness | 0.19 | | | (-0.51 , 0.89) | | | | | | | | - | | - | |
| BM | 0.16 | | | (-0.75 , 1.07) | | | | | | | | - | | - | |
| Positive Psych. | -0.25 | | | (-1.22 , 0.71) | | | | | | | | - | | - | |
| **SD** | 0.31 | | | (0.18, 0.55) | | | | | | | | 0.32 | | (0.18, 0.59) | |
| **N studies** |  | | | 16 | | | | | | | |  | | 12 | |
| **Secondary -Targeted - Depression** | | | | | | |  | | | | | |  | | |
|  | | **Update NMA (RE)**  **SMD (95% CrI)** | | | | | | | | | | **2019 NMA(RE)**  **SMD (95% CrI)** | | | |
| No intervention | | Reference | | | | | | | | | | Reference | | | |
| Waiting list | | 0.15 | (-0.32, 0.60) | | | | | | | | | 0.20 | (-0.31 , 0.70) | | |
| Usual curriculum | | 0.00 | (-0.75, 0.77) | | | | | | | | | 0.04 | (-0.72 , 0.82) | | |
| Attention control | | -0.85 | (-1.85, 0.14) | | | | | | | | | -0.81 | (-1.83 , 0.20) | | |
| Supportive | | 0.22 | (-0.36, 0.80) | | | | | | | | | 0.02 | (-0.64 , 0.67) | | |
| CB | | -0.25 | (-0.60, 0.08) | | | | | | | | | -0.22 | (-0.59 , 0.14) | | |
| Third Wave | | -3.76 | (-4.98, -2.53) | | | | | | | | | -3.74 | (-4.91 , -2.58) | | |
| IP | | -0.46 | (-1.25, 0.32) | | | | | | | | | -0.65 | (-1.51 , 0.17) | | |
| BM | | -0.94 | (-2.25, 0.36) | | | | | | | | | -0.90 | (-2.22 , 0.42) | | |
| Exercise | | - | - | | | | | | | | | -0.28 | (-1.14 , 0.58) | | |
| Education | | 0.04 | (-0.49, 0.55) | | | | | | | | | 0.11 | (-0.51 , 0.72) | | |
| PP | | -1.15 | (-2.34, 0.01) | | | | | | | | | - | - | | |
| **SD** | | 0.39 | (0.27, 0.56) | | | | | | | | | 0.38 | (0.25, 0.58) | | |
| **N studies** | |  | 30 | | | | | | | | |  | 24 | | |
| **Primary- Targeted- Depression** | | | | | | | |  | | | | |  |  |  |
|  | **Update NMA (RE)**  **SMD (95% CrI)** | | | | | | | | | | | **2019 NMA(RE)**  **SMD (95% CrI)** | | | |
| Waiting list | Reference | | | | | | | | | | | Reference | | | |
| Attention control | -0.72 | | (-3.54 , 2.07) | | | | | | | | | -0.72 | (-3.56, 2.10) | | |
| CB | -0.48 | | (-2.48 , 1.50) | | | | | | | | | -0.48 | (-2.49, 1.50) | | |
| Occ. Therapy | -0.10 | | (-2.91 , 2.72) | | | | | | | | | -0.10 | (-2.94, 2.71) | | |
| Supportive | -0.29 | | (-3.73 , 3.15) | | | | | | | | | - | - | | |
| **SD** | 0.60 | | (0.07, 3.79) | | | | | | | | | 0.60 | (0.08, 3.80) | | |
| **N studies** |  | | 6 | | | | | | | | |  | 5 | | |
| **Secondary- Universal-Anxiety** | | | | | | | | | |  | | | | |  |
|  | **Update NMA (RE)**  **SMD (95% CrI)** | | | | | | | | | | | **2019 NMA(RE)**  **SMD (95% CrI)** | | | |
| Usual | Reference | | | | | | | | | | | Reference | | | |
| Waiting list | 0.02 | | (-0.10 , 0.14) | | | | | | | | | -0.05 | (-0.28 , 0.18) | | |
| No intervention | 0.01 | | (-0.19 , 0.20) | | | | | | | | | -0.07 | (-0.34 , 0.20) | | |
| Attention control | 0.03 | | (-0.15 , 0.21) | | | | | | | | | -0.15 | (-0.51 , 0.16) | | |
| CB | -0.04 | | (-0.14 , 0.04) | | | | | | | | | -0.15 | (-0.34 , 0.04) | | |
| Third wave | 0.03 | | (-0.08 , 0.15) | | | | | | | | | 0.03 | (-0.14 , 0.20) | | |
| Mindfulness | -0.15 | | (-0.41 , 0.10) | | | | | | | | | -0.65 | (-1.14 , -0.19) | | |
| Positive Psych. | -0.14 | | (-0.41 , 0.12) | | | | | | | | | - | - | | |
| **SD** | 0.06 | | (0.00, 0.14) | | | | | | | | | 0.11 | (0.02, 0.23) | | |
| **N studies** |  | | 29 | | | | | | | | |  | 21 | | |
| **Secondary-Targeted-Anxiety** | | | | | | | | | |  | | |  |  |  |
|  | **Update NMA (RE)**  **SMD (95% CrI)** | | | | | | | | | | | **2019 NMA(RE)**  **SMD (95% CrI)** | | | |
| No intervention | Reference | | | | | | | | | | | Reference | | | |
| Waiting list | 0.31 | | (0.13, 0.50) | | | | | | | | | 0.31 | (0.10, 0.53) | | |
| Attention control | -0.10 | | (-0.39, 0.21) | | | | | | | | | -0.09 | (-0.38, 0.22) | | |
| Supportive | 0.82 | | (0.41, 1.22) | | | | | | | | | 1.08 | (0.52, 1.64) | | |
| CB | 0.03 | | (-0.10, 0.16) | | | | | | | | | 0.03 | (-0.10, 0.17) | | |
| Biofeedback | -0.17 | | (-0.54, 0.19) | | | | | | | | | -0.17 | (-0.54, 0.21) | | |
| MR | 0.03 | | (-0.40, 0.47) | | | | | | | | | 0.03 | (-0.41, 0.48) | | |
| BM | -0.17 | | (-0.44, 0.10) | | | | | | | | | -0.17 | (-0.44, 0.12) | | |
| Exercise | - | | - | | | | | | | | | -0.47 | (-0.87, -0.09) | | |
| Education | 0.13 | | (-0.19, 0.45) | | | | | | | | | - | - | | |
| PP | -0.59 | | (-0.96, -0.22) | | | | | | | | | - | - | | |
| **SD** | 0.05 | | (0.00, 0.18) | | | | | | | | | 0.06 | (0.00, 0.21) | | |
| **N studies** |  | | 20 | | | | | | | | |  | 15 | | |
| **Primary- Universal-Anxiety** | | | | | | | |  | | | | |  |  |  |
|  | **Update NMA (RE)**  **SMD (95% CrI)** | | | | | | | | | | | **2019 NMA(RE)**  **SMD (95% CrI)** | | | |
| Usual curriculum | Reference | | | | | | | | | | | Reference | | | |
| Waiting list | -0.02 | | (-0.24 , 0.19) | | | | | | | | | 0.02 | (-0.20 , 0.23) | | |
| No intervention | 0.19 | | (-0.34 , 0.72) | | | | | | | | | 0.23 | (-0.15 , 0.60) | | |
| Attention control | -0.21 | | (-0.56 , 0.14) | | | | | | | | | -0.17 | (-0.52 , 0.17) | | |
| CB | -0.12 | | (-0.27 , 0.01) | | | | | | | | | -0.07 | (-0.23 , 0.05) | | |
| Education | -0.02 | | (-0.35 , 0.31) | | | | | | | | | - | - | | |
| Mindfulness | -0.11 | | (-0.80 , 0.57) | | | | | | | | | - | - | | |
| BM | -0.10 | | (-0.45 , 0.22) | | | | | | | | | - | - | | |
| **SD** | 0.12 | | (0.01, 0.27) | | | | | | | | | 0.10 | (0.01, 0.26) | | |
| **N studies** |  | | 18 | | | | | | | | |  | 15 | | |
| **Primary- Targeted- Anxiety** | | | | |  |  | | | | | | |  |  |  |
|  | **Update NMA (RE)**  **SMD (95% CrI)** | | | | | | | | | | **2019 NMA(RE)**  **SMD (95% CrI)** | | | | |
| Waiting list | Reference | | | | | | | | | | Reference | | | | |
| Attention control | -0.38 | | (-1.10 , 0.32) | | | | | | | | -0.38 | | (-1.10 , 0.32) | | |
| CB | -0.38 | | (-0.84 , 0.06) | | | | | | | | -0.38 | | (-0.84 , 0.06) | | |
| Occ. Therapy | 0.11 | | (-0.91 , 1.13) | | | | | | | | 0.11 | | (-0.91 , 1.13) | | |
| Biofeedback | -0.49 | | (-1.38 , 0.39) | | | | | | | | -0.49 | | (-1.39 , 0.39) | | |
| Supportive | 0.10 | | (-0.99 , 1.19) | | | | | | | | - | | - | | |
| **SD** | 0.42 | | (0.21, 0.89) | | | | | | | | 0.42 | | (0.21, 0.88) | | |
| **N studies** |  | | 12 | | | | | | | |  | | 11 | | |

### Subgroup, meta-regression and sensitivity analyses for main NMA: Appendix Tables 25-38

The following describes the approach taken to explore effect modification/ heterogeneity in the main effectiveness NMA. Subgroup and sensitivity analyses for the control group analyses are reported separately.

###### Facilitator delivering intervention: meta-regression

Interventions were delivered by internal school staff (e.g. teacher, school nurse, school counsellor) or external staff (e.g mental health professional or student, mindfulness trainer, yoga instructor, trained lay provider). External staff also included research assistants. To explore whether intervention effects were modified by person delivering the intervention we fitted a meta-regression model for internal school staff (0) vs external staff (1). We estimated the intervention effect at each value of the covariate, for each intervention, including multi-arm trials which compared the same intervention delivered by different facilitators. Within each population-setting-outcome analysis, if there were ≥2 intervention types delivered by internal or external staff (e.g. cognitive behavioral [CB] and mindfulness) a random effects NMA model was fitted, and we assumed a hierarchical model for the regression coefficient across interventions, where the regression coefficients were assumed to come from a normal distribution with mean (m.beta) and precision (tau.beta). The between studies SD was assumed to be common for each value of the covariate. We estimated a between intervention SD (sd.beta) for the covariate regression coefficients. Vague priors were specified as per the main NMA models. Where only a single intervention varied by facilitator a fixed covariate effect (as for mode of delivery) was fitted.

##### Format of delivery: meta-regression:

Interventions were categorised as being delivered face-to-face (F2F - in person/ class) or via computer/ internet (digital). To explore whether intervention effects were modified by mode of delivery we fitted a meta-regression model for e.g. CB-F2F (covariate = 0) and CB-Digital (covariate = 1). This enables us to estimate the intervention effect for both CB-F2F and CB-digital. A random effects NMA model was fitted, but the regression coefficient for the covariate was assumed a fixed effect across studies. The between studies SD was assumed to be common for CB-F2F and CB-Digital.

##### Risk of Bias: sensitivity analysis excluding studies at unclear and high risk of bias

The PROSPERO registration stated that risk of bias sensitivity analysis would be to exclude studies at high risk of bias but did not state how we would group studies at unclear risk of bias. In the 2019 and 2021 versions of the review, studies at unclear risk of bias were grouped with those at high risk, and the same approach is followed here. Of the 125 studies included in the NMA, 7 were at high risk of bias, 19 were at low risk of bias, and 118 were at unclear risk of bias. Within each population-setting-outcome analysis, studies were excluded if both the randomisation and allocation concealment domains were rated as being at unclear or high risk of bias. Studies in which one of these domains was rated as being at low risk, but the other was rated as unclear were included.

###### Summary of Findings:

The tables below report the findings from the sensitivity and subgroup analyses from the main NMA. The NMA findings for each population, setting and outcome are largely unchanged when analyses were restricted to studies at low risk of bias. Of note, however, is that the effect estimate for positive psychology vs no intervention in targeted secondary settings is no longer statistically significant when studies at unclear and high risk of bias are excluded ((Anxiety: SMD -0.53 [95% CrIs -1.11, 0.03]).

For the facilitator subgroup analysis, there is some evidence that interventions delivered by external staff are more effective than those delivered by school staff in targeted secondary settings. However, there is no evidence of subgroup effects by facilitator in other populations and settings. For delivery format, digitally delivered positive psychology interventions may be more effective for reducing depressive symptoms than in-person formats, in universal secondary settings. However, there is no evidence of subgroup effects by format in other populations and settings.

###### Secondary-Universal – Depression Appendix Tables 25 -27

All estimates are based on 100,000 burn-ins and 200,000 iterations on two chains.

Facilitator delivering intervention:

| **Intervention/ covariate** | **SMD** | **95% CrIs** | **Regression coefficient (95% CrI)** | | |
| --- | --- | --- | --- | --- | --- |
| CB school staff | -0.02 | (-0.14 to 0.10) | 0.04 (-0.09 to 0.17) | | |
| CB external | 0.02 | (-0.10 to 0.13) |  |  |  |
| 3^rd^ wave school staff | -0.06 | (-0.26 to 0.14) | 0.04 (-0.17 to 0.24) | | |
| 3^rd^ wave external | -0.02 | (-0.19 to 0.15) |  |  |  |
| MR school staff | -0.11 | (-0.34 to 0.12) | 0.00 (-0.30 to 0.26) | | |
| MR external | -0.11 | (-0.37 to 0.14) |  |  |  |
| PP school staff | 0.02 | (-0.37 to 0.40) | 0.15 (-0.13 to 0.59) | | |
| PP external | 0.16 | (-0.25 to 0.63) |  |  |  |
| PS school staff | 0.05 | (-0.27 to 0.37) | 0.06 (-0.62 to 0.78) | | |
| PS external | 0.11 | (-0.62 to 0.88) |  |  |  |
|  | | | | |  |
| **Between study SD** |  |  | m.beta: | 0.06 (-0.26 to 0.41) |  |
| 0.15 (0.10 to 0.21) |  |  | sd.beta: | 0.12 (0.01 to 0.91) |  |

Format of delivery:

| **Intervention/ covariate** | **SMD** | **95% CrIs** | **Regression coefficient (95% CrI)** |
| --- | --- | --- | --- |
| CB in person | 0.01 | (-0.08 to 0.10) | -0.07 (-0.21 to 0.07) |
| CB digital | -0.06 | (-0.19 to 0.07) |  |
| PP in person | 0.11 | (-0.20 to 0.42) | -0.53 (-0.98 to -0.08) |
| PP digital | -0.42 | (-0.90 to -0.06) |  |
| **Between study SD:**  0.12 (0.08 to 0.17) | | | |

Risk of Bias:

| **Intervention** | **SMD (95% CrI)** | |
| --- | --- | --- |
| Usual | NA | |
| Wait list | 0.13 | (-0.05 to 0.35) |
| No intervention | 0.14 | (-0.06 to 0.38) |
| Attention control | 0.29 | (0.02 to 0.58) |
| CB | 0.06 | (-0.07 to 0.21) |
| Third wave | -0.03 | (-0.17 to 0.10) |
| CB+IP | 0.11 | (-0.28 to 0.50) |
| Mindfulness | -0.01 | (-0.17 to 0.14) |
| Positive Psych. | 0.18 | (-0.14 to 0.51) |
| Supportive | 0.13 | (-0.13 to 0.42) |
| **SD** | 0.09 | (0.01, 0.18) |
| **N studies** |  | 20 |

###### Secondary - Universal – Anxiety. Appendix Tables 28-30

All estimates are based on 100,000 burn-ins and 200,000 iterations on two chains.

#### Facilitator delivering intervention:

| **Intervention/ covariate** | **SMD** | **95% CrIs** | **Regression coefficient (95% CrI)** | | |
| --- | --- | --- | --- | --- | --- |
| CB school staff | -0.04 | (-0.13 to 0.04) | -0.04 (-0.20 to 0.10) | | |
| CB external | -0.08 | (-0.25 to 0.07) |  |  |  |
| 3^rd^ wave school staff | -0.09 | (-0.31 to 0.12) | 0.18 (-0.06 to 0.44) | | |
| 3^rd^ wave external | 0.09 | (-0.05 to 0.23) |  |  |  |
| MR school staff | -0.23 | (-0.62 to 0.13) | 0.11 (-0.25 to 0.51) | | |
| MR external | -0.13 | (-0.40 to 0.14) |  |  |  |
| PP school staff | -0.21 | (-0.56 to 0.13) | 0.10 (-0.22 to 0.43) | | |
| PP external | -0.12 | (-0.41 to 0.16) |  |  |  |
|  | | | | |  |
| **Between study SD** |  |  | m.beta: | 0.09 (-0.31 to 0.52) |  |
| 0.06 (0.01 to 0.14) |  |  | sd.beta: | 0.20 (0.02 to 1.07) |  |

#### Format of delivery:

| **Intervention/ covariate** | **SMD** | **95% CrIs** | **Regression coefficient (95% CrI)** | | |
| --- | --- | --- | --- | --- | --- |
| CB in person | -0.04 | (-0.14 to 0.04) | -0.04 (-0.20 to 0.10) | | |
| CB digital | -0.08 | (-0.26 to 0.08) |  |  |  |
| 3^rd^ wave in person | -0.04 | (-0.21 to 0.14) | 0.13 (-0.09 to 0.37) | | |
| 3^rd^ wave digital | 0.09 | (-0.06 to 0.25) |  |  |  |
| MR in person | -0.22 | (-0.61 to 0.14) | 0.09 (-0.25 to 0.49) | | |
| MR digital | -0.13 | (-0.40 to 0.14) |  |  |  |
| PP in person | -0.21 | (-0.57 to 0.14) | 0.09 (-0.21 to 0.43) | | |
| PP digital | -0.12 | (-0.42 to 0.17) |  |  |  |
|  | | | | |  |
| **Between study SD** |  |  | m.beta: | 0.07 (-0.29 to 0.48) |  |
| 0.06 (0.01 to 0.15) |  |  | sd.beta: | 0.17 (0.01 to 0.99) |  |

#### Risk of Bias:

| **Intervention** | **SMD (95% CrI)** | |
| --- | --- | --- |
| **Usual** | **NA** | |
| Wait list | 0.07 | (-0.20 to 0.32) |
| No intervention | -0.02 | (-0.34 to 0.31) |
| Attention control | 0.07 | (-0.18 to 0.33) |
| CB | -0.01 | (-0.21 to 0.19) |
| Third wave | 0.03 | (-0.12 to 0.18) |
| Mindfulness | 0.10 | (-0.25 to 0.44) |
| Positive Psych. | -0.11 | (-0.44 to 0.23) |
| **SD** | 0.09 (0.01 to 0.21) | |
| **N studies** |  | 12 |

###### Depression - Targeted – Secondary Appendix Tables 31-32

All estimates are based on 100,000 burn-ins and 200,000 iterations on two chains

Facilitator delivering intervention:

| **Intervention/ covariate** | **SMD** | **95% CrIs** | **Regression coefficient (95% CrI)** |
| --- | --- | --- | --- |
| CB school staff | -0.07 | (-0.42 to 0.28) | -0.34 (-0.66 to -0.01) |
| CB external | -0.40 | (-0.74 to -0.07) |  |
| Between study SD: 0.34 (0.22 to 0.50) | | | |

#### Format of delivery:

Analysis not feasible, due to insufficient variation in delivery format. 22 studies were face to face in class, 1 study was digital and 1 study combined in class and digital formats.

#### Risk of Bias

| **Intervention** | **SMD (95% CrI)** | |
| --- | --- | --- |
| No intervention | NA | |
| Waiting list | 0.28 | (-0.26 to 0.81) |
| Usual care | 0.76 | (-0.41 to 1.91) |
| Attention control | -0.83 | (-1.86 to 0.17) |
| Supportive | 0.23 | (-0.38 to 0.85) |
| CB | -0.23 | (-0.65 to 0.16) |
| Third wave | -3.00 | (-4.51 to -1.51) |
| IP | -0.44 | (-1.26 to 0.36) |
| Education | 0.04 | (-0.56 to 0.62) |
| Positive Psych. | -1.13 | (-2.33 to 0.05) |
| **SD** | 0.38 (0.24 to 0.57) | |
| **N studies** |  | 23 |

###### Anxiety - Targeted – Secondary. Appendix Tables 33-34

All estimates are based on 100,000 burn-ins and 200,000 iterations on two chains.

#### Facilitator delivering intervention:

| **Intervention/ covariate** | **SMD** | **95% CrIs** | **Regression coefficient (95% CrI)** |
| --- | --- | --- | --- |
| CB school staff | 0.005 | (-0.19 to 0.19) | -0.004 (-0.20 to -0.18) |
| CB external | -0.0002 | (-0.16 to 0.18) |  |
| Between study SD: 0.07 (0.00 to 0.22) | | | |

#### Format of delivery

Analysis not feasible, due to insufficient variation in delivery format within intervention type. Only biofeedback varies by format – 1 study uses digital and 1 study uses an in-person format.

#### Risk of Bias

| **Intervention** | **SMD (95% CrI)** | |
| --- | --- | --- |
| No intervention | NA | |
| Waiting list | 0.31 | (0.09 to 0.56) |
| Attention control | -0.03 | (-0.54 to 0.48) |
| Supportive | 0.52 | (-0.05 to 1.09) |
| CB | 0.01 | (-0.15 to 0.17) |
| Biofeedback | -0.06 | (-0.67 to 0.55) |
| Education | 0.09 | (-0.32 to 0.50) |
| Positive Psych. | -0.53 | (-1.11 to 0.03) |
| **SD** | 0.38 (0.24 to 0.57) | |
| **N studies** |  | 13 |

###### Depression - Universal – Primary. Appendix Table 35

All estimates are based on 100,000 burn-ins and 200,000 iterations on two chains.

#### Facilitator delivering intervention

| **Intervention/ covariate** | **SMD** | **95% CrIs** | **Regression coefficient (95% CrI)** |
| --- | --- | --- | --- |
| CB school staff | -0.21 | (-0.54 to 0.12) | 0.15 (-0.32 to 0.59) |
| CB external | -0.06 | (-0.45 to 0.30) |  |
| Between study SD: 0.32 (0.18 to 0.58) | | | |

#### Format of delivery and Risk of Bias

Delivery format subgroup analysis was not possible as 11 of 12 studies were in person, and 1 used a combined digital and in person format. Risk of bias sensitivity analysis was not feasible. Only four studies were at low risk of bias and did not form a connected network.

###### Anxiety - Universal – Primary. Appendix Tables 36-37

All estimates are based on 100,000 burn-ins and 200,000 iterations on two chains.

#### Facilitator delivering intervention

| **Intervention/ covariate** | **SMD** | **95% CrIs** | **Regression coefficient (95% CrI)** |
| --- | --- | --- | --- |
| CB school staff | -0.06 | (-0.21 to 0.06) | -0.15 (-0.33 to -0.003) |
| CB external | -0.22 | (-0.41 to -0.06) |  |
| Between study SD: 0.10 (0.01 to 0.24) | | | |

#### Format of delivery

Delivery format subgroup analysis was not feasible as 17 of 18 studies were in person, and 1 used a combined digital and in person format.

#### Risk of Bias

| **Intervention** | **SMD (95% CrI)** | | |
| --- | --- | --- | --- |
| Usual curriculum | NA | | |
| Waiting list | -0.06 | | (-5.30 to 5.23) |
| CB | -0.14 | (-3.17 to 2.91) | |
| Education | -0.02 | (-4.33 to 4.24) | |
| MR | -0.11 | (-4.43 to 4.24) | |
| BM | -0.13 | (-4.14 to 3.91) | |
| **SD** | 0.87 (0.05 to 4.58) | | |
| **N studies** |  | 6 | |

###### Depression - Targeted – Primary

No feasible analyses. Five of six studies were delivered by an external expert, with 1 study unclear about facilitator. All interventions were delivered in person. Only 2 studies were at low risk of bias. NMA was not possible as these studies did not form a connected network.

###### Anxiety - Targeted – Primary. Appendix Table 38

Facilitator and format subgroup analyses not feasible. Across 12 studies, only one was delivered by school staff and one combined school and external personnel. There was no variation in delivery within intervention type.

#### Risk of Bias

Estimates are based on 100,000 burn-ins and 200,000 iterations on two chains.

| **Intervention** | **SMD (95% CrI)** | |
| --- | --- | --- |
| Waiting list | NA | |
| Attention control | 0.03 | (-1.73 to 1.70) |
| CB | 0.02 | (-1.25 to 1.27) |
| Biofeedback | -0.08 | (-1.83 to 1.60) |
| OT | 0.11 | (-1.33 to 1.56) |
| **SD** | 0.21 (0.01 to 2.54) | |
| **N studies** |  | 5 |

### Intervention rankings for main control group analysis (six distinct control groups)

|  | **Universal** | | **Targeted** | |
| --- | --- | --- | --- | --- |
|  | Depression | Anxiety | Depression | Anxiety |
| No intervention | 5.5 (2,7) | 4.3 (1,6) | 4.3 (2,7) | 2.0 (1,4) |
| Waiting list | 3.1 (1,7) | 4.3 (2,6) | 5.8 (3,7) | 4.9 (4,6) |
| Usual curriculum | 4.3 (2,7) | 4.1 (2,6) | 4.3 (1,7) | - |
| Attention control | 5.0 (1,7) | 3.0 (1,6) | 1.2 (1,3) | 2.4 (1,4) |
| Supportive | 5.0 (1,7) | - | 5.5 (3,7) | 5.9 (5,6) |
| Education | 2.5 (1,7) | 3.4 (1,6) | 4.6 (2,7) | 3.3 (1,5) |
| CB | 2.5 (1,5) | 1.9 (1,4) | 2.3 (1,4) | 2.5 (1,4) |

Table 39: posterior mean rank (95% CrI) by population and outcome

Posterior mean rank and 95% credible intervals (CrI) for each of the six comparators/controls and CB. Intervention rankings were added post-hoc, during peer review, and were not pre-specified in the review registration.

### Subgroup and sensitivity analyses for control group comparison of results from individually and cluster randomised studies. Appendix Tables 40-43

UC: usual curriculum, WL: waiting list, NI: no intervention, AC: attention control, PS: supportive, PE: education, CB: cognitive behavioural intervention. Analyses conducted combining primary and secondary educational settings.

Standardised mean difference (SMD) and 95% credible intervals (CrIs), for each pairwise comparison possible between 7 comparators/ interventions. sd= between study variability in intervention effect (heterogeneity).

###### Universal depression: comparison of results from individually and cluster randomised studies

| Comparison  (ref v active) | Design | SMD | LCrI | UCrI |
| --- | --- | --- | --- | --- |
| UC v WL | Individual | -0.47 | -1.08 | 0.11 |
|  | Cluster | -0.02 | -0.19 | 0.16 |
| UC v NI | Individual | -0.21 | -0.59 | 0.13 |
|  | Cluster | -0.06 | -0.34 | 0.22 |
| UC v AC | Individual | -0.33 | -0.89 | 0.17 |
|  | Cluster | 0.18 | -0.21 | 0.57 |
| UC v CB | Individual | -0.37 | -0.70 | -0.08 |
|  | Cluster | -0.04 | -0.13 | 0.06 |
| UC v PE | Individual | -0.31 | -0.76 | 0.08 |
|  | Cluster | -0.13 | -0.53 | 0.26 |
| UC v PS | Cluster | 0.20 | -0.16 | 0.56 |
| sd | Individual | 0.15 | 0.02 | 0.35 |
|  | Cluster | 0.17 | 0.12 | 0.25 |

###### Universal anxiety: comparison of results from individually and cluster randomised studies

| Comparison  (ref v active) | Design | SMD | LCrI | UCrI |
| --- | --- | --- | --- | --- |
| UC v WL | Individual | 0.54 | -0.30 | 1.36 |
|  | Cluster | -0.03 | -0.14 | 0.07 |
| UC v NI | Individual | 0.42 | -0.42 | 1.23 |
|  | Cluster | -0.04 | -0.24 | 0.15 |
| UC v AC | Individual | 0.12 | -0.45 | 0.68 |
|  | Cluster | -0.05 | -0.25 | 0.12 |
| UC v CB | Individual | 0.11 | -0.67 | 0.86 |
|  | Cluster | -0.07 | -0.14 | 0.00 |
| UC v PE | Cluster | -0.13 | -0.53 | 0.26 |
| sd | Individual | 0.11 | 0.01 | 0.52 |
|  | Cluster | 0.05 | 0.00 | 0.13 |

###### Targeted depression: comparison of results from individually and cluster randomised studies

| Comparison  (ref v active) | Design | SMD | LCrI | UCrI |
| --- | --- | --- | --- | --- |
| NI v WL | Individual | 0.03 | -0.57 | 0.61 |
|  | Cluster | 0.42 | -0.29 | 1.04 |
| NI v UC | Individual | -0.06 | -0.89 | 0.78 |
| NI v AC | Individual | -0.68 | -1.39 | 0.01 |
| NI v PS | Individual | 0.15 | -0.52 | 0.81 |
|  | Cluster | 0.09 | -0.87 | 0.98 |
| NI v CB | Individual | -0.33 | -0.78 | 0.10 |
|  | Cluster | -0.10 | -0.65 | 0.40 |
| NI v PE | Individual | -0.04 | -0.66 | 0.56 |
| sd | Individual | 0.40 | 0.26 | 0.63 |
|  | Cluster | 0.29 | 0.03 | 0.72 |

###### Targeted anxiety: comparison of results from individually and cluster randomised studies

| Comparison  (ref v active) | Design | SMD | LCrI | UCrI |
| --- | --- | --- | --- | --- |
| NI v WL | Individual | 0.28 | -0.21 | 0.78 |
|  | Cluster | 0.55 | 0.18 | 0.99 |
| NI v AC | Individual | -0.04 | -0.60 | 0.54 |
|  | Cluster | 0.20 | -0.33 | 0.73 |
| NI v PS | Individual | 0.79 | 0.09 | 1.49 |
|  | Cluster | 0.56 | 0.07 | 1.07 |
| NI v CB | Individual | 0.01 | -0.42 | 0.43 |
|  | Cluster | 0.07 | -0.20 | 0.36 |
| NI v PE | Individual | 0.11 | -0.55 | 0.78 |
| sd | Individual | 0.28 | 0.11 | 0.51 |
|  | Cluster | 0.11 | 0.01 | 0.46 |

### Subgroup and sensitivity analyses for control group NMA: results excluding very small studies (n<50) Appendix Tables 44-47

UC: usual curriculum, WL: waiting list, NI: no intervention, AC: attention control, PS: supportive, PE: education, CB: cognitive behavioural intervention. Analyses conducted combining primary and secondary educational settings.

Standardised mean difference (SMD) and 95% credible intervals (CrIs), for each pairwise comparison possible between 7 comparators/ interventions. sd= between study variability in intervention effect (heterogeneity). LCrI: lower credible interval. UCrI: upper credible interval.

‘Ref’: reference intervention to which all others are compared. ‘Exp’: experimental intervention. SMDs interpreted as the effect of the experimental intervention over the reference intervention. E.g for UC vs WL in the universal depression table, the SMD would be interpreted as WL reducing depressive symptoms relative to UC.

###### Universal depression: excluding studies with <50 participants

| **Comparison** (ref v exp) | **SMD** | **LCrI** | **UCrI** |
| --- | --- | --- | --- |
| UC v WL | -0.04 | -0.21 | 0.13 |
| UC v NI | 0.07 | -0.10 | 0.25 |
| UC v AC | 0.07 | -0.23 | 0.35 |
| UC v CB | -0.06 | -0.15 | 0.03 |
| UC v PE | -0.13 | -0.53 | 0.26 |
| UC v PS | 0.05 | -0.17 | 0.28 |
| sd | 0.17 | 0.12 | 0.24 |

###### Universal anxiety: excluding studies with <50 participants

| **Comparison** (ref v exp) | **SMD** | **LCrI** | **UCrI** |
| --- | --- | --- | --- |
| UC v WL | 0.00 | -0.11 | 0.09 |
| UC v NI | 0.00 | -0.18 | 0.18 |
| UC v AC | -0.04 | -0.21 | 0.11 |
| UC v CB | -0.06 | -0.14 | 0.00 |
| UC v PE | -0.02 | -0.23 | 0.19 |
| sd | 0.06 | 0.00 | 0.13 |

###### Targeted depression: excluding studies with <50 participants

| **Comparison** (ref v exp) | **SMD** | **LCrI** | **UCrI** |
| --- | --- | --- | --- |
| NI v WL | 0.17 | -0.28 | 0.62 |
| NI v UC | 0.00 | -0.72 | 0.74 |
| NI v AC | -0.50 | -1.16 | 0.16 |
| NI v PS | 0.14 | -0.36 | 0.65 |
| NI v CB | -0.25 | -0.58 | 0.07 |
| NI v PE | 0.03 | -0.47 | 0.52 |
| sd | 0.37 | 0.24 | 0.54 |

###### Targeted anxiety: excluding studies with <50 participants

| **Comparison** (ref v expt) | **SMD** | **LCrI** | **UCrI** |
| --- | --- | --- | --- |
| NI v WL | 0.23 | 0.07 | 0.40 |
| NI v AC | 0.02 | -0.21 | 0.24 |
| NI v PS | 0.52 | 0.29 | 0.76 |
| NI v CB | 0.03 | -0.09 | 0.15 |
| NI v PE | 0.13 | -0.17 | 0.43 |
| sd | 0.04 | 0.00 | 0.13 |

### Sensitivity analysis: moderating effect of control group type for prevention of anxiety (bottom) and depression (top). Appendix Tables 48-49

|  | **UC** | **WL** | **NI** | **AC** | **PS** | **PE** | **CB** |
| --- | --- | --- | --- | --- | --- | --- | --- |
| **UC** |  | 0.05 | 0.07 | 0.23 | 0.06 | - | 0.01 |
|  |  | *(-0.09 to 0.20)* | *(-0.08 to 0.25)* | *(0.04 to 0.44)* | *(-0.14 to 0.28)* |  | *(-0.08 to 0.10)* |
| **WL** | 0.03 |  | 0.02 | 0.18 | 0.02 | - | -0.04 |
|  | *(-0.24 to 0.25)* |  | *(-0.15 to 0.20)* | *(-0.04 to 0.40)* | *(-0.20 to 0.24)* |  | *(-0.16 to 0.07)* |
| **NI** | -0.06 | -0.09 |  | 0.16 | 0.00 | - | -0.06 |
|  | *(-0.43 to 0.27)* | *(-0.44 to 0.27)* |  | *(-0.08 to 0.38)* | *(-0.19 to 0.17)* |  | *(-0.21 to 0.07)* |
| **AC** | 0.03 | 0.00 | 0.09 |  | -0.17 | - | -0.22 |
|  | *(-0.24 to 0.30)* | *(-0.28 to 0.34)* | *(-0.29 to 0.51)* |  | *(-0.43 to 0.10)* |  | *(-0.41 to -0.03)* |
| **PS** | - | - | - | - |  | - | -0.05 |
|  |  |  |  |  |  |  | *(-0.25 to 0.13)* |
| **PE** | -0.02 | -0.05 | 0.04 | -0.05 | - |  | - |
|  | *(-0.33 to 0.29)* | *(-0.42 to 0.37)* | *(-0.41 to 0.53)* | *(-0.46 to 0.36)* |  |  |  |
| **CB** | -0.05 | -0.08 | 0.01 | -0.08 | - | -0.03 |  |
|  | *(-0.24 to 0.10)* | *(-0.25 to 0.10)* | *(-0.30 to 0.32)* | *(-0.35 to 0.15)* |  | *(-0.40 to 0.30)* |  |

Low risk of bias: Universal anxiety and depression

Standardised mean difference (SMD) and 95% credible intervals (CrIs), for each pairwise comparison possible between 7 comparators/ interventions. UC: usual curriculum, WL: waiting list, NI: no intervention, AC: attention control, PS: supportive, PE: education, CBI: cognitive behavioural intervention. Analysis conducted combining primary and secondary educational settings.

Studies contributing to each analysis: Universal Depression N =11; Universal Anxiety N=10.

Universal Anxiety: (blue shading, bottom left cells). The intervention in each row is ‘experimental’ and in each column is the ‘control’: e.g. the WL vs AC denotes the effect of AC (‘experimental’) over WL (‘control’). PS was not a comparator in any study contributing to the anxiety analysis.

Universal Depression: (green shading, top right cells) The intervention in each column is ‘experimental’ and in each row is the ‘control’. Six interventions/ comparators were included in the universal depression analysis. PE was not included in the low risk of bias sensitivity analysis.

### Sensitivity analysis: moderating effect of control group type for prevention of anxiety (bottom) and depression (top).

Low risk of bias: Targeted anxiety and depression

|  | **NI** | **WL** | **UC** | **AC** | **PS** | **CB** | **PE** |
| --- | --- | --- | --- | --- | --- | --- | --- |
| **NI** |  | 0.29 | 0.76 | -0.82 | 0.23 | -0.23 | 0.04 |
|  |  | *(-0.25 to 0.80)* | *(-0.38 to 1.90)* | *(-1.82 to 0.16)* | *(-0.36 to 0.83)* | *(-0.63 to 0.15)* | *(-0.54 to 0.60)* |
| **WL** | 0.25 |  | 0.47 | -1.11 | -0.06 | -0.52 | -0.25 |
|  | *(0.02 to 0.49)* |  | *(-0.64 to 1.60)* | *(-2.08 to -0.14)* | *(-0.61 to 0.53)* | *(-0.86 to -0.18)* | *(-0.78 to 0.29)* |
| **UC** | - | - |  | -1.59 | -0.53 | -0.99 | -0.72 |
|  |  |  |  | *(-2.99 to -0.18)* | *(-1.69 to 0.63)* | *(-2.06 to 0.07)* | *(-1.86 to 0.42)* |
| **AC** | -0.10 | -0.35 | - |  | 1.06 | 0.59 | 0.87 |
|  | *(-0.44 to 0.25)* | *(-0.68 to -0.02)* |  |  | *(0.05 to 2.08)* | *(-0.32 to 1.50)* | *(-0.13 to 1.86)* |
| **PS** | 0.52 | 0.27 | - | 0.61 |  | -0.46 | -0.19 |
|  | *(-0.06 to 1.10)* | *(-0.30 to 0.84)* |  | *(-0.01 to 1.24)* |  | *(-0.93 to -0.02)* | *(-0.60 to 0.37)* |
| **CB** | -0.01 | -0.26 | - | 0.09 | -0.53 |  | 0.27 |
|  | *(-0.19 to 0.17)* | *(-0.41 to -0.11)* |  | *(-0.21 to 0.38)* | *(-1.08 to 0.02)* |  | *(-0.14 to 0.68*) |
| **PE** | - | - | - | - | - | - |  |

Standardised mean difference (SMD) and 95% credible intervals (CrIs), for each pairwise comparison possible between 7 comparators/ interventions. UC: usual curriculum, WL: waiting list, NI: no intervention, AC: attention control, PS: supportive, PE: education, CB: cognitive behavioural. Analysis conducted combining primary and secondary educational settings.

Studies contributing to each analysis: Targeted Depression N=17, Targeted Anxiety Total N=10

Targeted Anxiety: (blue shading, bottom left cells). The intervention in each row is ‘experimental’ and in each column is the ‘control’: e.g. the WL vs AC comparison denotes the effect of AC (‘experimental’) over WL (‘control’). PE and PS did not contribute to the anxiety analysis.

Targeted Depression: (green shading, top right cells) The intervention in each column is ‘experimental’ and in each row is the ‘control’. Seven interventions/ comparators were included in the targeted depression analysis.

### Post hoc Scenario analyses 1 and 2 for control group NMA Appendix Tables 50-53

##### Scenario Analysis 1: to approximate Stockings and standard pairwise meta-analyses

In this analysis comparators were grouped into two comparators ‘UC, WL, NI’ and ‘AC, PS, PE’

**Universal depression (top right) and universal anxiety (bottom left)**

|  | **NI/UC/WL** | **AC/PE/PS** | **CB** |
| --- | --- | --- | --- |
| **NI/UC/WL** | - | 0.01 | -0.06 |
|  |  | *(-0.15, 0.15)* | *(-0.13, -0.001)* |
| **AC/PE/PS** | -0.03 | - | -0.06 |
|  | *(-0.15, 0.08)* |  | *(-0.22, 0.08)* |
| **CB** | -0.06 | -0.03 | - |
|  | *(-0.11, -0.02)* | *(-0.15, 0.08)* |  |

Standardised mean difference (SMD) and 95% credible intervals (CrIs), for each pairwise comparison possible between a conflated no intervention/ usual curriculum/ waiting list grouping (UC/NI/WL), an attention control, education, and supportive control grouping (AC, PS, PE) and cognitive behavioural interventions (CB).

Analysis conducted combining primary and secondary educational settings. Studies contributing to each analysis: Universal Depression n=43; Universal Anxiety n=36

Universal Anxiety: (blue shading, bottom left cells). Row is 'active' relative to column 'control'

Universal Depression: (green shading, top right cells) Column is 'active' relative to row 'control'

**Targeted depression (top right) and Targeted anxiety (bottom left)**

|  | **NI/UC/WL** | **AC/PE/PS** | **CB** |
| --- | --- | --- | --- |
| **NI/UC/WL** | - | -0.18 | -0.34 |
|  |  | *(-0.50, 0.14)* | *(-0.54, -0.15)* |
| **AC/PE/PS** | 0.01 | - | -0.16 |
|  | *(-0.29, 0.30)* |  | *(-0.41, 0.08)* |
| **CB** | -0.21 | -0.22 | - |
|  | *(-0.39, -0.04)* | *(-0.46, 0.01)* |  |

Standardised mean difference (SMD) and 95% credible intervals (CrIs), for each pairwise comparison possible between a conflated no intervention/ usual curriculum/ waiting list grouping (UC/NI/WL), an attention control, education, and supportive control grouping (AC, PS, PE) and cognitive behavioural interventions (CB).

Analysis conducted combining primary and secondary educational settings. Studies contributing to each analysis: Targeted Depression n=28, Targeted Anxiety Total n=24

Targeted Anxiety: (blue shading, bottom left cells). Row is 'active' relative to column 'control'

Targeted Depression: (green shading, top right cells) Column is 'active' relative to row 'control'

##### Scenario Analysis 2: to approximate Werner-Seidler

In this analysis separate nodes were created for (i) UC/NI, (ii) WL, and (iii) a conflated AC/PE/PS control.

**Universal depression (top right) and universal anxiety (bottom left)**

|  | **NI/UC** | **WL** | **AC/PE/PS** | **CB** |
| --- | --- | --- | --- | --- |
| **NI/UC** | - | -0.07 | -0.002 | -0.08 |
|  |  | *(-0.22, 0.08)* | *(-0.16, 0.15)* | *(-0.16, -0.01)* |
| **WL** | 0.01 | - | 0.07 | -0.01 |
|  | *(-0.09, 0.09)* |  | *(-0.14, 0.27)* | *(-0.15, 0.12)* |
| **AC/PE/PS** | -0.03 | -0.04 | - | -0.08 |
|  | *(-0.16, 0.09)* | *(-0.17, 0.10)* |  | *(-0.23, 0.08)* |
| **CB** | -0.06 | -0.07 | -0.03 | - |
|  | *(-0.13, -0.01)* | *(-0.14, -0.002)* | *(-0.15, 0.08)* |  |

Standardised mean difference (SMD) and 95% credible intervals (CrIs), for each pairwise comparison possible between a combined no intervention (NI) and usual curriculum (UC) grouping, an ‘attention/non-specific’ grouping combining attention control, supportive, education (AC/PS/PE) and cognitive behavioural intervention (CB).

Analysis conducted combining primary and secondary educational settings. Studies contributing to each analysis: Universal Depression n=43; Universal Anxiety n=36

Universal Anxiety: (blue shading, bottom left cells). Row is 'active' relative to column 'control'

Universal Depression: (green shading, top right cells). Column is 'active' relative to row 'control'

**Targeted depression (top right) and Targeted anxiety (bottom left)**

|  | **NI/UC** | **WL** | **AC/PE/PS** | **CB** |
| --- | --- | --- | --- | --- |
| **NI/UC** | - | 0.17 | -0.09 | -0.25 |
|  |  | *(-0.24, 0.56)* | *(-0.48, 0.29)* | *(-0.55, 0.03)* |
| **WL** | 0.39 | - | -0.26 | -0.42 |
|  | *(0.06, 0.73)* |  | *(-0.62, 0.11)* | *(-0.69, -0.15)* |
| **AC/PE/PS** | 0.26 | -0.12 | - | -0.16 |
|  | *(-0.08, 0.61)* | *(-0.42, 0.17)* |  | *(-0.41, 0.08)* |
| **CB** | 0.04 | -0.34 | -0.22 | - |
|  | *(-0.23, 0.32)* | *(-0.55, -0.15)* | *(-0.43, -0.01)* |  |

Standardised mean difference (SMD) and 95% credible intervals (CrIs), for each pairwise comparison possible between a combined no intervention (NI) and usual curriculum (UC) grouping, a combined attention control, supportive, and education control (AC/PS/PE) and cognitive behavioural intervention (CB).

Analysis conducted combining primary and secondary educational settings. Studies contributing to each analysis: Targeted Depression n=28, Targeted Anxiety Total n=24

Targeted Anxiety: (blue shading, bottom left cells). Row is 'active' relative to column 'control', e.g. WL vs NI/UC (SMD 0.39 [95% CrI 0.06, 0.73]) favours NI/UC relative to WL (SMD >0 favours column).

Targeted Depression: (green shading, top right cells) Column is 'active' relative to row 'control' e.g. WL vs CB (SMD -0.42 [95% CrI -0.69, -0.15]) favours CB relative to WL (SMD <0 favours row)
